# SWISS: multiplexed orthogonal genome editing in plants with a Cas9 nickase and engineered CRISPR RNA scaffolds

**DOI:** 10.1186/s13059-020-02051-x

**Published:** 2020-06-16

**Authors:** Chao Li, Yuan Zong, Shuai Jin, Haocheng Zhu, Dexing Lin, Shengnan Li, Jin-Long Qiu, Yanpeng Wang, Caixia Gao

**Affiliations:** 1 State Key Laboratory of Plant Cell and Chromosome Engineering, Center for Genome Editing, Institute of Genetics and Developmental Biology, Innovation Academy for Seed Design, Chinese Academy of Sciences, Beijing, China; 2 College of Advanced Agricultural Sciences, University of Chinese Academy of Sciences, Beijing, China; 3 State Key Laboratory of Plant Genomics, Institute of Microbiology, Innovation Academy for Seed Design, Chinese Academy of Sciences, Beijing, China

**Keywords:** Multiplexed orthogonal genome editing, Cas9 nickase, CBE, ABE, Indels, RNA scaffolds

## Abstract

We describe here a CRISPR simultaneous and wide-editing induced by a single system (SWISS), in which RNA aptamers engineered in crRNA scaffold recruit their cognate binding proteins fused with cytidine deaminase and adenosine deaminase to Cas9 nickase target sites to generate multiplexed base editing. By using paired sgRNAs, SWISS can produce insertions/deletions in addition to base editing. Rice mutants are generated using the SWISS system with efficiencies of cytosine conversion of 25.5%, adenine conversion of 16.4%, indels of 52.7%, and simultaneous triple mutations of 7.3%. The SWISS system provides a powerful tool for multi-functional genome editing in plants.

## Background

Single-nucleotide substitutions, gene expression changes, or removal of deleterious genes are molecular basis of many important agronomic traits in plants [1]. Stacking traits or changing several key factors of regulatory pathways would greatly advance crop breeding [1]. Diversity and simplicity of CRISPR-Cas systems provide powerful molecular toolboxes [2–10]. Several strategies have been employed to implement multiplex applications in bacteria, yeast, and mammalian cells [11–16]. The most commonly used multiplex strategies for orthogonal genome manipulation include several orthogonal CRISPR systems forming the multi-functional CRISPR system, such as a dual-functional method using SpCas9 variants for adenine base editor (ABE) and SaCas9 for cytosine base editor (CBE) [17] or a tri-functional method using LbCpf1 variant for CRISPRa, SpCas9 variant for CRISPRi, and SaCas9 variant for deletion [15]. However, these strategies require delivering multiple Cas proteins simultaneously, and each Cas protein needs its own PAM recognition [15, 17]. On the other hand, various RNA aptamers were incorporated into CRISPR RNA scaffolds, and these aptamers could recruit their binding proteins to Cas9-targeted sites [14]. This strategy has been used to recruit gene activation or repression effectors to target different genomic sites to perform dual function systems [14], and to recruit sets of fluorescent proteins to label multiplexed genomic site [18]. Nonetheless, tools for performing multi-functional editing are still very limited, especially in plants [1].

**Fig. 1:**
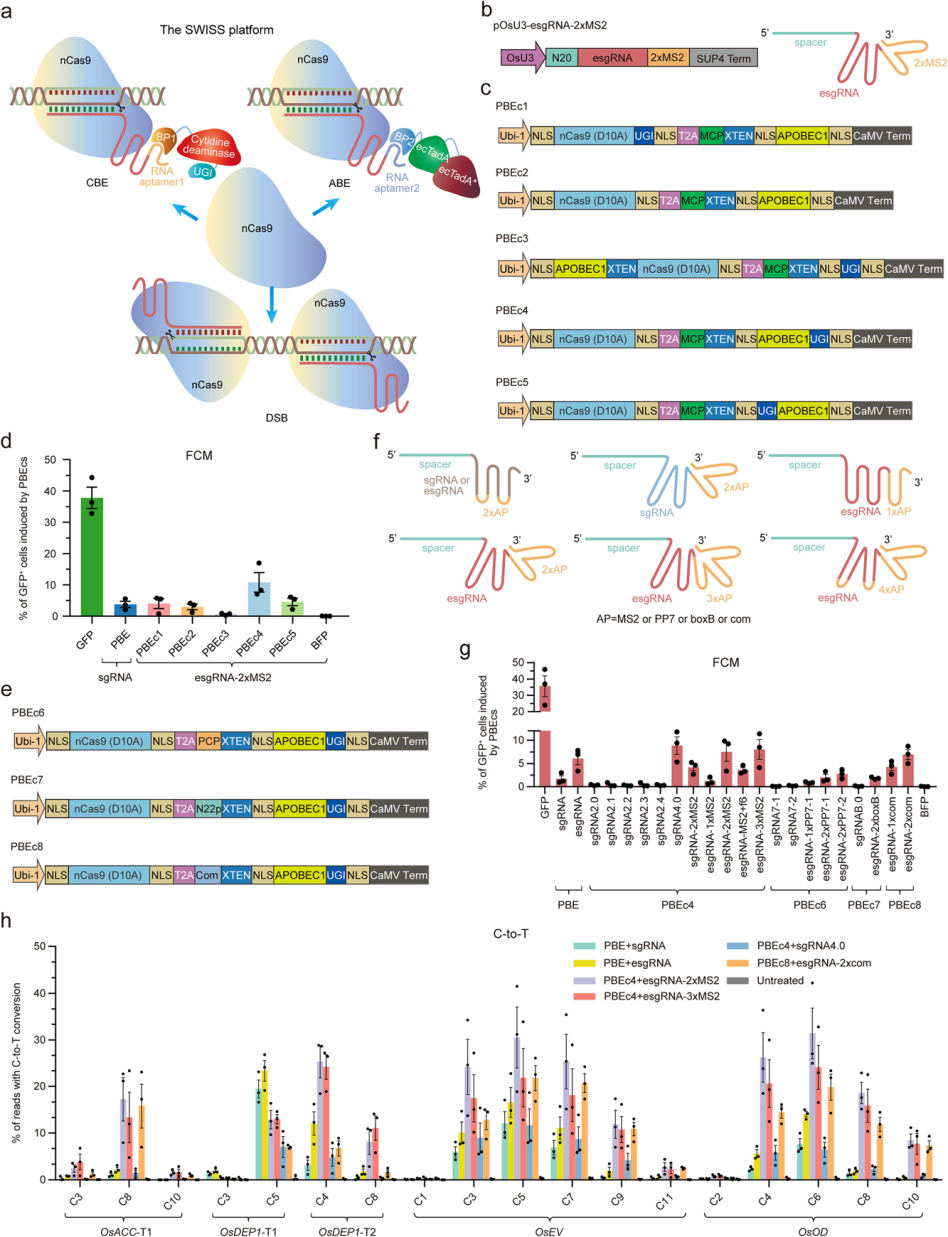
Multiple RNA scaffolds and binding protein orthologs mediate efficient C-to-T conversion. **a** The CRISPR scaffold RNA-programmed multiplex genome editing system based on nCas9 nuclease. Abbreviation: BP, binding protein. **b** Architecture of the pOsU3-esgRNA-2×MS2 construct with two MS2 hairpins at the 3′-end of the esgRNA. Abbreviation: SUP4 Term, transcription terminator for the *S. cerevisiae SUP4* tRNA gene. **c** Architectures of PBEc1-c5. Abbreviations: XTEN, 16-aa linker; NLS, nuclear localization signal; CaMV, cauliflower mosaic virus; Term, terminator. **d** Comparison of C-to-T conversion using a BFP-to-GFP reporter system by PBE and the five PBEcs in rice protoplasts (*n* = 3). Values and error bars indicate means ± s.e.m. of three independent experiments. **e** Architectures of PBEc6-c8. Abbreviations: XTEN, a 16-aa linker; NLS, nuclear localization signal; CaMV, cauliflower mosaic virus; Term, terminator. **f** Schematic of the scRNAs with MS2, PP7, boxB, or com RNA hairpins in the tetraloop and stem loop2 or the 3′-end of the sgRNA and esgRNA. **g** Comparison of C-to-T conversion using the BFP-to-GFP reporter system induced by various scRNAs and their cognate PBEcs in rice protoplasts (*n* = 3). The f6 aptamer hairpin binds MCP specifically. Two PP7 hairpin variants were adopted. Values and error bars indicate means ± s.e.m. of three independent experiments. **h** Comparison of C-to-T editing frequencies of rice endogenous genes induced by four scRNAs and their cognate PBEcs (*n* = 3). An untreated protoplast sample served as control. Values and error bars indicate means ± s.e.m. of three independent experiments.

Compared with Cas9 and dCas9, nCas9 has not been exploited to its full potential for multiplex genome engineering. To generate multiple genetic modifications of plant genomes, we envisioned a multiplex genome editing system that would achieve simultaneous wide-editing induced by a single system (SWISS) based on nCas9 nuclease (Fig. 1a). The system contains sgRNA scaffolds, with different RNA aptamers recruiting cognate binding proteins (BPs), fused with cytidine or adenosine deaminases, and could carry out cytidine base editing and adenine base editing at different target sites simultaneously. By using another pair of sgRNAs to this dual-function system, SWISS could introduce a double-strand break (DSB) at a third target site, obtaining a tri-functional genome editing at multiple sites (Fig. 1a).

## Results

### Engineered constructs and scRNAs for efficient C-to-T conversion

We previously developed a plant cytosine base editor [19], PBE, consisting of cytidine deaminase APOBEC1 [20], nCas9 (D10A), and uracil DNA glycosylase inhibitor (UGI). In the present work, we firstly generated a CRISPR RNA scaffold (scRNA) construct with two MS2 hairpins at the 3′-end of the esgRNA (esgRNA-2×MS2), which mediates efficient activation in human cells [14] and is driven by the *OsU3* promoter (Fig. 1b; Additional file 2: Sequences S1 and S2). To generate MS2-recruited PBE constructs (PBEcs), we used a T2A “self-cleaving” peptide to express nCas9 and MCP (MS2 coat protein)-deaminase fusion modules simultaneously; the nCas9 (D10A) was fused with or without APOBEC1 or UGI as the RNA-programmed module, while MCP fused with APOBEC1 or UGI or both was the recruited module, generating PBEc1-c5. These PBEcs were codon-optimized for crop plants and driven by the *Ubi-1* promoter of maize (Fig. 1c; Additional file 2: Sequences S3).

We tested PBEc1-c5 with the esgRNA-2×MS2 scaffold in our former BFP-to-GFP reporter system using rice protoplasts [19], in which GFP fluorescence requires the codon CAC (His66) to be converted to TAC (Tyr66). PBE together with a conventional sgRNA construct was used as control. The average proportion of GFP^+^ rice cells ranged from 0.7 to 10.8%, with the MCP-APOBEC1-UGI (PBEc4)-recruited module the most efficient and its frequency being about 2.9-fold higher than obtained with PBE (Fig. 1d; Additional file 1: Figure S1), while the yield with MCP-UGI-APOBEC1 (PBEc5) was only about 1.2-fold higher than that with PBE. C-to-T activity using MCP-APOBEC1 as recruited module (PBEc1 and PBEc2) was comparable to that of PBE. However, when MCP-UGI was the recruited module (PBEc3), C-to-T activity declined dramatically (Fig. 1d), though MCP bound to its RNA aptamer hairpin as a dimer [21]. Therefore, we chose the structure of PBEc4 for further development.

To develop a platform for multiplex recruitment and create several scRNAs for efficient C-to-T conversion, we replaced the MCP in PBEc4 with PCP, N22p, and Com [14, 18], generating PBEc6-c8, which recognize the well-characterized viral RNA hairpins PP7, boxB, and com, respectively (Fig. 1e; Additional file 2: Sequences S3). For scRNAs, besides one, two, or three hairpins of these RNA aptamers (MS2, PP7, boxB, or com) were introduced to the 3′-end of the sgRNA or esgRNA [22, 23], RNA hairpins on the tetraloop and stem loop2 of sgRNA or esgRNA and a quad-hairpin scRNA (sgRNA4.0) were generated for comparison [18, 24] (Fig. 1f; Additional file 1: Figure S2; Additional file 2: Sequences S2).

We then compared the activity of these scRNAs and cognate PBEcs using the BFP-to-GFP reporter system in rice protoplasts. To our surprise, we found that all the scRNAs with RNA hairpins on the tetraloop and stem loop2, including the MS2, PP7, and boxB hairpins, yielded very low frequencies of GFP^+^ signals, ranging from 0.1 to 0.4% (Fig. 1g; Additional file 1: Figure S3). On the other hand, all the scRNAs bearing two or three RNA aptamer hairpins at their 3′-ends, and esgRNA-1×com incorporating one 3′-end hairpin, yielded robust frequencies of GFP^+^ signals, ranging from 1.8 to 8.8% (Fig. 1g; Additional file 1: Figure S3). Of them, PBEc4 combined with esgRNA-2×MS2 (7.5%), esgRNA-3×MS2 (8.0%), and sgRNA4.0 (8.8%), and PBEc8 combined with esgRNA-2×com (6.9%), generated even higher frequencies of GFP^+^ signals than the combinations of the PBE and sgRNA (1.7%) or esgRNA (6.0%) (Fig. 1g; Additional file 1: Figure S3). The different outcomes for the two scRNA conformations could be due to the fact that we used a double-stranded linker between hairpin repeats to improve the conformational stability of the 3′-ends of the multi-hairpin scRNAs [14].

To evaluate the effectiveness of esgRNA-2×MS2, esgRNA-3×MS2, sgRNA4.0, and esgRNA-2×com in converting C-to-T in endogenous rice genes, we expressed the sgRNAs in rice protoplasts using these four scRNAs and co-transfected PBEc4 or PBEc8. PBE with conventional sgRNA and esgRNA constructs served as controls. The base editing efficiencies at C_3_ to C_9_ of these five tested target sites (*OsACC*-T1, *OsDEP1*-T1, *OsDEP1*-T2, *OsEV*, and *OsOD*) were enhanced using esgRNA-2×MS2 (average 18.0%), esgRNA-3×MS2 (average 15.0%), and esgRNA-2×com (average 11.1%) compared with the conventional sgRNA (average 4.8%), esgRNA (8.0%), and sgRNA4.0 (average 4.7%) (Fig. 1h). The results with esgRNA-2×MS2, esgRNA-3×MS2, and esgRNA-2×com were 2.3- to 3.8-fold superior to those with the conventional sgRNA (Additional file 1: Figure S4) and had the same primary C-to-T base editing window (Fig. 1h; Additional file 1: Figure S5). Moreover, the C-to-T base editing efficiencies of narrow window APOBEC1 variants (YE1, EE, and YEE) can also be improved 1.4- to 1.8-fold in central positions (C_5_ for *OsEV*, C_6_ for *OsOD*) by esgRNA-2×MS2 with PBEc4 architecture than sgRNA with PBE architecture (Additional file 1: Figure S6; Additional file 2: Sequences S3).

Thus, we have shown that incorporating different RNA aptamers into sgRNA provides an effective approach to multiplex recruitment of RNA-programmed nCas9 (D10A) in plants. In addition, PBEc4 combined with esgRNA-2×MS2 or esgRNA-3×MS2 and PBEc8 combined with esgRNA-2×com can be chosen as candidates for developing multiplex genome editing systems.

### Engineering constructs for RNA scaffolds mediated A-to-G conversion

**Fig. 2:**
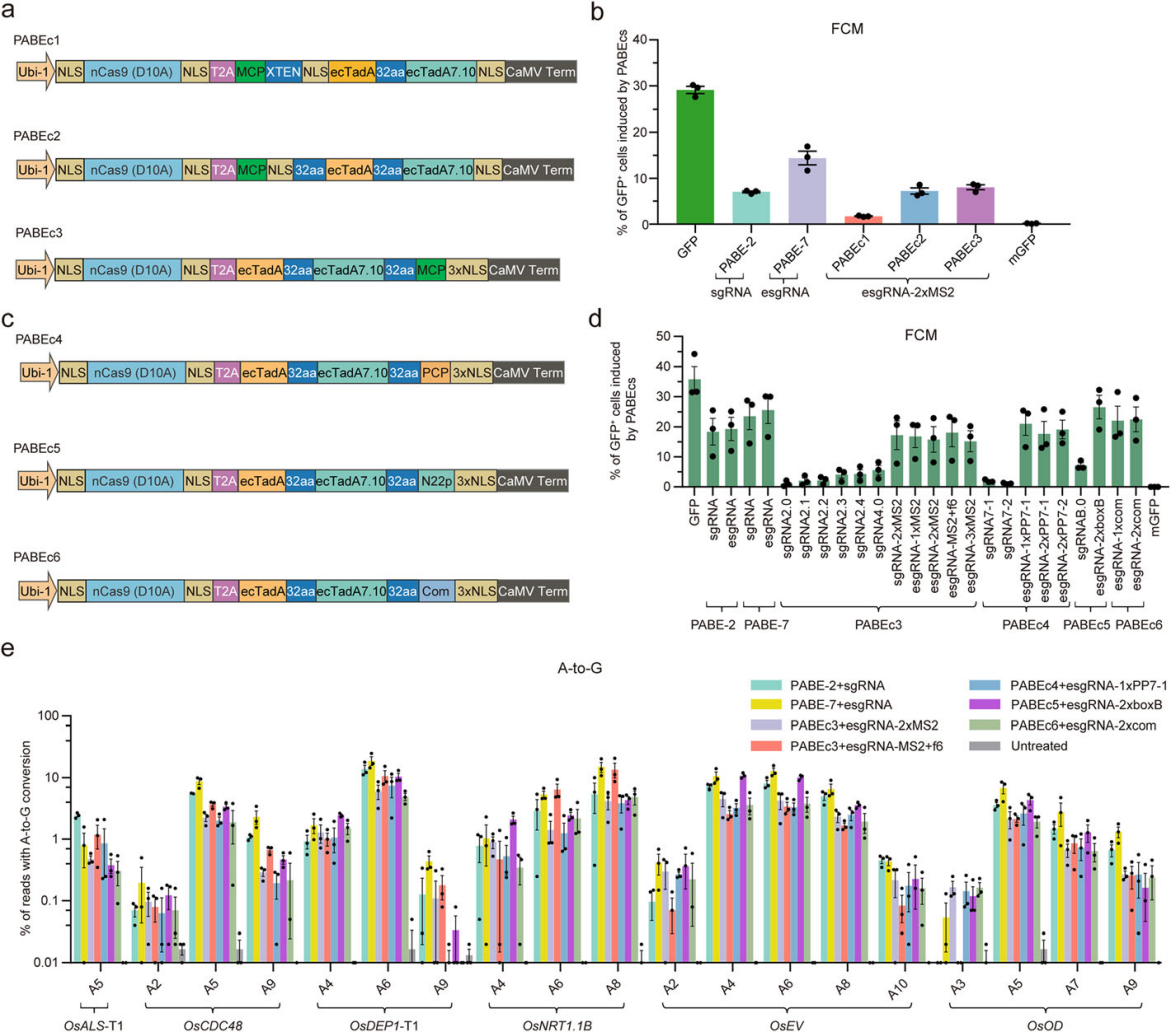
Optimization of plant adenine base editor constructs using multiple RNA scaffolds and binding protein orthologs. **a** Architectures of PABEc1-c3. Abbreviations: ecTadA7.10, evolved *Escherichia coli* TadA; aa, amino acid; XTEN, a 16 aa linker; NLS, nuclear localization signal; CaMV, cauliflower mosaic virus; Term, terminator. **b** Comparison of A-to-G conversion using the mGFP-to-GFP reporter system induced by PABE and the three PABEcs in rice protoplasts (*n* = 3). Values and error bars indicate means ± s.e.m. of three independent experiments. **c** Architectures of PABEc4-c6. Abbreviations: ecTadA7.10, evolved *Escherichia coli* TadA; aa, amino acid; XTEN, a 16 aa linker; NLS, nuclear localization signal; CaMV, cauliflower mosaic virus; Term, terminator. **d** Comparison of A-to-G conversion using the mGFP-to-GFP reporter system induced by various scRNAs and their cognate PABEcs in rice protoplasts (*n* = 3). Values and error bars indicate means ± s.e.m. of three independent experiments. **e** Comparison of the A-to-G editing frequencies of endogenous rice genes induced by five scRNAs and their cognate PABEcs (*n* = 3). An untreated protoplast sample served as control. Values and error bars indicate means ± s.e.m. of three independent experiments

We previously created the plant adenine base editor [25], PABE-7, composed of a laboratory-evolved deoxyadenosine deaminase dimer ecTadA-ecTadA7.10 [26], nCas9 (D10A), and three copies of the SV40 NLS at the C terminus. To repurpose PABE-7 into RNA aptamer-recruiting architecture using nCas9 (D10A) platform, we generated PABE constructs (PABEc1-c3) for recruiting esgRNA-2×MS2 by optimizing the linker length and location between MCP and adenosine deaminase (Fig. 2a; Additional file 2: Sequences S3). The mGFP-to-GFP reporter system was used to test A-to-G conversion activity in rice protoplasts [25]; in this case, an A-to-G conversion on the non-coding strand converts TAG to CAG (Gln69) on the coding strand. In contrast to the increased C-to-T editing efficiency obtained by using PBEc4, the A-to-G editing efficiency of PABEc1-c3 was lower (1.7–8.0%) than that of PABE-7 (14.4%). Of which, PABEc3 showed higher A-to-G editing efficiency (Fig. 2b; Additional file 1: Figure S7). Therefore, we chose the PABEc3 architecture for further multiplex development and tried to improve its activity using other RNA aptamers.

We proceeded to replace the C terminal MCP in PABEc3 with PCP, N22p, and Com, generating PABEc4-c6 (Fig. 2c; Additional file 2: Sequences S3). These cognate scRNAs were tested with PABEc3-c6 using the mGFP-to-GFP reporter system in rice protoplasts. To our surprise, the A-to-G activity of PABEc5 combined with esgRNA-2×boxB (26.5%) was increased and was comparable to that of PABE-7 combined with esgRNA (25.6%) (Fig. 2d; Additional file 1: Figure S8). The highest A-to-G activities in other RNA aptamer groups were PABEc3 combined with esgRNA-MS2+f6 (18.1%), PABEc4 combined with esgRNA-1×PP7-1 (21.0%), and PABEc6 combined with esgRNA-2×com (22.5%); however, they were still lower than that of PABE-7 combined with esgRNA (Fig. 2d; Additional file 1: Figure S8). As observed with the PBEcs using the scRNAs of RNA hairpins in the tetraloop and stem loop2 conformation, the A-to-G editing activities of PABEcs with these scRNAs also induced much lower GFP^+^ signals, ranging from 1.1 to 7.2% (Fig. 2d; Additional file 1: Figure S8).

We chose esgRNA-2×MS2, esgRNA-MS2+f6, esgRNA-1×PP7-1, esgRNA-2×boxB, and esgRNA-2×com to evaluate the effectiveness of PABEcs in converting A-to-G in endogenous rice genes. Six appropriate sgRNAs were inserted into these scRNAs, and they were co-transfected with cognate PABEcs into rice protoplasts (Additional file 1: Table S1). Of these combinations, PABEc5 combined with esgRNA-2×boxB had the highest A-to-G base editing efficiency (average 4.7%) in A_4_ to A_8_, which was lower than PABE-7 combined with esgRNA (average 7.6%), but was comparable to that of PABE-2 combined with sgRNA (average 4.8%), a similar construct used in human cells [26] (Fig. 2e; Additional file 1: Figure S9). Therefore, we selected PABEc5 combined with esgRNA-2×boxB as the scRNA for ABE in the multiplex genome editing system.

### Multiplex genome editing with Cas9 nickase and RNA scaffolds

**Fig. 3:**
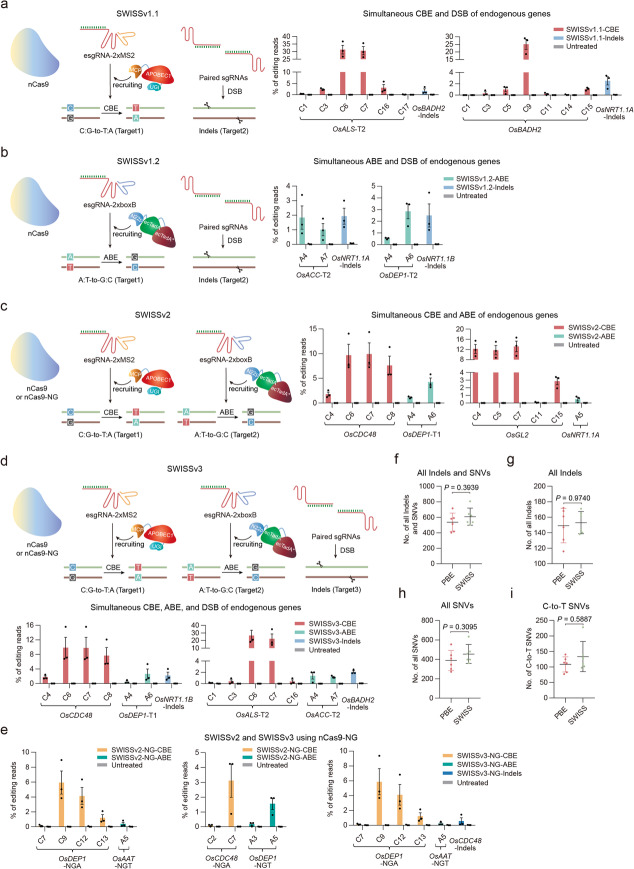
CRISPR RNA scaffold-programmed simultaneous multiplex genome editing based on an nCas9 (D10A) platform in rice protoplasts. **a** Simultaneous CBE and DSB formation induced by PBEc4 with esgRNA-2×MS2, and paired sgRNAs. Left, schematic of the SWISSv1.1 strategy. Right, the two sets of sgRNAs tested (*n* = 3). A CBE target, esgRNA-2×MS2, and paired sgRNAs for creating DSB were assembled in the same vector. **b** Simultaneous ABE and DSB induced by PABEc5 with esgRNA-2×boxB, and paired sgRNAs. Left, schematic of the SWISSv1.2 strategy. Right, the two sets of sgRNAs tested (*n* = 3). One ABE target with esgRNA-2×boxB and paired sgRNAs for creating DSB were assembled in the same vector. **c** Simultaneous CBE and ABE induced by SWISSv2. Left, schematic of the SWISSv2 strategy. Right, the two sets of sgRNAs tested (*n* = 3). One CBE target with esgRNA-2×MS2 and one ABE target with esgRNA-2×boxB were assembled in the same vector. **d** Simultaneous CBE, ABE, and DSB induced by SWISSv3. Top, schematic of the SWISSv3 strategy. Bottom, the two sets of sgRNAs tested (*n* = 3). One CBE target with esgRNA-2×MS2, one ABE target with esgRNA-2×boxB, and paired sgRNAs for DSB were assembled in the same vector. **e** The scope of SWISSv2 and SWISSv3 multiplex genome editing strategies could be expanded by an nCas9-NG PAM variant. The two sets of sgRNAs for SWISSv2 and one set of sgRNA for SWISSv3 tested (*n* = 3). Multiple sgRNAs were assembled in the same vector. **a**–**e** An untreated protoplast sample served as control. Values and error bars indicate means ± s.e.m. of three independent experiments. **f**–**i** Numbers of total indels and SNVs (**f**), total indels (**g**), total SNVs (**h**), and total C-to-T SNVs (**i**) identified in the PBE and SWISS plants. Both of the pH-SWISSv2/v3 and pH-PBE binary constructs were transformed without the sgRNA cassette. Each dot represents the number of indels or SNVs from an individual plant. Horizontal lines and error bars indicate mean number of mutations ± SD (*n* = 6). *P* values were calculated by the two-tailed Mann-Whitney *U* test

The successful development of scRNA-mediated CBE or ABE in rice protoplasts paved the way to multiplexed orthogonal CBE and ABE editing on different targets using the nCas9 (D10A) platform. To fully harness the properties of nCas9 (D10A)-mediated multiplex editing, we first set out to express three sgRNAs in a dual-function system designated as SWISS version 1.1 (SWISSv1.1) based on PBEc4, esgRNA-2×MS2, and paired sgRNAs [27], which should perform simultaneous cytosine base editing and generate paired-nCas9 mediated DSB (Fig. 3a). Toward developing such a platform, we designed two sets of sgRNAs (Additional file 1: Table S2) and assembled multiple sgRNAs in the same vector under the *OsU3* or *TaU6* promoter (Additional file 1: Figure S10; Additional file 2: Sequences S4). C-to-T efficiencies ranged from 0.3 to 31.3% at C_3_ to C_9_, while indels efficiency ranged from 1.7 to 2.5% (Fig. 3a). Encouraged by the activity of SWISSv1.1, we used another dual-function strategy for adenine base editing and simultaneous DSB production based on PABEc5, esgRNA-2×boxB, and paired sgRNAs, designated as SWISS version 1.2 (SWISSv1.2) (Fig. 3b). A-to-G frequencies in the two tested groups reached 2.9%, and indels efficiency reached 2.5% (Fig. 3b). Moreover, with both SWISSv1.1 and SWISSv1.2, more than 79% of the indels reads were deletions induced by the paired nCas9 (D10A) (Additional file 1: Figure S11). These findings establish that scRNA-mediated CBE and ABE can induce multiple sgRNAs to perform base editing and indels dual-function, which shows that the paired nCas9 (D10A) provides an alternative way to induce indels when using PBEc4 and PABEc5.

To determine whether scRNA-mediated base editing can promote dual-function CBE and ABE on different target sites simultaneously when designated as SWISS version 2 (SWISSv2) (Fig. 3c), we co-expressed nCas9 (D10A), MCP-APOBEC1-UGI, and ecTadA-ecTadA7.10-N22p using T2A under one *Ubi-1* promoter (Additional file 1: Figure S12a; Additional file 2: Sequences S5). The esgRNA-2×MS2 for cytosine base editing was installed under the *TaU6* promoter, and the esgRNA-2×boxB for adenine base editing was controlled by the *OsU3* promoter (Additional file 1: Figure S12b). Two groups of targets were tested in rice protoplasts (Additional file 1: Table S2). Amplicon deep sequencing showed that SWISSv2 induced efficient CBE and ABE dual-function on two different targets at both groups. The C-to-T efficiency ranged from 1.8 to 13.2% in C_3_ to C_9_, and the A-to-G efficiency ranged from 0.5 to 4.3% in A_4_ to A_8_ (Fig. 3c).

Encouraged by the results above, we then introduced paired sgRNAs into SWISSv2 designated as SWISS version 3 (SWISSv3) (Fig. 3d) and tested two groups of target sites (Additional file 1: Figure S12c and Table S2). As expected, we observed that SWISSv3 acted as a programmable CBE, ABE, and DSB tri-functional editing system performing C-to-T (0.4–26.7%) and A-to-G (0.5–2.6%) editing on primary editing window of two targets and simultaneously creating indels (2.1–2.2%) at the other target (Fig. 3d). We also compared the editing efficiency of the SWISS system (SWISSv2 and SWISSv3) with PBE, PBEc4, PABE-2, PABEc5, and paired nCas9 (Additional file 1: Figure S13 and Table S2). The results showed that the base editing efficiencies of SWISSv2 and SWISSv3 (average C-to-T 15.3%; average A-to-G 2.0%) were lower than the original PBE (average C-to-T 15.5%) and PABE-2 (average A-to-G 5.1%) base editors, but comparable to the PBEc4 (average C-to-T 15.0%) and PABEc5 (average A-to-G 2.3%) (Additional file 1: Figure S13a, b). The indel efficiency of SWISSv3 (2.1%) was similar to that of paired nCas9 (D10A) (2.1%) (Additional file 1: Figure S13b). These data support that SWISS is a reliable multi-functional genome editing tool.

To release the requirement of PAMs and expand the editing scope of SWISSv2 and SWISSv3, we replaced the nCas9 (D10A) with nCas9-NG (D10A) PAM variant (VRVRFRR) [28], generating the NG version (Additional file 1: Figure S12a; Additional file 2: Sequences S5). Three groups of targets were tested, including two for SWISSv2 and one for SWISSv3 (Additional file 1: Table S2). The C-to-T (0.1–5.9%) and A-to-G (0.2–1.6%) editing on two targets were efficient in SWISSv2 of Cas9-NG PAM variant (Fig. 3e). Similarly, the C-to-T (up to 5.9%), A-to-G (up to 0.3%), and indels (0.6%) editing on three targets were also observed in SWISSv3 of Cas9-NG PAM variant (Fig. 3e). Taken together, SWISSv2 and SWISSv3 provide alternative tools for gene stacking and genetic modification in plants.

### Simultaneous CBE, ABE, and indels formation in rice plants

To test the potential of SWISSv3 in rice plants, we used multiple sgRNAs targeting *OsALS* [29], *OsACC* [30], and *OsBADH2* [31] and assembled into the binary vector (Additional file 1: Figure S14a). The editing targets in regenerated plants were examined by T7EI assay and confirmed by Sanger sequencing (Additional file 1: Figure S14b, c). Efficient C conversion (25.5%), A conversion (16.4%), and indels formation (52.7%) were evident in 55 regenerated rice seedlings (Table 1). Totally, 10 plants (18.1%) were involved in simultaneous dual-function editing on different targets, including 1 plant (1.8%) containing simultaneous C edits and A edits, 7 plants (12.7%) containing simultaneous C edits and indels, and 2 plants (3.6%) containing simultaneous A edits and indels (Table 2). Importantly, 4 plants (7.3%) contained simultaneous C edits, A edits, and indels at separate targets, and SWISSv3 also produced individual editing events with C edits (3.6%), A edits (3.6%), and indels (29.1%) at the three targets (Table 2).

**Table 1:** Mutation frequencies induced by SWISSv3 in T_0_ rice plants

Types of genome editing	Target sites	No. of transgenic rice lines	No. of mutants^a^	Genotype of mutations^b^	Heterozygous/homozygous
CBE	*OsALS*-T2	55	14 (25.5%)	C_6_>T_6_ (7); C_6_>G_6_ (2); C_6_C_7_>T_6_T_7_ (4); C_6_C_7_C_16_>T_6_T_7_T_16_ (1)	14/0
ABE	*OsACC*-T2		9 (16.4%)	A_4_>G_4_ (7); A_7_>G_7_ (2)	9/0
Indels	*OsBADH2*-Indels		29 (52.7%)	deletions (22); insertions (7)	19/10

^a^Based on the number of T_0_ lines (rice) carrying the observed mutations over the total number of T_0_ transgenic rice lines analyzed

^b^The genotypes of indels were analyzed with the online tools DSDecodeM and TIDE (see the “Methods” section)

**Table 2:** Multiplex genome editing using SWISSv3 in T_0_ rice plants

Editing events	Target sites	Genotype of mutations	Efficiencies
Single	*OsALS*-T2	C_6_>T_6_ (1); C_6_C_7_>T_6_T_7_ (1)	3.6% (2/55)
	*OsACC*-T2	A_4_>G_4_ (2)	3.6% (2/55)
	*OsBADH2*-Indels	deletions (12); insertions (4)	29.1% (16/55)
Double	*OsALS*-T2/*OsACC*-T2	C_6_>T_6_+A_4_>G_4_ (1)	1.8% (1/55)
	*OsALS*-T2/*OsBADH2*-Indels	C_6_>T_6_+deletion (2); C_6_>T_6_+insertion (1); C_6_>G_6_+deletion (1); C_6_C_7_>T_6_T_7_+deletion (2); C_6_C_7_C_16_>T_6_T_7_T_16_+insertion (1)	12.7% (7/55)
	*OsACC*-T2/*OsBADH2*-Indels	A_4_>G_4_+deletion (1); A_7_>G_7_+deletion (1)	3.6% (2/55)
Triple	*OsALS*-T2/*OsACC*-T2/*OsBADH2*-Indels	C_6_>T_6_+A_4_>G_4_+ deletion (1); C_6_>G_6_+ A_4_>G_4_+ insertion (1); C_6_>T_6_+A_7_>G_7_+ deletion (1); C_6_C_7_>T_6_T_7_+ A_4_>G_4_+ deletion (1)	7.3% (4/55)

We also evaluated the potential for off-target effects; we searched the genomic sequence for all target sites that contained sequences with up to a 3-nt mismatch and sequenced these sites in the triple mutants. We found no off-target mutations in any of the triple mutants (Additional file 1: Table S3). Previous studies showed that APOBEC1-based CBE induced Cas9-independent genome-wide mutations in rice and mouse [32, 33]. To examine specificity of the SWISS systems, twelve transgenic rice plants expressing the SWISSv2/v3 or PBE without sgRNA were analyzed by whole genome sequencing at an average depth of 60× with high quality (Additional file 1: Table S4). We filtered out background mutations using ten wild-type plants. The results showed that there was no significant difference in the average number of total indels and single-nucleotide variants (SNVs) between the SWISS and PBE groups (Fig. 3f-i). Thus, the genome-wide Cas9-independent off-target effects of the SWISS system was comparable to that of PBE [32].

Taken together, our findings demonstrate that SWISSv3 acts as a tri-functional synthetic programmable genome editing system with CRISPR RNA scaffolds in plants. This ability will facilitate molecular design breeding in crops.

## Discussion

Multiplex genome editing using multiple sgRNAs could be exploited in two ways; one is performing the same type of editing events on different targets [3, 34] and the other is performing a range of different editing events on different targets [11–13, 15]. Although Cas9 or Cas12a has been engineered as dual-functional genome editing system using a truncated sgRNA (or crRNA) and another full-length sgRNA (or crRNA), this strategy was restricted in editing gene regulation and cleave on different targets simultaneously [11–13]. Other multi-functional genome editing could also be achieved with CRISPR-Cas orthologs by recognizing different target sites [15, 17], but large cargo-capacity vector or co-delivery multiple vectors are needed and specific PAM sequences are required. In our SWISS system, we used a single Cas9 nickase (D10A) and different scRNAs for multiplex editing; the reduced vector size and the use of an NG PAM Cas9 variant would further expand the targeting scope of the SWISS systems.

In the studies described above, we used the RNA polymerase III promoters *OsU3* and *TaU6* to express multiple sgRNAs [35, 36]. Other multiple sgRNA strategies, such as using Csy4 RNA ribonuclease [37] and ribozymes to process polycistronic sgRNAs [38], could be incorporated into SWISS systems. Since the average C-to-T activity of scRNA-recruited constructs was higher than that of PBE, this strategy could be used to improve the activity of narrow window cytidine deaminase variants. Based on this result, we speculate that the RNA scaffolds could also recruit other deaminase variants with higher efficiencies and specificities to avoid the unpredictable DNA and RNA off-target mutations [39, 40]. Although the A-to-G activity of the scRNA-recruited construct was comparable to that of PABE-2 on endogenous rice targets, it was efficient enough in SWISSv3 for us to obtain A-to-G substitutions in rice plants. At the same time, we tested different PABE constructs and various RNA aptamers, but unlike the PBEcs, they still had low A-to-G activity than the optimized PABE-7 construct [25]. Combined with the observation of lower A-to-G activity in our STEME systems [41], we speculate that the opportunities to optimize the architectures of PABEcs are limited in plants. One possible way to improve the A-to-G activity in plants is to incorporate the recently evolved ecTadA variants with improved A-to-G editing efficiency, such as the adenosine deaminase in ABE8e and ABE8s [42, 43]. Another possible way is to fuse a synthetic transcription activation domain for opening chromatin to improve the A>G editing efficiency [44].

Although dual-function SWISSv1.1 and SWISSv1.2 systems may also be implemented with canonical CBEs and ABEs using multiple sgRNAs, our RNA aptamer recruitment strategy provides a better alternative, especially if multiplex editing was performed in nCas9 (D10A)-over-expressing plants. Hence, we propose to create an nCas9 (D10A)-over-expressing rice strain, as a developmental platform for operating SWISS systems. By introducing a small cargo carrying multiple sgRNAs and the base editing recruitment modules in the twice successive transformation, multi-functional editing events could also be achieved. Moreover, using a third RNA scaffold with a truncated protospacer (14–15 nt) to recruit an epigenetic effector, repressor, activator, or fluorescent marker would create a quadruple-function CRISPR system.

Although orthogonal elements have been widely used in the field of synthetic biology in organism like bacteria, yeasts, and mammalian cells, the utilization of such kind of elements in plant has been very limited. It is worth noting that the orthogonal aptamer-binding protein pairs exploited in our study and by others [45, 46] can expand the elements stock in plant synthetic biology as well. The efficient, specific, and orthogonal binding between the aptamer and its corresponding binding protein that examined in our work may contribute to the construction of synthetic signal pathways, gene circuits, or biosensor in plant cells.

## Conclusions

We describe here an efficient multiplexed orthogonal genome editing strategy using Cas9 nickase and CRISPR RNA scaffolds in rice. The scRNAs of 3′-end RNA aptamer hairpins esgRNA-2×MS2 and esgRNA-2×boxB were used for mediating cytosine base editing and adenine base editing, respectively. Therefore, in our SWISS systems, we used MS2 and boxB to achieve multiplexed orthogonal CBE and ABE editing on different targets. We also took advantage of the nickase activity of nCas9 (D10A) to produce DSB with paired sgRNAs on the third target, generating SWISSv3, and realized multiplexed orthogonal CBE, ABE, and indels mutations on different targets, which also worked efficiently in rice plants. These findings expand the multiplex genome editing tools in plants and provide feasible opportunities for traits stacking and improvement in plants.

## Methods

### Plasmid construction

The nCas9, APOBEC1, UGI, ecTadA, and ecTadA7.10 portions of PBEcs and PABEcs were amplified from PBE or PABE-7 [19, 25]. The Cas9 variant nCas9-NG (D10A) containing the R1335V/L1111R/D1135V/G1218R/E1219F/A1322R/T1337R substitutions was amplified from STEME-NG [41]. Binding proteins, including MCP, PCP, N22p, and Com, were synthesized commercially (GenScript, Nanjing, China). The different components of PBEcs, PABEcs, and SWISSv2/v3 were assembled into the pJIT163 backbone by One Step Cloning (ClonExpress II One Step Cloning Kit, Vazyme, Nanjing, China). The sgRNA constructs pOsU3-sgRNA and pOsU3-esgRNA have been previously described [25]; the *TaU6* promoter was amplified from pTaU6-sgRNA [25]. All the scRNAs listed in Additional file 2: Sequences S2 were synthesized commercially and used to replace the sgRNA in pOsU3-esgRNA by One Step Cloning. Annealed oligos were inserted into *Bsa*I (New England BioLabs)-digested OsU3-derived vectors. To construct the pH-SWISSv2/v3 binary vector, this cassette was cloned into the pHUE411 backbone under the *Ubi-1* promoter of maize [47]. PCR was performed using TransStart FastPfu DNA Polymerase (TransGen Biotech, Beijing, China). All primer sets used in this work were listed in Additional file 1: Table S5 and were synthesized by Beijing Genomics Institute (BGI).

### Installing multiple sgRNAs

The templates for assembling multiple sgRNAs listed in Additional file 2: Sequences S4 were synthesized commercially. For dual sgRNAs, the PCR products were amplified from esgRNA-pTaU6 or esgRNA-2×boxB-pTaU6 and installed into pOsU3-esgRNA or pOsU3-esgRNA-2×MS2 by Golden Gate cloning [37]. For triple or quadruple sgRNAs, the PCR products were amplified from single- or dual-sgRNA vectors and assembled by Multi One Step Cloning (ClonExpress MultiS One Step Cloning Kit, Vazyme, Nanjing, China).

### Protoplast transfection

We used the *Japonica* rice variety Nipponbare to prepare protoplasts. Protoplast isolation and transformation were performed as described [48]. Ten micrograms of each of nuclease and sgRNA plasmid DNA was introduced into the protoplasts by PEG-mediated transfection, with a mean transformation efficiency of 30–45% as measured by flow cytometry (FCM) or hemocytometer. The transfected protoplasts were incubated at 23 °C. The protoplasts were collected after 60 h incubation, and the genomic DNA were extracted for amplicon deep sequencing.

### *Agrobacterium*-mediated transformation of rice callus cells

The binary vector was transformed into *A. tumefaciens* AGL1 by electroporation and used to transform about 120 rice calli. *Agrobacterium*-mediated transformation of callus cells of the *Japonica* rice variety Zhonghua11 was conducted as reported [48]. Hygromycin (50 μg/ml) was used to select transgenic plants.

### DNA extraction

Genomic DNA of protoplasts was extracted with a DNA-Quick Plant System (Tiangen Biotech, Beijing, China). Genomic DNA of regenerated rice seedlings was extracted with CTAB. Targeted sites were amplified with specific primers, and the amplicons were purified with an EasyPure PCR Purification Kit (TransGen Biotech, Beijing, China) and quantified with a NanoDrop™ 2000 Spectrophotometer (Thermo Fisher Scientific, Waltham, MA, USA).

### Amplicon deep sequencing and data analysis

Genomic DNA extracted from the desired protoplast samples at 60 h post-transfection served as template. Two rounds of PCR were performed for the amplicons of protoplasts. In the first round, the target region was amplified using site-specific primers (Additional file 1: Table S5). In the second, both forward and reverse barcodes were added to the ends of the PCR products for library construction (Additional file 1: Table S5). Equal amounts of the PCR products were pooled and purified by Gel DNA Extraction, and samples were sequenced commercially (Novogene, Tianjin, China) using the Illumina NextSeq 500 platform. The protospacer sequences in the reads were examined to identify base substitutions or indels. Amplicon sequencing was performed three times for each target site, using genomic DNA extracted from three independent protoplast samples. Amplicon reads with a quality score < 30 were filtered out. Analyses of base-editing processivity were performed as previously described [49].

### Mutant identification by T7EI and Sanger sequencing

Site-specific primers were used to amplify genomic DNA from regenerated rice seedlings. T7EI enzyme (Vazyme, Nanjing, China) was used to identify rice mutants with C-to-T conversions, A to G conversions, or indels in target regions, with an optimized protocol: 200 ng sample PCR products, 200 ng wild-type PCR products, and reaction buffer added to 8.0 μL; 98 °C denaturation for 10 min, 95 °C denaturation for 5 min, and temperature lowered from 90 to 10 °C in next 10 steps followed by denaturation for 1 min; 0.2 μL T7EI (10 U/μL) added with water to 10.0 μL; and incubate at 37 °C for 20 min and assay immediately. Sanger sequencing was performed to confirm genotypes. The genotypes of indels were analyzed with the online tools DSDecodeM [50] and TIDE [51].

### Off-target analysis

Potential off-target sites were predicted using the online tool Cas-OFFinder [52]. Sites containing up to 3-nt mismatches were examined. The whole-genome sequencing assay and genome-wide Cas9-independent off-target analysis were conducted as reported [32].

### Statistical analysis

GraphPad Prism version 7.0 was used for all data analysis. All numerical values are presented as means ± s.e.m. Statistical comparison adjustments were performed using two-tailed Mann-Whitney *U* test.

## Supplementary information

**Additional file 1: Figure S1.** Flow cytometry of BFP-to-GFP conversion induced by PBE and the five PBEcs in rice protoplasts. **Figure S2.** Engineering the secondary structures of CRISPR RNA scaffolds. **Figure S3.** Flow cytometry of BFP-to-GFP conversion induced by various scRNAs and their cognate PBEcs in rice protoplasts. **Figure S4.** Frequencies of base editing of endogenous genes by different scRNAs and cognate PBEcs in rice protoplasts. **Figure S5.** Activities of esgRNA-2×MS2, esgRNA-3×MS2, sgRNA4.0, and esgRNA-2×com with cognate PBEcs in rice protoplasts. **Figure S6.** C-to-T editing frequencies generated by scaffold RNA-recruited APOBEC1 narrow-window variants in rice protoplasts. **Figure S7.** Flow cytometry of mGFP-to-GFP conversion induced by PABE and the three PABEcs in rice protoplasts. **Figure S8.** Flow cytometry of mGFP-to-GFP conversion induced by various scRNAs and their cognate PABEcs in rice protoplasts. **Figure S9.** Activities of the selected scaffold RNAs with their cognate PABEcs in rice protoplasts. **Figure S10.** Schematic of multiple sgRNAs assembly for SWISSv1.1 and SWISSv1.2. **Figure S11.** The distributions of deletion reads among the indel sequencing reads for SWISSv1.1, SWISSv1.2, and SWISSv3. **Figure S12.** Schematic of multiple sgRNAs assembly for SWISSv2 and SWISSv3. **Figure S13.** Comparison of the editing efficiencies between the SWISS systems and the individual genome editing tools (PBE, PBEc4, PABE-2, PABEc5, and paired nCas9). **Figure S14.** Simultaneous CBE, ABE, and DSB formation in rice plants. **Table S1.** The sgRNA sequences used to compare the activities of PBEcs and PABEcs. **Table S2.** The sgRNA sequences used for SWISSv1.1, SWISSv1.2, SWISSv2, and SWISSv3 editing in rice protoplasts. **Table S3.** Potential off-target sites analyzed for *OsALS*-T2, *OsACC*-T2, *OsBADH2*-Indels-sgL, and *OsBADH2*-Indels-sgR triple mutants. **Table S4.** Statistics of whole genome sequencing analysis. **Table S5.** Primer sequences used in this study.

**Additional file 2: Sequences S1.** DNA sequences of the *OsU3* and *TaU6* promoters used in this study. **Sequences S2.** DNA sequences of sgRNA, esgRNA, and scRNAs used in this study. **Sequences S3.** DNA sequences of the modules composed of PBEcs and PABEcs used in this study. **Sequences S4.** DNA sequences of templates for assemble multiple sgRNAs used in this study. **Sequences S5.** DNA sequences of SWISSv2/v3 and SWISSv2/v3-NG used in this study.

**Additional file 3.** Review history.

## Data Availability

Deep sequencing data are available under BioProject ID PRJNA628139 (https://www.ncbi.nlm.nih.gov/bioproject/PRJNA628139) [53]. Whole genome sequencing data are available under BioProject ID PRJNA636218 (https://www.ncbi.nlm.nih.gov/bioproject/PRJNA636218) [54].

## References

[1] Chen K, Wang Y, Zhang R, Zhang H, Gao C (2019). CRISPR/Cas genome editing and precision plant breeding in agriculture. Annu Rev Plant Biol.

[2] Jinek M, Chylinski K, Fonfara I, Hauer M, Doudna JA, Charpentier E (2012). A programmable dual-RNA-guided DNA endonuclease in adaptive bacterial immunity. Science.

[3] Cong L, Ran FA, Cox D, Lin S, Barretto R, Habib N, Hsu PD, Wu X, Jiang W, Marraffini LA (2013). Multiplex genome engineering using CRISPR/Cas systems. Science.

[4] Kleinstiver BP, Pattanayak V, Prew MS, Tsai SQ, Nguyen NT, Zheng Z, Joung JK (2016). High-fidelity CRISPR-Cas9 nucleases with no detectable genome-wide off-target effects. Nature.

[5] Zetsche B, Gootenberg JS, Abudayyeh OO, Slaymaker IM, Makarova KS, Essletzbichler P, Volz SE, Joung J, Van Der Oost J, Regev A (2015). Cpf1 is a single RNA-guided endonuclease of a class 2 CRISPR-Cas system. Cell.

[6] Kleinstiver BP, Sousa AA, Walton RT, Tak YE, Hsu JY, Clement K, Welch MM, Horng JE, Malagon-Lopez J, Scarfò I (2019). Engineered CRISPR-Cas12a variants with increased activities and improved targeting ranges for gene, epigenetic and base editing. Nat Biotechnol.

[7] Abudayyeh OO, Gootenberg JS, Essletzbichler P, Han S, Joung J, Belanto JJ, Verdine V, Cox DBT, Kellner MJ, Regev A (2017). RNA targeting with CRISPR-Cas13. Nature.

[8] Cox DB, Gootenberg JS, Abudayyeh OO, Franklin B, Kellner MJ, Joung J, Zhang F (2017). RNA editing with CRISPR-Cas13. Science.

[9] Yan WX, Hunnewell P, Alfonse LE, Carte JM, Keston-Smith E, Sothiselvam S, Garrity AJ, Chong S, Makarova KS, Koonin EV (2019). Functionally diverse type V CRISPR-Cas systems. Science.

[10] Strecker J, Ladha A, Gardner Z, Schmid-Burgk JL, Makarova KS, Koonin EV, Zhang F (2019). RNA-guided DNA insertion with CRISPR-associated transposases. Science.

[11] Dahlman JE, Abudayyeh OO, Joung J, Gootenberg JS, Zhang F, Konermann S (2015). Orthogonal gene knockout and activation with a catalytically active Cas9 nuclease. Nat Biotechnol.

[12] Kiani S, Chavez A, Tuttle M, Hall RN, Chari R, Ter-Ovanesyan D, Qian J, Pruitt BW, Beal J, Vora S (2015). Cas9 gRNA engineering for genome editing, activation and repression. Nat Methods.

[13] Campa CC, Weisbach NR, Santinha AJ, Incarnato D, Platt RJ (2019). Multiplexed genome engineering by Cas12a and CRISPR arrays encoded on single transcripts. Nat Methods.

[14] Zalatan JG, Lee ME, Almeida R, Gilbert LA, Whitehead EH, La Russa M, Tsai JC, Weissman JS, Dueber JE, Qi LS (2015). Engineering complex synthetic transcriptional programs with CRISPR RNA scaffolds. Cell.

[15] Lian J, HamediRad M, Hu S, Zhao H (2017). Combinatorial metabolic engineering using an orthogonal tri-functional CRISPR system. Nat Commun.

[16] Tak YE, Kleinstiver BP, Nuñez JK, Hsu JY, Horng JE, Gong J, Weissman JS, Joung JK (2017). Inducible and multiplex gene regulation using CRISPR–Cpf1-based transcription factors. Nat Methods.

[17] Liu Z, Lu Z, Yang G, Huang S, Li G, Feng S, Liu Y, Li J, Yu W, Zhang Y (2018). Efficient generation of mouse models of human diseases via ABE- and BE-mediated base editing. Nat Commun.

[18] Ma H, Tu L-C, Naseri A, Huisman M, Zhang S, Grunwald D, Pederson T (2016). Multiplexed labeling of genomic loci with dCas9 and engineered sgRNAs using CRISPRainbow. Nat Biotechnol.

[19] Zong Y, Wang Y, Li C, Zhang R, Chen K, Ran Y, Qiu J-L, Wang D, Gao C (2017). Precise base editing in rice, wheat and maize with a Cas9-cytidine deaminase fusion. Nat Biotechnol.

[20] Komor AC, Kim YB, Packer MS, Zuris JA, Liu DR (2016). Programmable editing of a target base in genomic DNA without double-stranded DNA cleavage. Nature.

[21] Chao JA, Patskovsky Y, Almo SC, Singer RH (2008). Structural basis for the coevolution of a viral RNA–protein complex. Nat Struct Mol Biol.

[22] Chen B, Gilbert LA, Cimini BA, Schnitzbauer J, Zhang W, Li G-W, Park J, Blackburn EH, Weissman JS, Qi LS (2013). Dynamic imaging of genomic loci in living human cells by an optimized CRISPR/Cas system. Cell.

[23] Dang Y, Jia G, Choi J, Ma H, Anaya E, Ye C, Shankar P, Wu H (2015). Optimizing sgRNA structure to improve CRISPR-Cas9 knockout efficiency. Genome Biol.

[24] Konermann S, Brigham MD, Trevino AE, Joung J, Abudayyeh OO, Barcena C, Hsu PD, Habib N, Gootenberg JS, Nishimasu H (2015). Genome-scale transcriptional activation by an engineered CRISPR-Cas9 complex. Nature.

[25] Li C, Zong Y, Wang Y, Jin S, Zhang D, Song Q, Zhang R, Gao C (2018). Expanded base editing in rice and wheat using a Cas9-adenosine deaminase fusion. Genome Biol.

[26] Gaudelli NM, Komor AC, Rees HA, Packer MS, Badran AH, Bryson DI, Liu DR (2017). Programmable base editing of A• T to G• C in genomic DNA without DNA cleavage. Nature.

[27] Ran FA, Hsu PD, Lin C-Y, Gootenberg JS, Konermann S, Trevino AE, Scott DA, Inoue A, Matoba S, Zhang Y (2013). Double nicking by RNA-guided CRISPR Cas9 for enhanced genome editing specificity. Cell.

[28] Nishimasu H, Shi X, Ishiguro S, Gao L, Hirano S, Okazaki S, Noda T, Abudayyeh OO, Gootenberg JS, Mori H (2018). Engineered CRISPR-Cas9 nuclease with expanded targeting space. Science.

[29] Mazur BJ, Chui CF, Smith JK (1987). Isolation and characterization of plant genes coding for acetolactate synthase, the target enzyme for two classes of herbicides. Plant Physiol.

[30] Powles SB, Yu Q (2010). Evolution in action: plants resistant to herbicides. Annu Rev Plant Biol.

[31] Shan Q, Zhang Y, Chen K, Zhang K, Gao C (2015). Creation of fragrant rice by targeted knockout of the Os BADH 2 gene using TALEN technology. Plant Biotechnol J.

[32] Jin S, Zong Y, Gao Q, Zhu Z, Wang Y, Qin P, Liang C, Wang D, Qiu J-L, Zhang F, Gao C (2019). Cytosine, but not adenine, base editors induce genome-wide off-target mutations in rice. Science.

[33] Zuo E, Sun Y, Wei W, Yuan T, Ying W, Sun H, Yuan L, Steinmetz LM, Li Y, Yang H (2019). Cytosine base editor generates substantial off-target single-nucleotide variants in mouse embryos. Science.

[34] Zetsche B, Heidenreich M, Mohanraju P, Fedorova I, Kneppers J, DeGennaro EM, Winblad N, Choudhury SR, Abudayyeh OO, Gootenberg JS (2017). Multiplex gene editing by CRISPR–Cpf1 using a single crRNA array. Nat Biotechnol.

[35] Zhang D, Zhang H, Li T, Chen K, Qiu J-L, Gao C (2017). Perfectly matched 20-nucleotide guide RNA sequences enable robust genome editing using high-fidelity SpCas9 nucleases. Genome Biol.

[36] Hao Y, Zong W, Zeng D, Han J, Chen S, Tang J, Zhao Z, Li X, Ma K, Xie X. Shortened snRNA promoters for efficient CRISPR/Cas-based multiplex genome editing in monocot plants. Sci China Life Sci. 2020. 10.1007/s11427-019-1612-6. 10.1007/s11427-019-1612-6 31942685

[37] Čermák T, Curtin SJ, Gil-Humanes J, Čegan R, Kono TJ, Konečná E, Belanto JJ, Starker CG, Mathre JW, Greenstein RL (2017). A multipurpose toolkit to enable advanced genome engineering in plants. Plant Cell.

[38] Gao Y, Zhao Y (2014). Self-processing of ribozyme-flanked RNAs into guide RNAs in vitro and in vivo for CRISPR-mediated genome editing. J Integr Plant Biol.

[39] Zhou C, Sun Y, Yan R, Liu Y, Zuo E, Gu C, Han L, Wei Y, Hu X, Zeng R (2019). Off-target RNA mutation induced by DNA base editing and its elimination by mutagenesis. Nature.

[40] Grünewald J, Zhou R, Iyer S, Lareau CA, Garcia SP, Aryee MJ, Joung JK (2019). CRISPR DNA base editors with reduced RNA off-target and self-editing activities. Nat Biotechnol.

[41] Li C, Zhang R, Meng X, Chen S, Zong Y, Lu C, Qiu J-L, Chen Y-H, Li J, Gao C. Targeted, random mutagenesis of plant genes with dual cytosine and adenine base editors. Nat Biotechnol. 2020. 10.1038/s41587-019-0393-7. 10.1038/s41587-019-0393-7 31932727

[42] Richter MF, Zhao KT, Eton E, Lapinaite A, Newby GA, Thuronyi BW, Wilson C, Koblan LW, Zeng J, Bauer DE, et al. Phage-assisted evolution of an adenine base editor with improved Cas domain compatibility and activity. Nat Biotechnol. 2020. 10.1038/s41587-020-0453-z. 10.1038/s41587-020-0453-z PMC7357821 32433547

[43] Gaudelli NM, Lam DK, Rees HA, Solá-Esteves NM, Barrera LA, Born DA, Edwards A, Gehrke JM, Lee S-J, Liquori AJ, et al. Directed evolution of adenine base editors with increased activity and therapeutic application. Nat Biotechnol. 2020. 10.1038/s41587-020-0491-6. 10.1038/s41587-020-0491-6 32284586

[44] Liu G, Yin K, Zhang Q, Gao C, Qiu J-L (2019). Modulating chromatin accessibility by transactivation and targeting proximal dsgRNAs enhances Cas9 editing efficiency in vivo. Genome Biol.

[45] Li Z, Zhang D, Xiong X, Yan B, Xie W, Sheen J, Li J-F (2017). A potent Cas9-derived gene activator for plant and mammalian cells. Nat Plants.

[46] Lowder LG, Zhou J, Zhang Y, Malzahn A, Zhong Z, Hsieh T-F, Voytas DF, Zhang Y, Qi Y (2018). Robust transcriptional activation in plants using multiplexed CRISPR-Act2.0 and mTALE-Act systems. Mol Plant.

[47] Xing HL, Dong L, Wang ZP, Zhang HY, Han CY, Liu B, Wang XC, Chen QJ (2014). A CRISPR/Cas9 toolkit for multiplex genome editing in plants. BMC Plant Biol.

[48] Shan Q, Wang Y, Chen K, Liang Z, Li J, Zhang Y, Zhang K, Liu J, Voytas DF, Zheng X (2013). Rapid and efficient gene modification in rice and Brachypodium using TALENs. Mol Plant.

[49] Clement K, Rees H, Canver MC, Gehrke JM, Farouni R, Hsu JY, Cole MA, Liu DR, Joung JK, Bauer DE (2019). CRISPResso2 provides accurate and rapid genome editing sequence analysis. Nat Biotechnol.

[50] Liu W, Xie X, Ma X, Li J, Chen J, Liu Y-G (2015). DSDecode: a web-based tool for decoding of sequencing chromatograms for genotyping of targeted mutations. Mol Plant.

[51] Brinkman EK, Chen T, Amendola M, van Steensel B (2014). Easy quantitative assessment of genome editing by sequence trace decomposition. Nucleic Acids Res.

[52] Bae S, Park J, Kim J-S. Cas-OFFinder: a fast and versatile algorithm that searches for potential off-target sites of Cas9 RNA-guided endonucleases. Bioinformatics. 2014;30:1473–5. 10.1093/bioinformatics/btu048 PMC4016707 24463181

[53] Li C, Zong Y, Jin S, Zhu Hao, Lin D, Li S, Qiu J-L, Wang Y, Gao C. SWISS: multiplexed orthogonal genome editing in plants with a Cas9 nickase and engineered CRISPR RNA scaffolds. Dataset. NCBI https://www.ncbi.nlm.nih.gov/bioproject/PRJNA628139 (2020). Accessed 26 Apr 2020. 10.1186/s13059-020-02051-x PMC7296638 32546280

[54] Li C, Zong Y, Jin S, Zhu Hao, Lin D, Li S, Qiu J-L, Wang Y, Gao C. SWISS: multiplexed orthogonal genome editing in plants with a Cas9 nickase and engineered CRISPR RNA scaffolds. Dataset. NCBI https://www.ncbi.nlm.nih.gov/bioproject/PRJNA636218 (2020). Accessed 02 Jun 2020. 10.1186/s13059-020-02051-x PMC7296638 32546280

