# Supplementary material for: SWISS: multiplexed orthogonal genome editing in plants with a Cas9 nickase and engineered CRISPR RNA scaffolds
Source: Genome Biol. 2020 Jun 16;21:141. doi: 10.1186/s13059-020-02051-x (PMC7296638; doi:10.1186/s13059-020-02051-x)
Supplement: Supplementary file 1 — Additional file 1: Figure S1. Flow cytometry of BFP-to-GFP conversion induced by PBE and the five PBEcs in rice protoplasts. Figure S2. Engineering the secondary structures of CRISPR RNA scaffolds. Figure S3. Flow cytometry of BFP-to-GFP conversion induced by various scRNAs and their cognate PBEcs in rice protoplasts. Figure S4. Frequencies of base editing of endogenous genes by different scRNAs and cognate PBEcs in rice protoplasts. Figure S5. Activities of esgRNA-2×MS2, esgRNA-3×MS2, sgRNA4.0, and esgRNA-2×com with cognate PBEcs in rice protoplasts. Figure S6. C-to-T editing frequencies generated by scaffold RNA-recruited APOBEC1 narrow-window variants in rice protoplasts. Figure S7. Flow cytometry of mGFP-to-GFP conversion induced by PABE and the three PABEcs in rice protoplasts. Figure S8. Flow cytometry of mGFP-to-GFP conversion induced by various scRNAs and their cognate PABEcs in rice protoplasts. Figure S9. Activities of the selected scaffold RNAs with their cognate PABEcs in rice protoplasts. Figure S10. Schematic of multiple sgRNAs assembly for SWISSv1.1 and SWISSv1.2. Figure S11. The distributions of deletion reads among the indel sequencing reads for SWISSv1.1, SWISSv1.2, and SWISSv3. Figure S12. Schematic of multiple sgRNAs assembly for SWISSv2 and SWISSv3. Figure S13. Comparison of the editing efficiencies between the SWISS systems and the individual genome editing tools (PBE, PBEc4, PABE-2, PABEc5, and paired nCas9). Figure S14. Simultaneous CBE, ABE, and DSB formation in rice plants. Table S1. The sgRNA sequences used to compare the activities of PBEcs and PABEcs. Table S2. The sgRNA sequences used for SWISSv1.1, SWISSv1.2, SWISSv2, and SWISSv3 editing in rice protoplasts. Table S3. Potential off-target sites analyzed for OsALS-T2, OsACC-T2, OsBADH2-Indels-sgL, and OsBADH2-Indels-sgR triple mutants. Table S4. Statistics of whole genome sequencing analysis. Table S5. Primer sequences used in this study. [file 13059_2020_2051_MOESM1_ESM.docx]

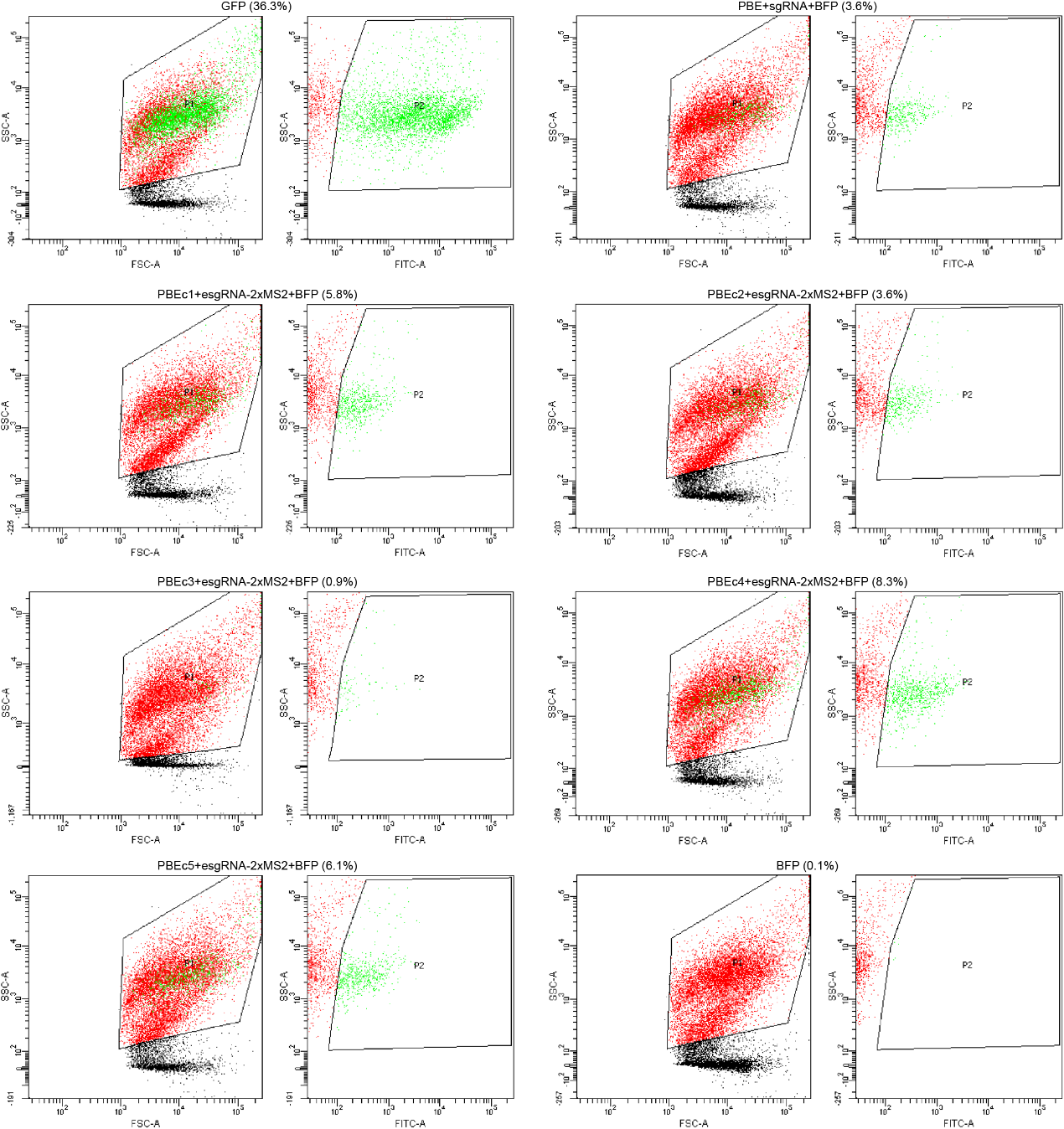


**Figure S1. Flow cytometry of BFP-to-GFP conversion induced by PBE and the five PBEcs in rice protoplasts.** One of three independent biological replicates is shown.


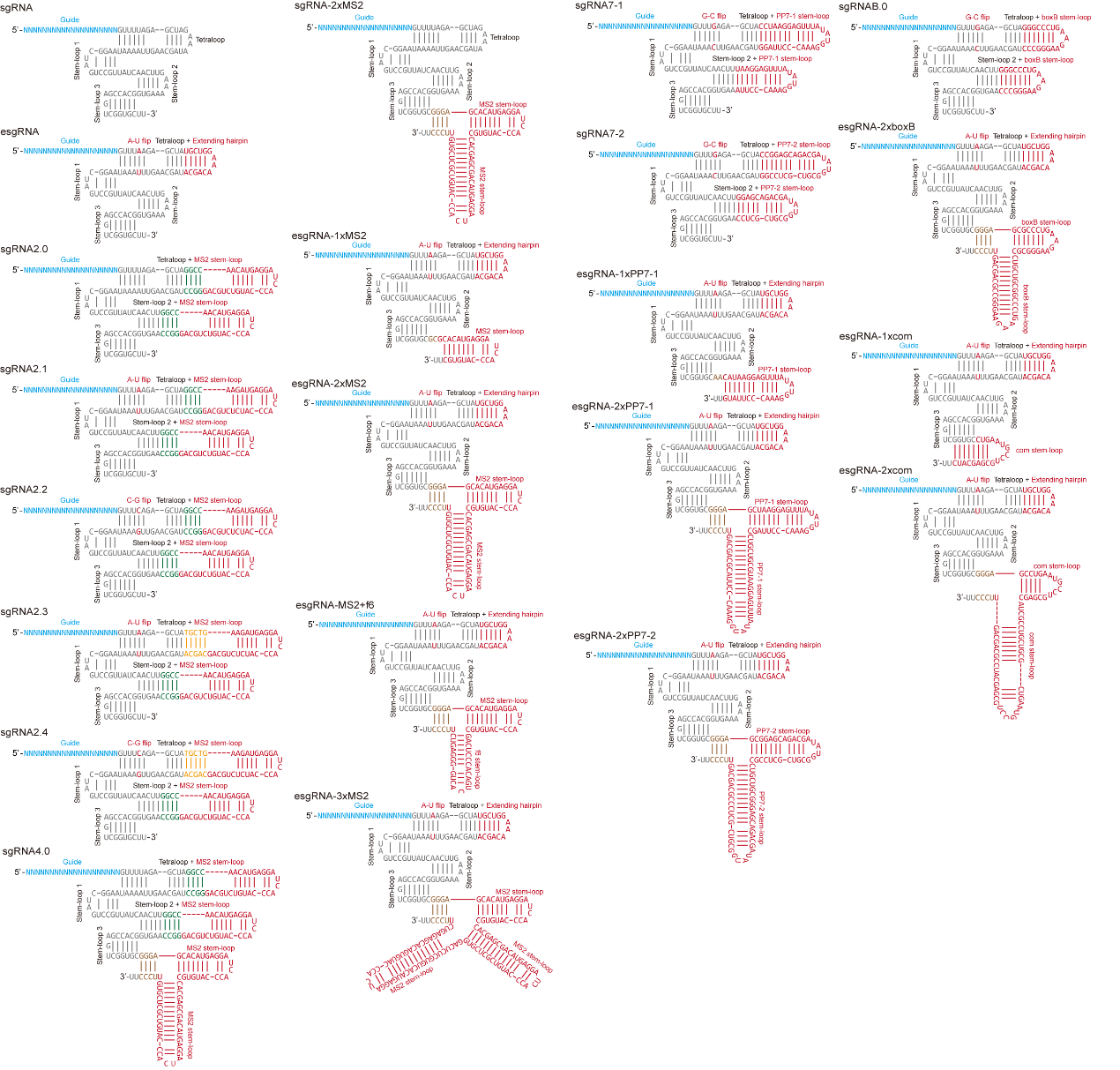


**Figure S2. Engineering the secondary structures of CRISPR RNA scaffolds.** Four RNA aptamers were used, including MS2, PP7, boxB, and com. The scRNAs of sgRNA2.0 to sgRNA2.4 harboring MS2 hairpin, sgRNA7-1 and sgRNA7-2 harboring two PP7 hairpin variants, and sgRNAB.0 harboring boxB hairpin in the tetraloop and stem loop2 of sgRNA or esgRNA scaffold. The esgRNA scaffold harboring an A-U or C-G flip and extending Cas9-binding hairpin structure. The scRNAs of sgRNA- or esgRNA- represents RNA aptamer hairpins adding at the 3’ end of sgRNA or esgRNA. The sgRNA4.0 bearing two MS2 hairpins at the 3’ end of sgRNA2.0. The f6 aptamer hairpin binds MCP specifically.


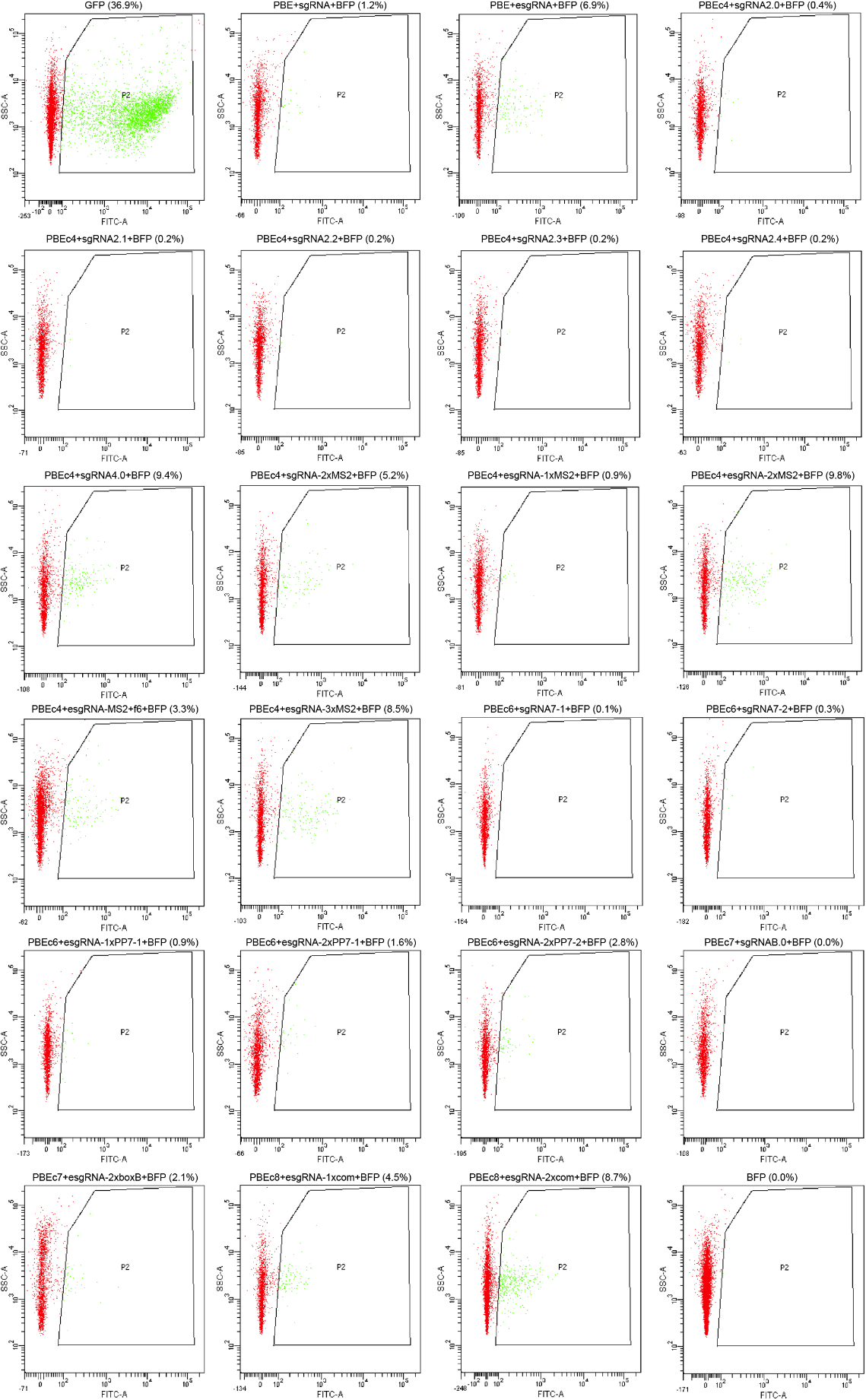


**Figure S3. Flow cytometry of BFP-to-GFP conversion induced by various** **scRNAs and their cognate PBEcs in rice protoplasts.** One of three independent biological replicates is shown.


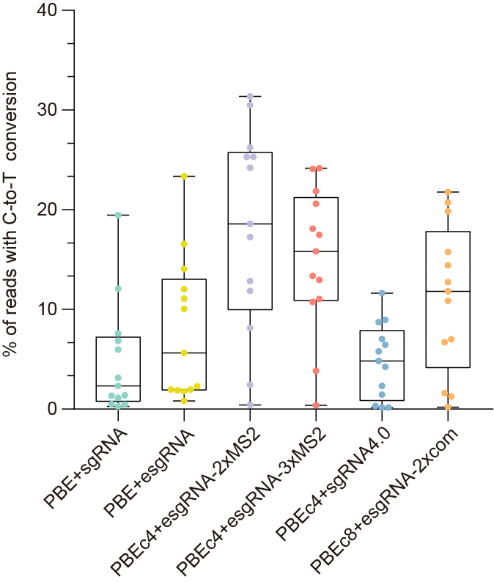


**Figure S4. Frequencies of base editing of endogenous genes by different scRNAs and cognate PBEcs in rice protoplasts.** Data are presented as boxplots (centerline, median; box limits, 25th and 75th percentiles; lower and upper whiskers extend to the lowest or highest value). Data in each boxplot include three independent experiments (*n* = 39).

**
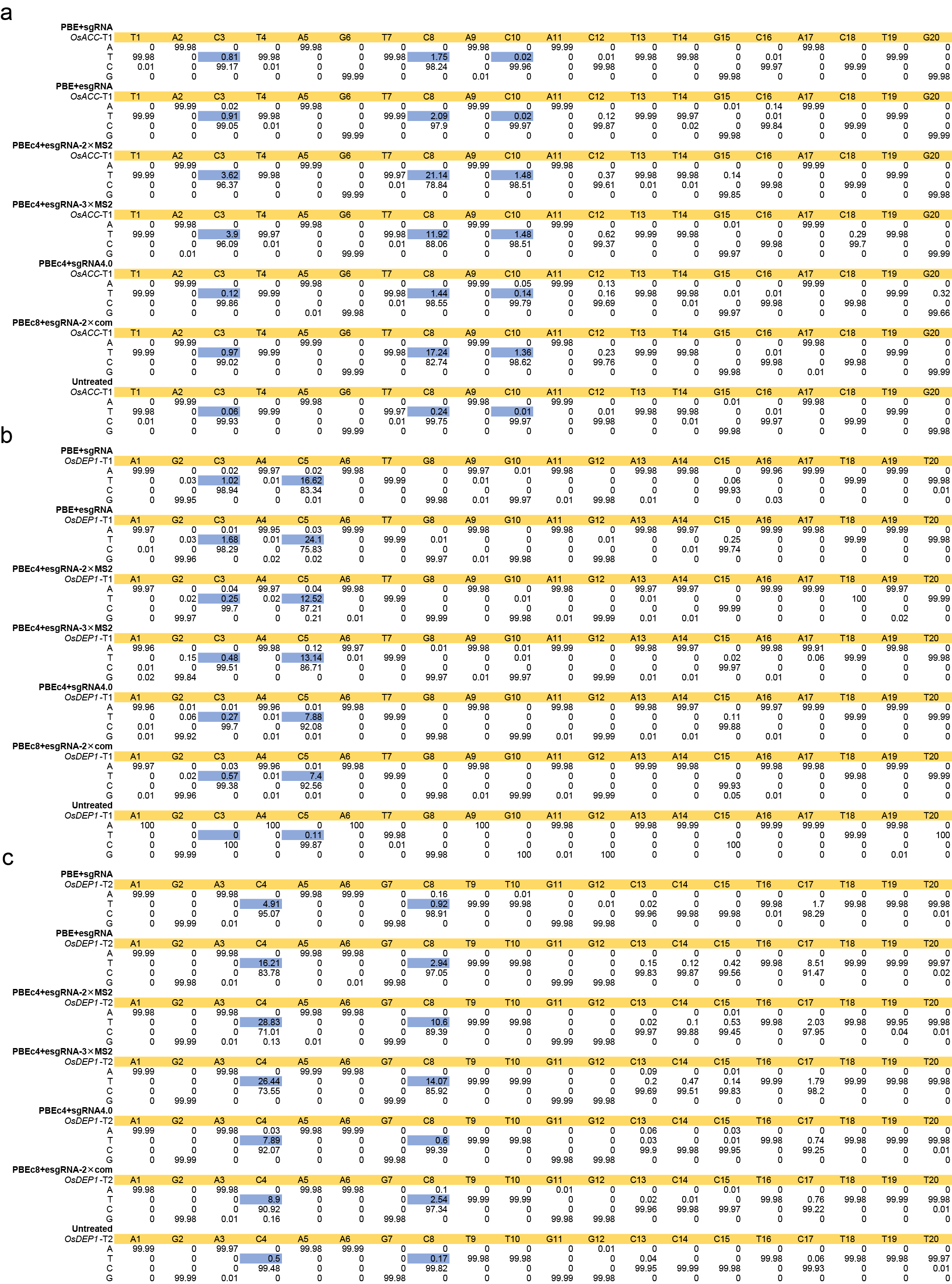
**

**
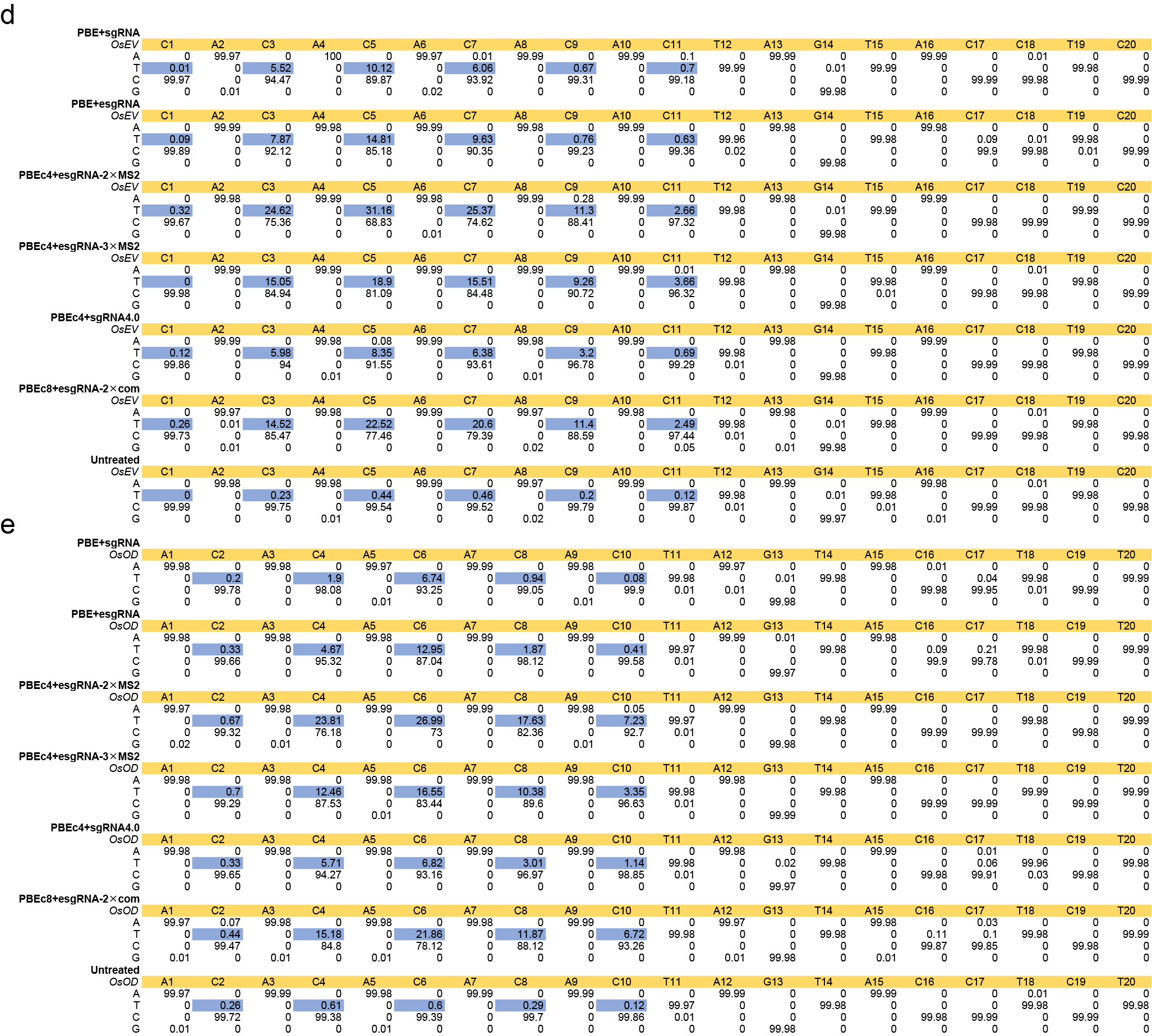
**

**Figure S5. Activities of esgRNA-2×MS2, esgRNA-3×MS2, sgRNA4.0, and esgRNA-2×com with cognate PBEcs in rice protoplasts.** (**a**)-(**e**) are *OsACC*-T1, *OsDEP1*-T1, *OsDEP1*-T2, *OsEV*, and *OsOD* targets, respectively. One of three independent biological replicates is shown.


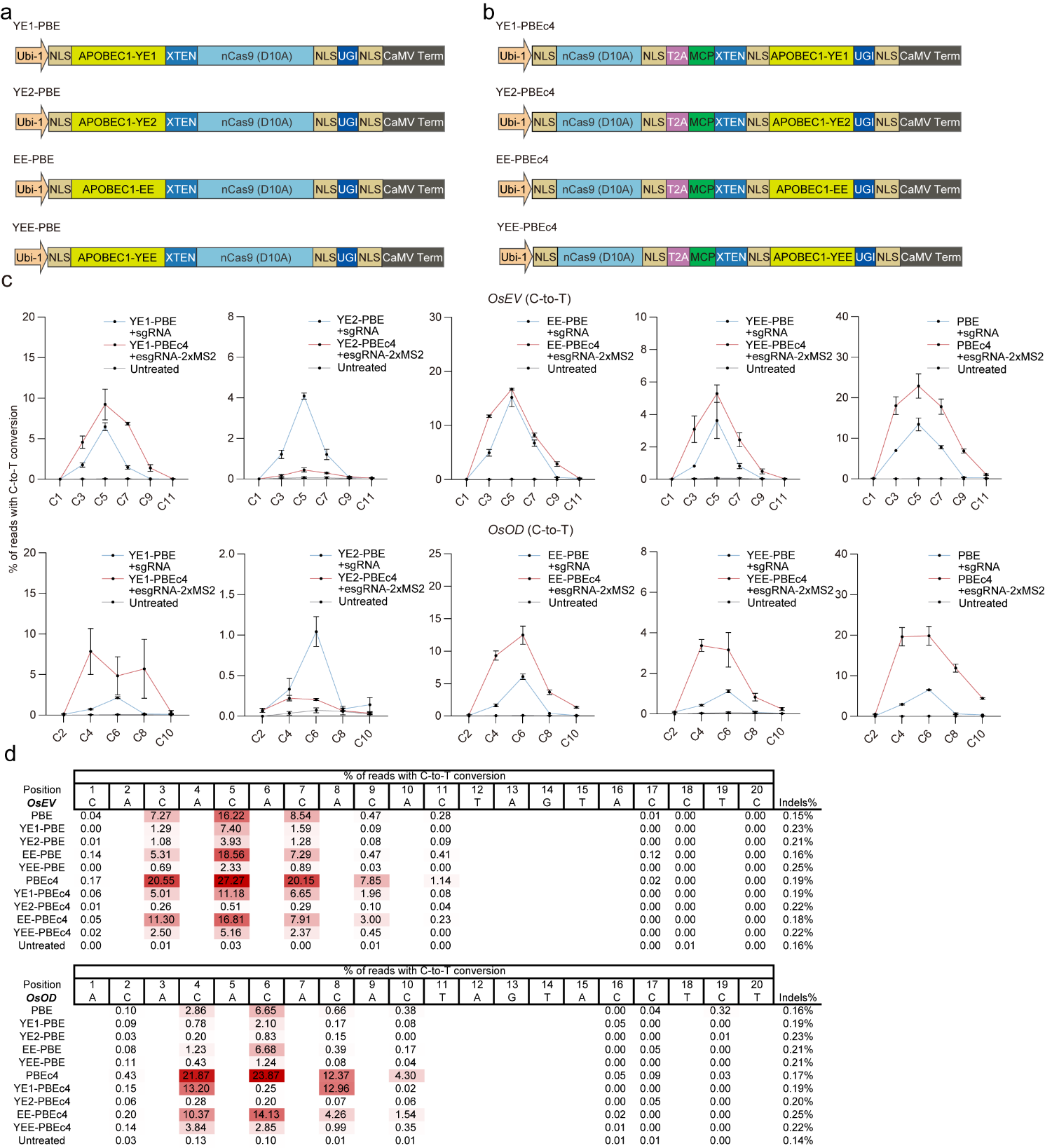


**Figure S6.** **C-to-T editing frequencies generated by scaffold RNA-recruited APOBEC1 narrow-window variants in rice protoplasts.** (**a**) Architectures of YE1-PBE, YE2-PBE, EE-PBE, and YEE-PBE. Abbreviations: XTEN, 16-aa linker; NLS, nuclear localization signal; CaMV, cauliflower mosaic virus; Term, terminator. (**b**) Architectures of YE1-PBEc4, YE2-PBEc4, EE-PBEc4, and YEE-PBEc4. Abbreviations: XTEN, 16-aa linker; NLS, nuclear localization signal; CaMV, cauliflower mosaic virus; Term, terminator. (**c**) C-to-T editing frequencies of APOBEC1 narrow-window variants can be improved by using the scRNA recruitment strategy (*n*=3). An untreated protoplast sample served as control. Values and error bars indicate means ± s.e.m of three independent experiments. (**d**) Activities of APOBEC1 narrow-window variants in nCas9-fused architecture versus scRNA-recruited architecture. *OsEV* and *OsOD* were tested. One of three independent biological replicates is shown.


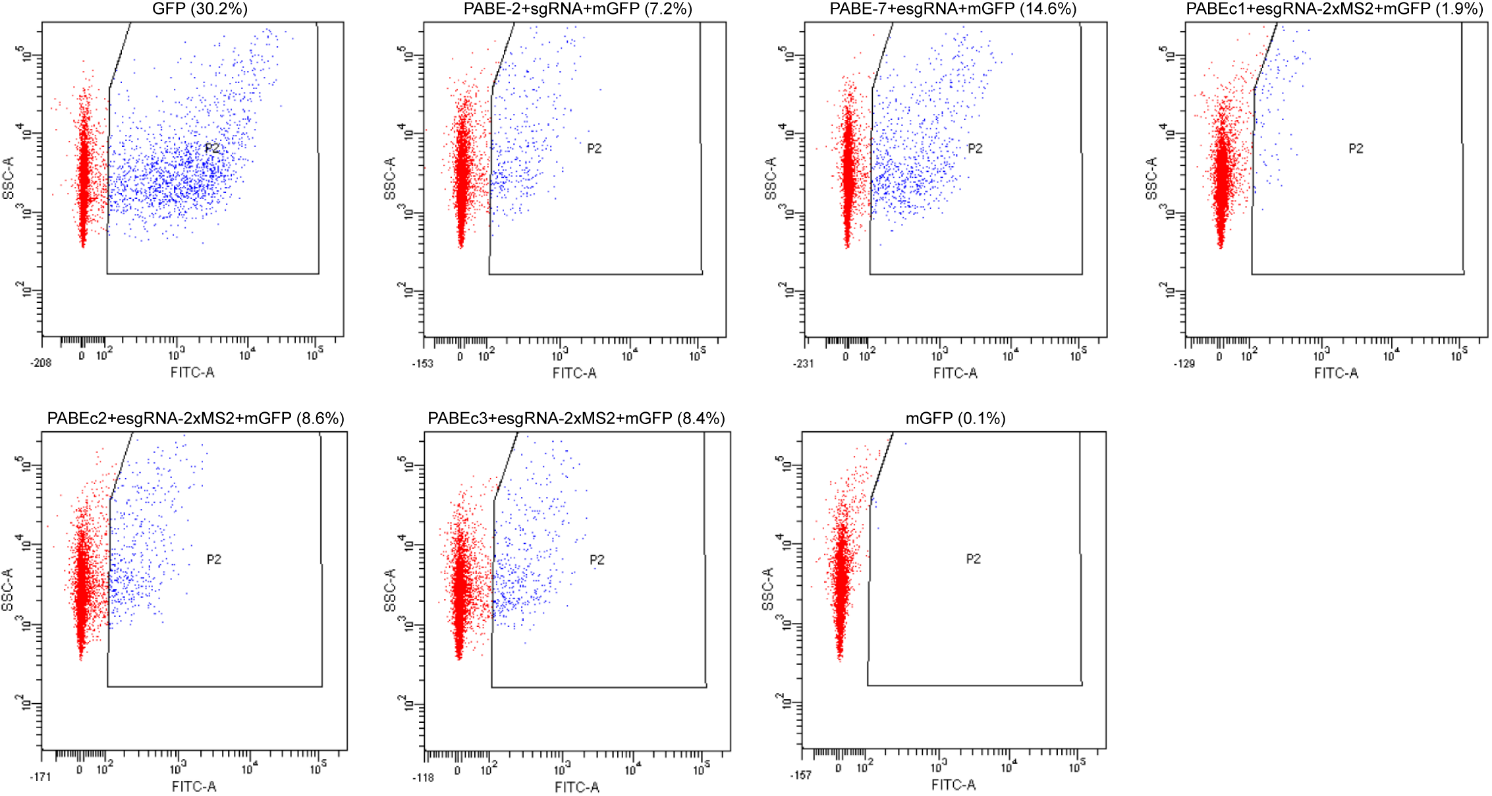


**Figure S7. Flow cytometry of mGFP-to-GFP conversion induced by PABE and the three PABEcs in rice protoplasts.** One of three independent biological replicates is shown.


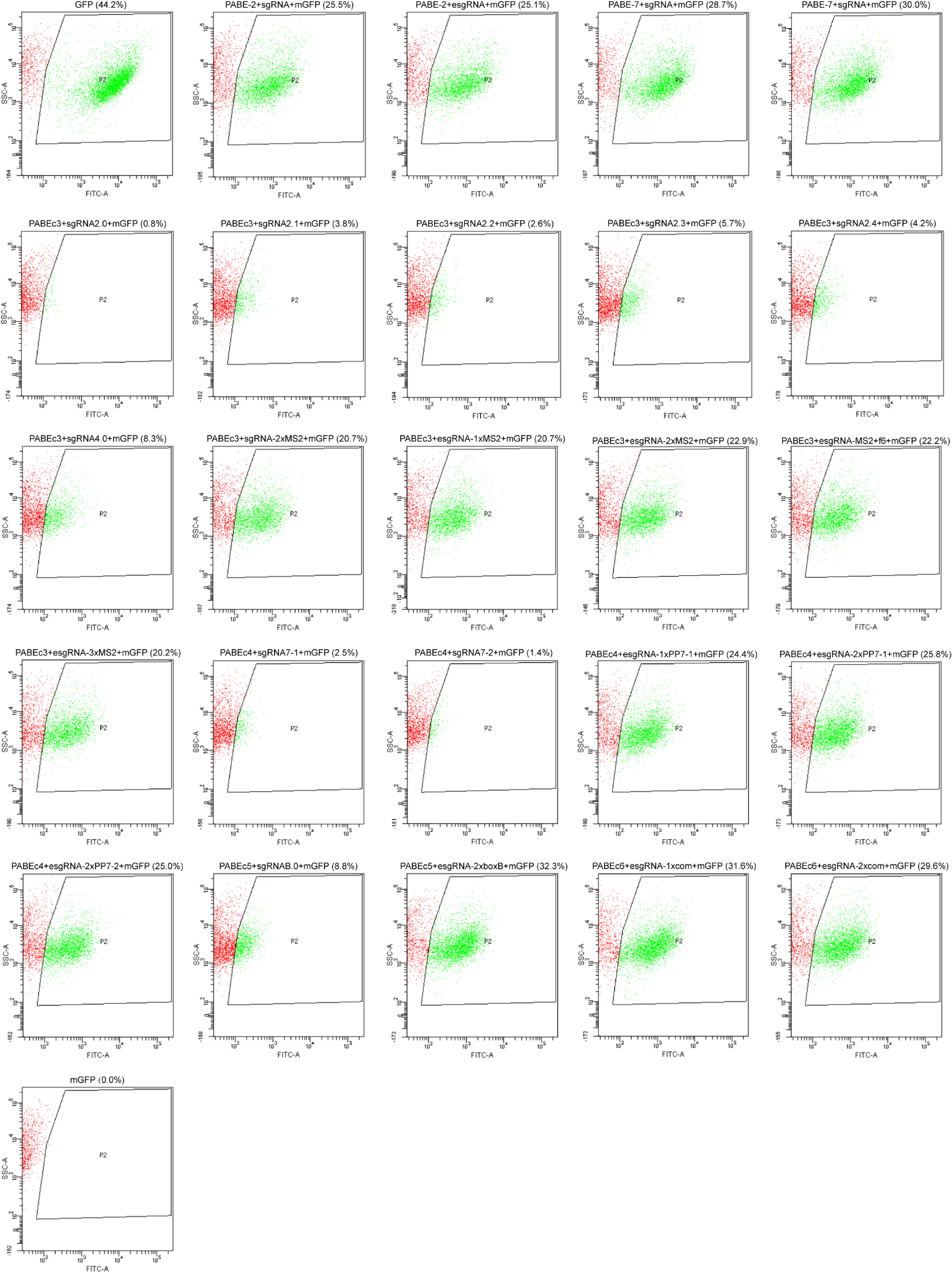


**Figure S8. Flow cytometry of mGFP-to-GFP conversion induced by various scRNAs and their cognate PABEcs in rice protoplasts.** One of three independent biological replicates is shown.


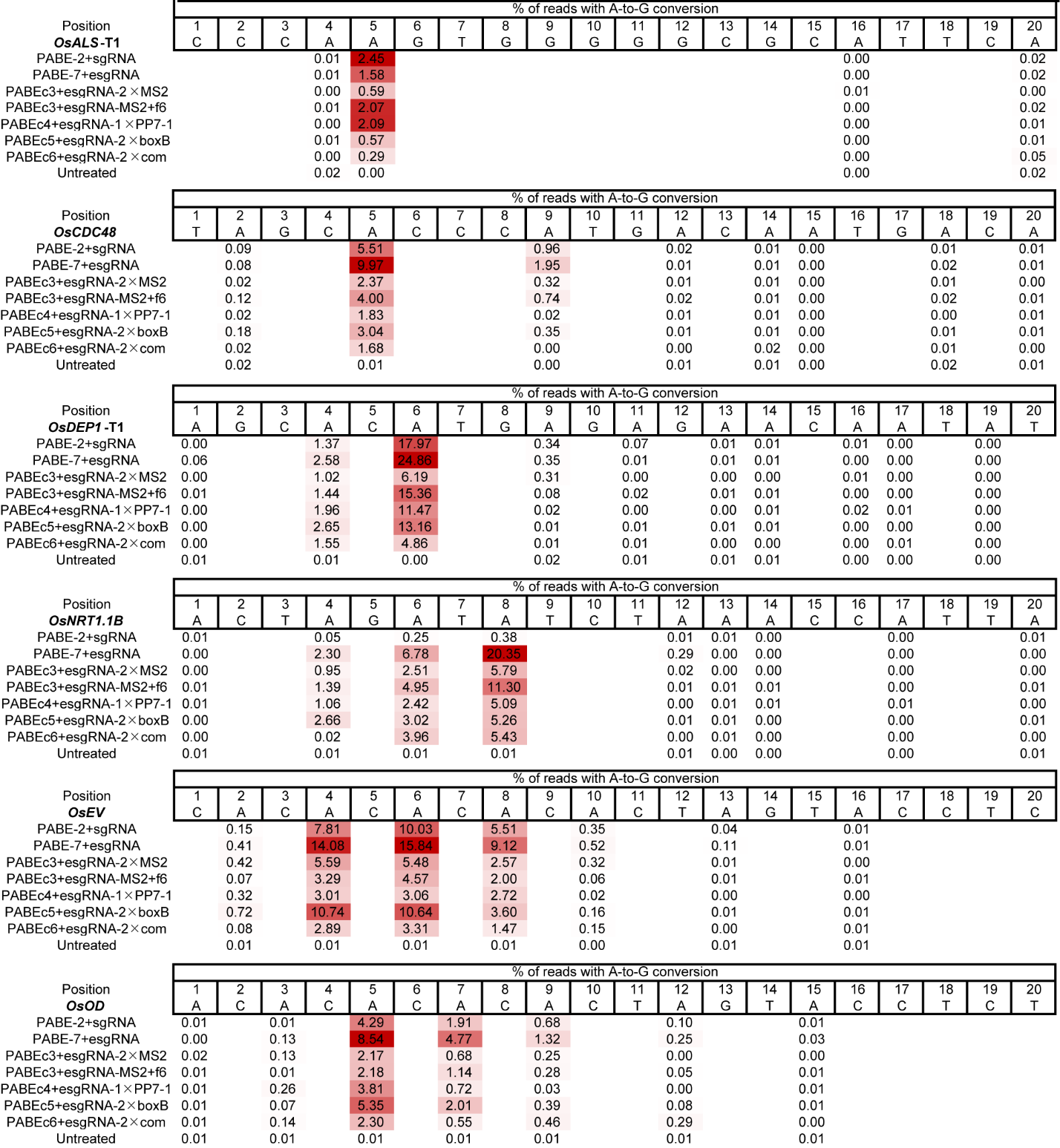


**Figure S9. Activities of the selected scaffold RNAs with their cognate PABEcs in rice protoplasts.** The selected scRNAs of esgRNA-2×MS2, esgRNA-MS2+f6, esgRNA-1×PP7-1, esgRNA-2×boxB, and esgRNA-2×com with cognate PABEcs were tested in rice protoplasts. One of three independent biological replicates is shown.


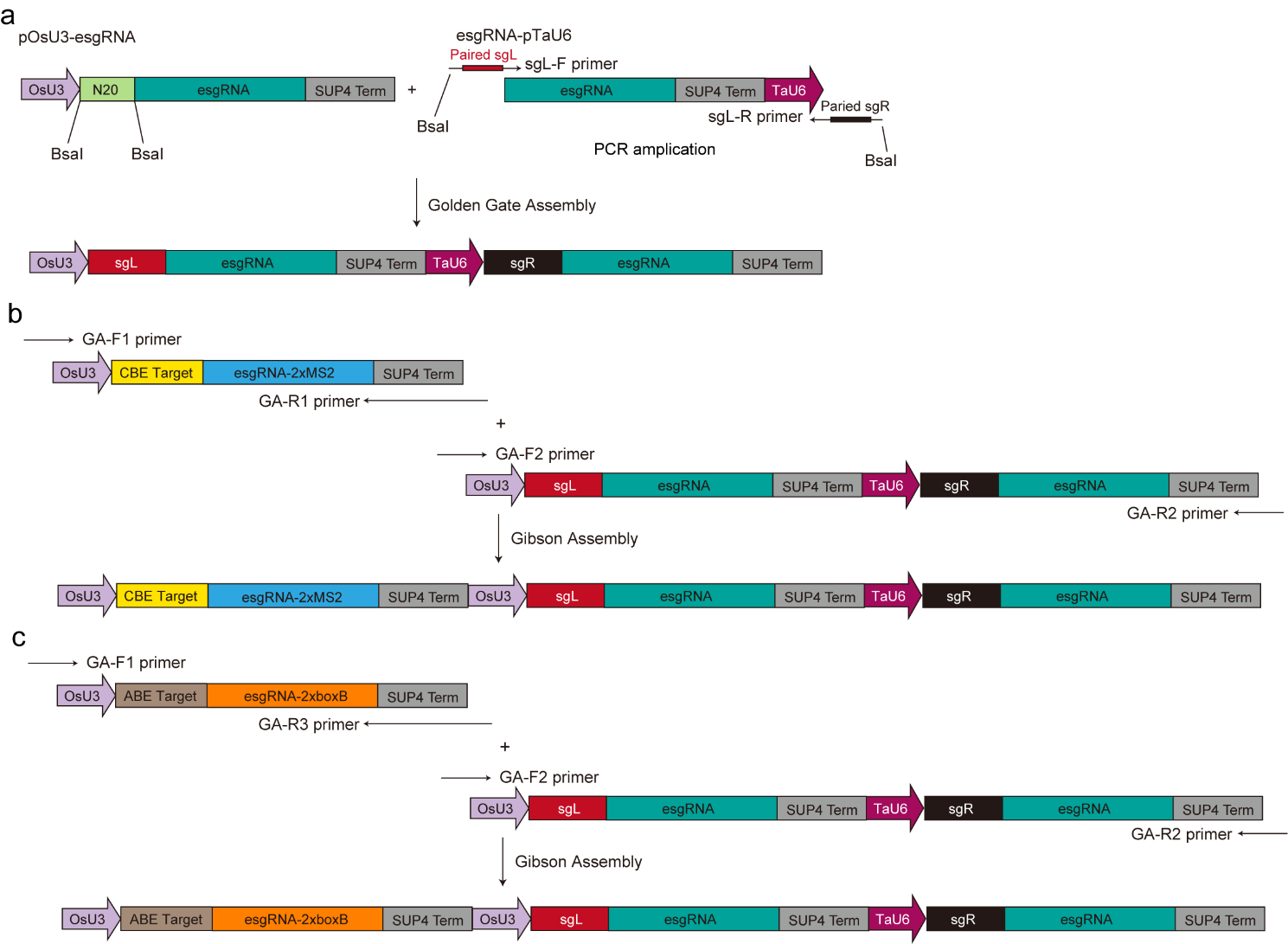


**Figure S10. Schematic of multiple sgRNAs assembly for SWISSv1.1 and SWISSv1.2.** (**a**) Schematic of paired sgRNA assembly. Paired sgRNAs (Paired sgL and Paired sgR) were designed in PAM-out orientation with 40-68 bp between nicking sites. The PCR products were amplified from esgRNA-pTaU6 templates and inserted into the BsaI sites of pOsU3-esgRNA by Golden Gate Assembly. (**b**) Schematic of the CBE target, and paired sgRNAs assembly. The CBE target was inserted into the BsaI sites of pOsU3-esgRNA-2🞨MS2, and the pOsU3-CBE target-esgRNA-2🞨MS2 portion was amplified. PCR products harboring the paired sgRNAs were amplified from the paired sgRNA plasmid. The above two PCR products were assembled into the EcoRI- and HindIII-digested backbone of pOsU3-esgRNA by Multi One Step Cloning. (**c**) Schematic of the ABE target, and paired sgRNAs assembly. The ABE target was inserted into the BsaI sites of pOsU3-esgRNA-2🞨boxB and the pOsU3-ABE target-esgRNA-2🞨MS2 portion was amplified. PCR products harboring paired sgRNAs were amplified from the paired sgRNA plasmid. The above two PCR products were assembled into the EcoRI- and HindIII-digested backbone of pOsU3-esgRNA by Multi One Step Cloning.

**
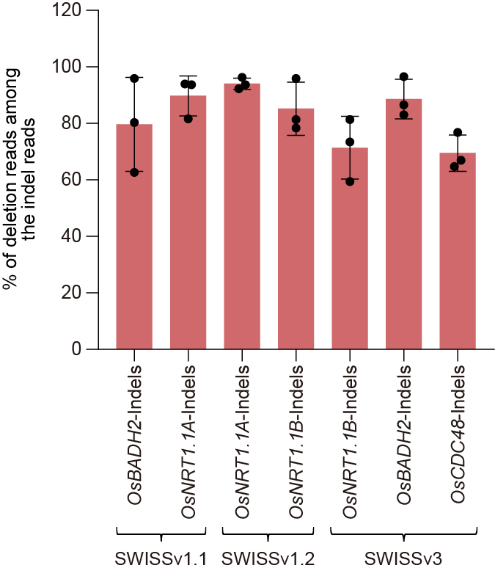
**

**Figure S11. The distributions of deletion reads among the indel sequencing reads for SWISSv1.1, SWISSv1.2, and SWISSv3.** Values and error bars are means ± s.e.m of three independent experiments.


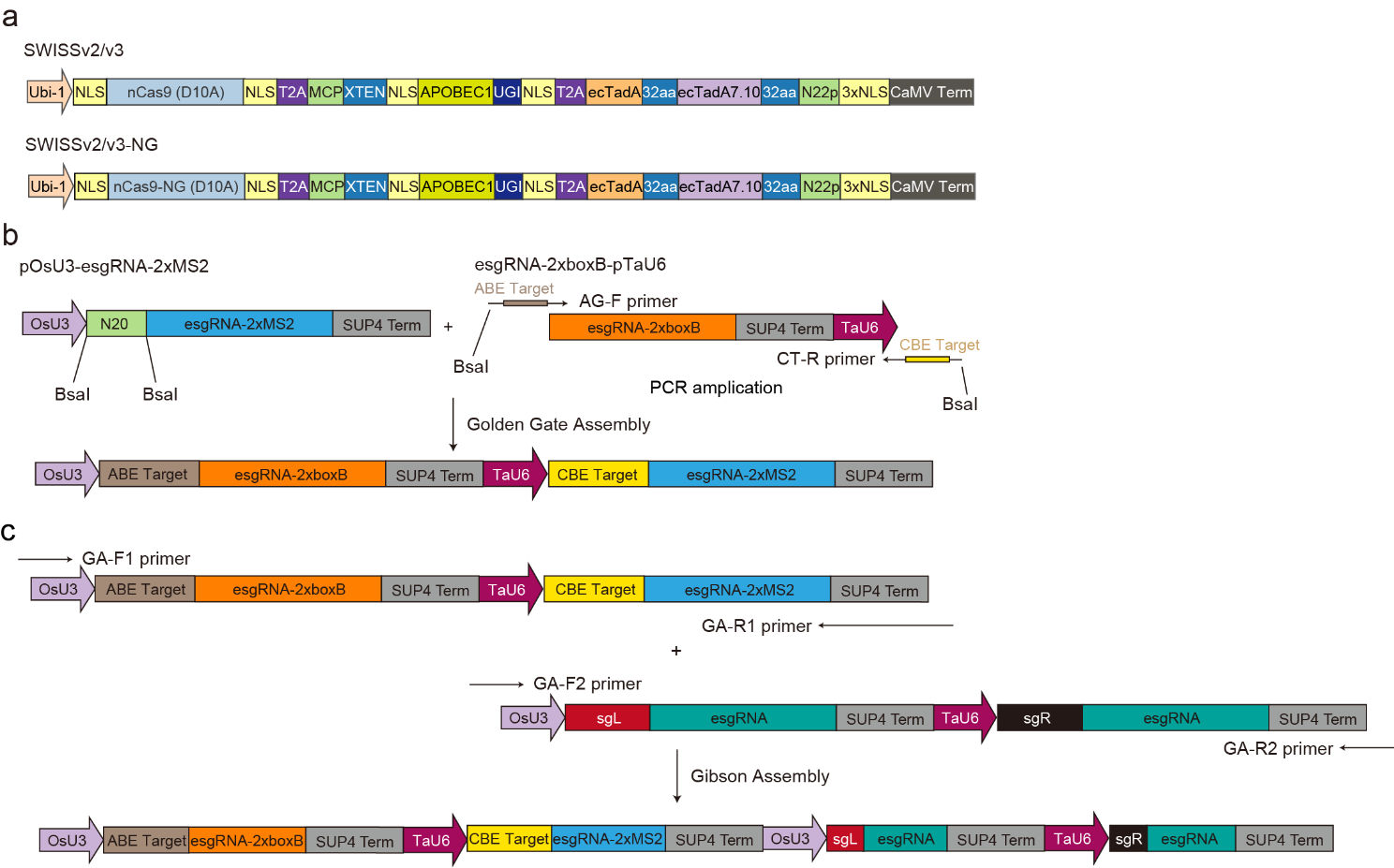


**Figure S12. Schematic of multiple sgRNAs assembly for SWISSv2 and SWISSv3.** (**a**) Architecture of SWISSv2/v3 and SWISSv2/v3-NG. Abbreviations: ecTadA7.10, evolved *Escherichia coli* TadA; aa, amino acid; XTEN, 16 aa linker; NLS, nuclear localization signal; CaMV, cauliflower mosaic virus; Term, terminator. (**b**) Schematic of assembly of the CBE and ABE targets. PCR products were amplified from esgRNA-2🞨boxB-pTaU6 templates and inserted into the BsaI sites of pOsU3-esgRNA-2🞨MS2 by Golden Gate Assembly. (**c**) Schematic of assembly of CBE target, ABE target, and paired sgRNAs. PCR products harboring the CBE and ABE targets were amplified from the CBE target and ABE target dual sgRNA plasmid. PCR products harboring the paired sgRNAs were amplified from the paired sgRNAs plasmidand assembled into the EcoRI- and HindIII-digested backbone of pOsU3-esgRNA by Multi One Step Cloning.


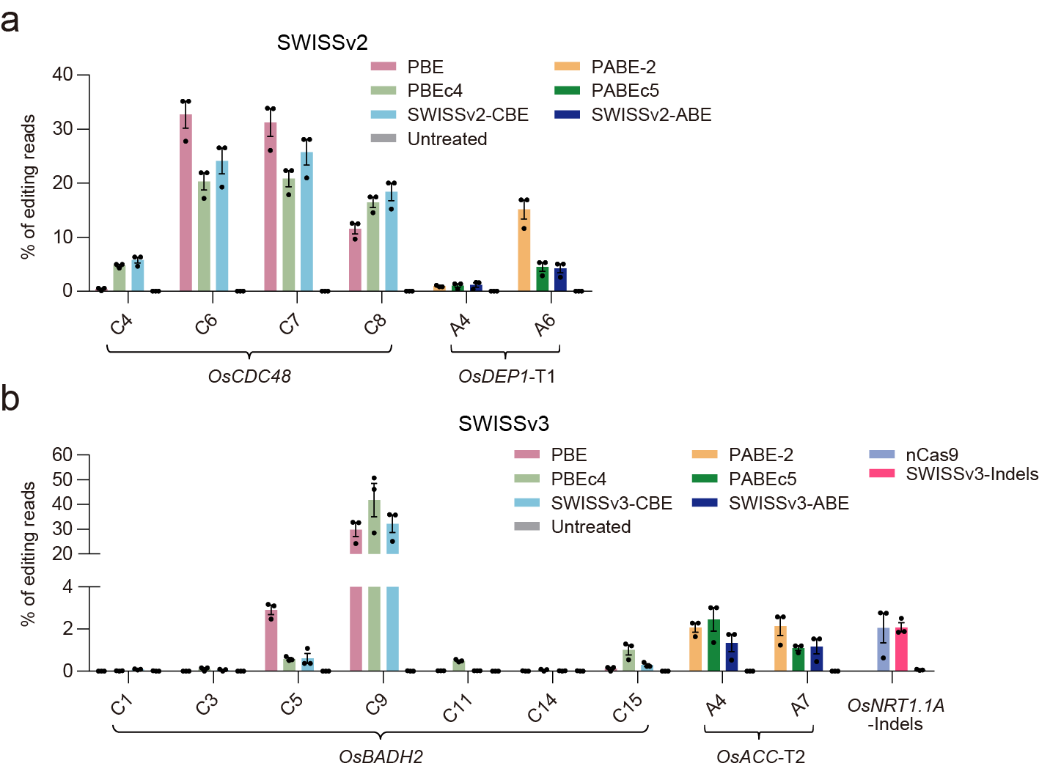


**Figure S13. Comparison of the editing efficiencies between the SWISS systems and the individual genome editing tools (PBE, PBEc4, PABE-2, PABEc5, and paired nCas9).** (**a**) In SWISSv2, the C-to-T and A-to-G editing efficiencies of SWISSv2 were compared with PBE, PBEc4, PABE-2, and PABEc5. Protoplasts samples treated by SWISSv2 and multiple sgRNAs combinations were compared with each combination from PBE, PBEc4, PABE-2, and PABEc5 using respective sgRNA (*n*=3). An untreated protoplast sample served as control. Values and error bars indicate means ± s.e.m of three independent experiments. (**b**) In SWISSv3, the C-to-T, A-to-G, and indel efficiencies of SWISSv3 were compared with PBE, PBEc4, PABE-2, PABEc5, and paired nCas9 (D10A). Protoplasts samples treated by SWISSv3 and multiple sgRNAs combinations were compared with each combination from PBE, PBEc4, PABE-2, PABEc5, and paired nCas9 (D10A) using respective sgRNA (*n*=3). An untreated protoplast sample served as control. Values and error bars indicate means ± s.e.m of three independent experiments.


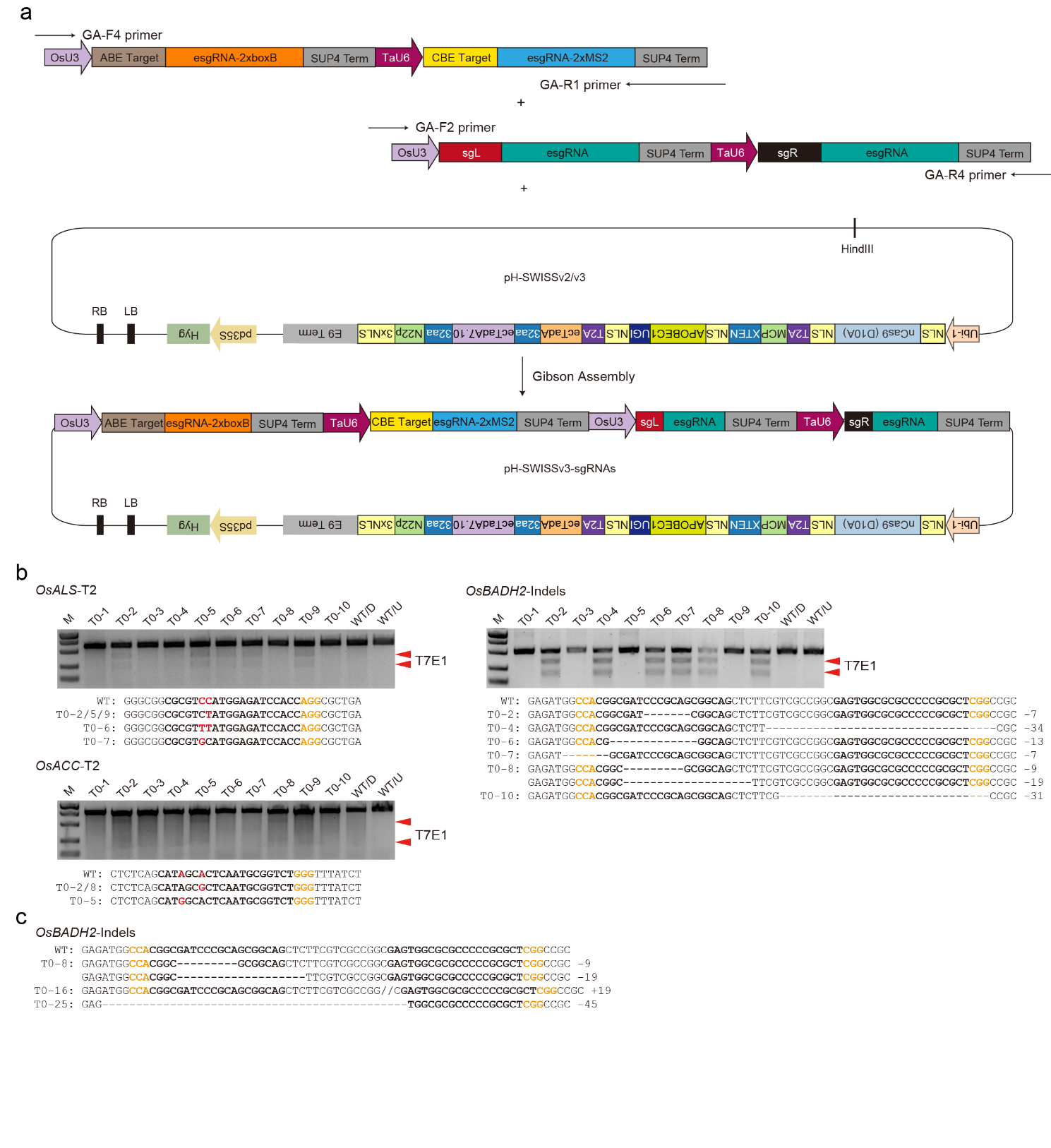


**Figure S14. Simultaneous CBE, ABE, and DSB formation in rice plants.** (**a**) Schematic of assembly of the CBE target, ABE target, and paired sgRNAs to produce the binary vector. PCR products harboring the CBE target and ABE target were amplified from the CBE target and ABE target dual sgRNAs plasmid. PCR products harboring the paired sgRNAs were amplified from the paired sgRNA plasmid. The above two PCR products were assembled into HindIII-digested pH-SWISSv2/v3 binary vector by Multi One Step Cloning. (**b**) Outcome of T7E1 assays for *OsALS*-T2, *OsACC*-T2 and *OsBADH2-*indel mutants. The C/A bases targeted are highlighted in red. The PAM sequence is shown in brown. Ten T0 plantlets (T0-1 to T0-10) were analyzed. WT/D and WT/U indicate the genomic DNA amplicons of the wild type (WT) control with or without digestion by T7E1. In total fifty-five regenerated plants were identified. The bands marked by red arrowheads are diagnostic of positive genome editing. Sequences were determined by Sanger sequencing, and the sequencing chromatograms of the indels were further analyzed with the online tools DSDecodeM [50] and TIDE [51]. (**c**) Three representative mutants of *OsBADH2*-Indel were confirmed by Sanger Sequencing. Sequence alignment results indicated that both paired sgRNAs cleaved in rice genome.

**Table S1.** **The sgRNA sequences used to compare the activities of PBEcs and PABEcs.**

| Target name | Target sequence (5′-3′) ^a^ | Oligo (5′-3′) |
| --- | --- | --- |
| *OsACC*-T1 | TACTAGTCACACTTGCACTG**TGG** | F: ggcgTACTAGTCACACTTGCACTG |
|  |  | R: aaacCAGTGCAAGTGTGACTAGTA |
| *OsALS*-T1 | CCCAAGTGGGGGCGCATTCA**AGG** | F: ggcgCCCAAGTGGGGGCGCATTCA |
|  |  | R: aaacTGAATGCGCCCCCACTTGGG |
| *OsCDC48* | TAGCACCCATGACAATGACA**TGG** | F: ggcgTAGCACCCATGACAATGACA |
|  |  | R: aaacTGTCATTGTCATGGGTGCTA |
| *OsDEP1*-T1 | AGCACATGAGAGAACAATAT**TGG** | F: ggcgAGCACATGAGAGAACAATAT |
|  |  | R: aaacATATTGTTCTCTCATGTGCT |
| *OsDEP1*-T2 | AGACAAGCTTGGCCCTCTTT**GGG** | F: ggcgAGACAAGCTTGGCCCTCTTT |
|  |  | R: aaacAAAGAGGGCCAAGCTTGTCT |
| *OsNRT1.1B* | ACTAGATATCTAAACCATTA**AGG** | F: ggcgACTAGATATCTAAACCATTA |
|  |  | R: aaacTAATGGTTTAGATATCTAGT |
| *OsEV* | CACACACACACTAGTACCTC**TGG** | F: ggcgCACACACACACTAGTACCTC |
|  |  | R: aaacGAGGTACTAGTGTGTGTGTG |
| *OsOD* | ACACACACACTAGTACCTCT**GGG** | F: ggcgACACACACACTAGTACCTCT |
|  |  | R: aaacAGAGGTACTAGTGTGTGTGT |

^a^ PAM motifs are written in bold and underlined.

**Table S2. The sgRNA sequences used for SWISSv1.1, SWISSv1.2, SWISSv2, and SWISSv3 editing in rice protoplasts.**

| Type of genome editing | Target name | Target sequence (5′-3′) ^a^ |
| --- | --- | --- |
| CBE | *OsALS*-T2 | CGCGTCCATGGAGATCCACC**AGG** |
|  | *OsBADH2* | CGCGCAATCGCGGCCAAGGT**AGG** |
|  | *OsCDC48* | TAGCACCCATGACAATGACA**TGG** |
|  | *OsGL2* | CTTCCACAGGCTTTCTTGAA**CGG** |
|  | *OsDEP1*-NGA | GTGAAGCTCATCCTACAAAA**GGA** |
|  | *OsCDC48*-NGA | CCATGACAATGACATGGGAA**CGA** |
| ABE | *OsACC*-T2 | CATAGCACTCAATGCGGTCT**GGG** |
|  | *OsDEP1*-T1 | AGCACATGAGAGAACAATAT**TGG** |
|  | *OsNRT1.1A* | GTGGACGCTGGCGACGCTGA**CGG** |
|  | *OsAAT*-NGT | TACAACAAGGATCCCAGCCC**CGT** |
|  | *OsDEP1*-NGT | GCACATGAGAGAACAATATT**GGT** |
| DSB ^b^ | *OsBADH2*-Indels-sgL | CTGCCGCTGCGGGATCGCCG**TGG** |
|  | *OsBADH2*-Indels-sgR | GAGTGGCGCGCCCCCGCGCT**CGG** |
|  | *OsNRT1.1A*-Indels-sgL | TCGTCGGAGGCGAGTGGTGG**TGG** |
|  | *OsNRT1.1A*-Indels-sgR | CTCTCACCTGCGTTTGTCGC**CGG** |
|  | *OsNRT1.1B*-Indels-sgL | GTTTAGATATCTAGTAGTGC**TGG** |
|  | *OsNRT1.1B*-Indels-sgR | GGTCAAATCAAGTTATTTTT**AGG** |
|  | *OsCDC48*-Indels-sgL | AATGACATGGGAACGAGCTT**TGA** |
|  | *OsCDC48*-Indels-sgR | GTGCTACAAACCGGCCAAAC**AGT** |

^a^ The PAM motis are written in bold and underlined.

^b^ Paired sgRNAs were designed according to the rules: (1) PAM in out orientation; (2) distance between nicking sites 40-68 bp.

**Table S3.** **Potential off-target sites analyzed for *OsALS*-T2, *OsACC*-T2, *OsBADH2*-Indels-sgL, and *OsBADH2*-Indels-sgR triple mutants.**

| Target name | Potential off-target site | Sequence (5′-3′) ^a^ | No. of mismatches | Target locus | Detection method | Edited |
| --- | --- | --- | --- | --- | --- | --- |
| *OsALS*-T2 | On-target | CGCGTCCATGGAGATCCACC**AGG** | 0 | LOC_Os02g30630.1 | T7E1 and Sanger sequencing | C-to-T |
|  | Off-target1 | CGCGTCaATGGAGATCCACC**AGG** | 1 | LOC_Os04g32010.1 | T7E1 and Sanger sequencing | NO |
|  | Off-target2 | CGCGTCgATGGAGATCCACC**AGG** | 1 | LOC_Os04g31960.1 | T7E1 and Sanger sequencing | NO |
|  | Off-target3 | CGCGTCCAaGGAGtTCCAgC**AGG** | 3 | LOC_Os10g08540.1 | T7E1 and Sanger sequencing | NO |
|  | Off-target4 | CGCGTCCAaGGAGtTCCAgC**AGG** | 3 | LOC_Os10g08540.1 | T7E1 and Sanger sequencing | NO |
| *OsACC*-T2 | On-target | CATAGCACTCAATGCGGTCT**GGG** | 0 | LOC_Os05g22940.1 | T7E1 and Sanger sequencing | A-to-G |
|  | Off-target | N.A. ^b^ | 1 - 3 | N.A. ^b^ | N.A. ^b^ | N.A. ^b^ |
| *OsBADH2*-Indels-sgL | On-target | CTGCCGCTGCGGGATCGCCG**TGG** | 0 | LOC_Os08g32870.1 | T7E1 and Sanger sequencing | Indels |
|  | Off-target1 | CTGCCGCTGCGGcgTCGgCG**AGG** | 3 | LOC_Os03g52640.1 | T7E1 and Sanger sequencing | NO |
|  | Off-target2 | CTGCCGCTcCtGGAaCGCCG**AGG** | 3 | LOC_Os01g07160.1 | T7E1 and Sanger sequencing | NO |
|  | Off-target3 | CgGCCGCaGCGGGAgCGCCG**GGG** | 3 | LOC_Os02g35190.1 | T7E1 and Sanger sequencing | NO |
|  | Off-target4 | CTGCCGCTGCtGccTCGCCG**TGG** | 3 | LOC_Os02g43220.1 | T7E1 and Sanger sequencing | NO |
|  | Off-target5 | CcGCCGCgGCGGGtTCGCCG**CGG** | 3 | LOC_Os10g37770.1 | T7E1 and Sanger sequencing | NO |
|  | Off-target6 | CTGCtGCTGCGccATCGCCG**GGG** | 3 | LOC_Os06g46440.1 | T7E1 and Sanger sequencing | NO |
|  | Off-target7 | CTGgCGCTGCGGGAgCGgCG**TGG** | 3 | LOC_Os11g03550.1 | T7E1 and Sanger sequencing | NO |
|  | Off-target8 | CTGCaGCcGCGGGAaCGCCG**CGG** | 3 | LOC_Os11g42240.1 | T7E1 and Sanger sequencing | NO |
| *OsBADH2*-Indels-sgR | On-target | GAGTGGCGCGCCCCCGCGCT**CGG** | 0 | LOC_Os08g32870.1 | T7E1 and Sanger sequencing | Indels |
|  | Off-target1 | GAGTGGCGCGCCCgCGCGgT**TGG** | 2 | LOC_Os04g18380.1 | T7E1 and Sanger sequencing | NO |
|  | Off-target2 | GAGTGGCGCGCCtCCaCGCT**CGG** | 2 | LOC_Os01g22370.1 | T7E1 and Sanger sequencing | NO |
|  | Off-target3 | GAGTGGCGCGCCCgCGCGgT**GGG** | 2 | LOC_Os01g64256.1 | T7E1 and Sanger sequencing | NO |
|  | Off-target4 | GAGTGGCGCttCCCCGCGCa**CGG** | 2 | LOC_Os06g12810.1 | T7E1 and Sanger sequencing | NO |

^a^ PAM motifs are written in bold and underlined.

^b^ N.A. = not applicable.

**Table S4.** **Statistics of whole genome sequencing analysis.**

| Sample name | Total base (Mb) | Mapping ratio (%) | Properly paired Mapping ratio (%) | Insert size average (bp) | Coverage (>Q30) (%) | Average depth (Q30) |
| --- | --- | --- | --- | --- | --- | --- |
| PBE-#1 | 31523.91 | 99.54 | 98.07 | 360 | 98.20 | 70.00 |
| PBE-#2 | 17976.66 | 99.72 | 98.38 | 326 | 94.41 | 38.77 |
| PBE-#3 | 20383.44 | 99.66 | 98.53 | 353 | 95.65 | 42.17 |
| PBE-#4 | 22339.62 | 99.60 | 97.99 | 371 | 96.94 | 44.16 |
| PBE-#5 | 27714.94 | 99.63 | 98.40 | 370 | 97.34 | 51.03 |
| PBE-#6 | 22368.40 | 99.58 | 98.32 | 338 | 93.65 | 42.04 |
| SWISSv2/v3-#1 | 24132.38 | 99.67 | 97.30 | 358 | 96.74 | 50.51 |
| SWISSv2/v3-#2 | 23075.29 | 99.68 | 98.40 | 371 | 95.55 | 49.07 |
| SWISSv2/v3-#3 | 23420.27 | 99.72 | 98.12 | 352 | 97.10 | 48.94 |
| SWISSv2/v3-#4 | 28843.80 | 99.76 | 98.60 | 329 | 97.94 | 58.86 |
| SWISSv2/v3-#5 | 23281.88 | 99.71 | 97.41 | 352 | 96.97 | 48.57 |
| SWISSv2/v3-#6 | 20155.09 | 99.69 | 98.62 | 371 | 97.33 | 44.91 |

**Table S5. Primer sequences used in this study.**

| Primer names | Primer sequences (5'-3') | Applications |
| --- | --- | --- |
| OsACC-T1-1F | AGCTTCTTGATCTTGTTCCAGTTTCAG | Sanger sequencing and 1st round PCR for deep sequencing |
| OsACC-T1-1R | CCTCACTGCAGTTTCAAATGCCTACA |  |
| OsCDC48-1F | TCTTTCTGATTAATGGCCCGGAG |  |
| OsCDC48-1R | GGATGCATTGGAGAGCAGCC |  |
| OsDEP1-T1-1F | AACATGAAAGAATCCAGCTCCATTC |  |
| OsDEP1-T1-1R | CCTAAGTGTGACATACAAGTGCAAC |  |
| OsDEP1-T2-1F | GCAAGTAGGATGCTGTGAAG |  |
| OsDEP1-T2-1R | GAAGTTCTCTGATATCTGAAGAGCTTCTG |  |
| OsALS-T1-1F | CATACTTGGGCAACCCGGAATG |  |
| OsALS-T1-1R | AGGACAAGAAACTTACATGATATCTTGTG |  |
| OsNRT1.1B-1F | AGCTAGGAGTAGAGAACGAGACATATAC |  |
| OsNRT1.1B-1R | GTTGGGAGAATAGCTGAAGCTATCGG |  |
| OsOD-1F | GGGAGATGAGAGAGCTTGTGCC |  |
| OsOD-1R | GAGTAGTGTAGTACTGAAGAAGCACAGC |  |
| OsEV-1F | GGGAGATGAGAGAGCTTGTGCC |  |
| OsEV-1R | GAGTAGTGTAGTACTGAAGAAGCACAGC |  |
| OsALS-T2-1F | GCGACACCACGTCCTTCCCG |  |
| OsALS-T2-1R | CCCGAGGACGCGAGGAAGAAG |  |
| OsBADH2-1F | GTCCCCCATCGGTACCCTCC |  |
| OsBADH2-1R | CACCCTCTCCGCTTGAACCC |  |
| OsGL2-1F | TTGGATACGGTTCCTACTTCGGC |  |
| OsGL2-1R | CTGAAATCTTTGTTTCCGGCACCAG |  |
| OsDEP1-NGA-1F | AACATGAAAGAATCCAGCTCCATTC |  |
| OsDEP1-NGA-1R | CCTAAGTGTGACATACAAGTGCAAC |  |
| OsCDC48-NGA-1F | TCTTTCTGATTAATGGCCCGGAG |  |
| OsCDC48-NGA-1R | GGATGCATTGGAGAGCAGCC |  |
| OsACC-T2-1F | TATCCGTGGTGTTGATGACAGCC |  |
| OsACC-T2-1R | CGCAATCTGAGTATATAGAGGCATC |  |
| OsNRT1.1A-1F | ATACTGATGTACACACACATGATTGAAAC |  |
| OsNRT1.1A-1R | ATGCGCTGGAGCGGGGTGAG |  |
| OsAAT-NGT-1F | AGGTTAAGTACGCTGGTGCGC |  |
| OsAAT-NGT-1R | GACGATTCAAAGCAAGAATGGTGCC |  |
| OsDEP1-NGT-1F | AACATGAAAGAATCCAGCTCCATTC |  |
| OsDEP1-NGT-1R | CCTAAGTGTGACATACAAGTGCAAC |  |
| OsBADH2-Indels-1F | CAAGGCAGCACAGAACAGAGC |  |
| OsBADH2-Indels-1R | CGATTGCGCGGAGGTACTTGG |  |
| OsNRT1.1A-Indels-1F | TAATCTATCTATCTATCTTCCCCTTGTTGATACA |  |
| OsNRT1.1A-Indels-1R | GAAGTTGGTGACGACGTTGGC |  |
| OsNRT1.1B-Indels-1F | AGCTAGGAGTAGAGAACGAGACATATAC |  |
| OsNRT1.1B-Indels-1R | GTTGGGAGAATAGCTGAAGCTATCGG |  |
| OsCDC48-Indels-1F | TCTTTCTGATTAATGGCCCGGAG |  |
| OsCDC48-Indels-1R | GGATGCATTGGAGAGCAGCC |  |
| OsACC-T1-2F | TGAAACTTAAGTTGGACTGCG | 2nd round PCR for deep sequencing without barcode |
| OsACC-T1-2R | CTGGGTATGAGGACCAGCC |  |
| OsCDC48-2F | AAGTTGAGAGGCGCATCG |  |
| OsCDC48-2R | CATCAGGAACACCAATGTCAATC |  |
| OsDEP1-T1-2F | ACAGGTAGAAAAACTTTTGTGGG |  |
| OsDEP1-T1-2R | CATGAAGGGCAGTAGTACATACTC |  |
| OsDEP1-T2-2F | CTTGTAGTACTGTTTCTAGGCGG |  |
| OsDEP1-T2-2R | AGGTCAGTTGTCCATGCCCA |  |
| OsALS-T1-2F | CCGCCATCAAGAAGATGCTC |  |
| OsALS-T1-2R | CTGGTGCTTTGCCAACATAC |  |
| OsNRT1.1B-2F | ACTCCAGCCACTCACTGTC |  |
| OsNRT1.1B-2R | GTCGTTGGGACTGGGCTAC |  |
| OsOD-2F | CCTTGCTTTCATTCTTCAGTGC |  |
| OsOD-2R | GCAGACCAAGATCCCAAGAAC |  |
| OsEV-2F | CCTTGCTTTCATTCTTCAGTGC |  |
| OsEV-2R | GCAGACCAAGATCCCAAGAAC |  |
| OsALS-T2-2F | ATCCTCGTGGAGGCGCTGG |  |
| OsALS-T2-2R | GTACCCGGACGCCGCGAAC |  |
| OsBADH2-2F | GGAGGCGCTGAAGAGGAACC |  |
| OsBADH2-2R | GAGCTCCTCCCCCTGTACGG |  |
| OsGL2-2F | TACGGACGGCAAGAAGTGGC |  |
| OsGL2-2R | TGGAAGGCGGTGGAGGTGAC |  |
| OsDEP1-NGA-2F | ACAGGTAGAAAAACTTTTGTGGG |  |
| OsDEP1-NGA-2R | CATGAAGGGCAGTAGTACATACTC |  |
| OsCDC48-NGA-2F | AAGTTGAGAGGCGCATCG |  |
| OsCDC48-NGA-2R | CATCAGGAACACCAATGTCAATC |  |
| OsACC-T2-2F | TTCCCATGGCTGCAGAGC |  |
| OsACC-T2-2R | ATCCTGGAGTTCCTCTGACC |  |
| OsNRT1.1A-2F | CTGCAAGTAAGCTTAACAAAAGTTGC |  |
| OsNRT1.1A-2R | GGGCGTACACCGTCCAGAAC |  |
| OsAAT-NGT-2F | TCGACCTGATCGGTGCTC |  |
| OsAAT-NGT-2R | ATCCACCACCAATCCAATCC |  |
| OsDEP1-NGT-2F | ACAGGTAGAAAAACTTTTGTGGG |  |
| OsDEP1-NGT-2R | CATGAAGGGCAGTAGTACATACTC |  |
| OsBADH2-Indels-2F | CCGACCCCAAATCGCACAG |  |
| OsBADH2-Indels-2R | GGGTACCGATGGGGGACTCG |  |
| OsNRT1.1A-Indels-2F | CCTGGGGGTAGCATAGTAGATTC |  |
| OsNRT1.1A-Indels-2R | TCTCCGGCAACATCCCCACC |  |
| OsNRT1.1B-Indels-2F | ACTCCAGCCACTCACTGTC |  |
| OsNRT1.1B-Indels-2R | GTCGTTGGGACTGGGCTAC |  |
| OsCDC48-Indels-2F | AAGTTGAGAGGCGCATCG |  |
| OsCDC48-Indels-2R | CATCAGGAACACCAATGTCAATC |  |
| Barcode-2AF | CGATGT | Barcode sequences for deep sequencing |
| Barcode-2AR | TGACCA |  |
| Barcode-2BF | ACAGTG |  |
| Barcode-2BR | GCCAAT |  |
| Barcode-2CF | CAGATC |  |
| Barcode-2CR | CTTGTA |  |
| Barcode-2DF | AGTCAA |  |
| Barcode-2DR | AGTTCC |  |
| Barcode-2EF | GTAGAG |  |
| Barcode-2ER | GTCCGC |  |
| Barcode-2FF | GTTTCG |  |
| Barcode-2FR | CGTACG |  |
| Barcode-2GF | ACTGAT |  |
| Barcode-2GR | ATGAGC |  |
| Barcode-2HF | CAACTA |  |
| Barcode-2HR | CACCGG |  |
| Barcode-2IF | CAGGCG |  |
| Barcode-2IR | CATGGC |  |
| Barcode-2JF | CGGAAT |  |
| Barcode-2JR | CTAGCT |  |
| Barcode-2KF | GCGCTA |  |
| Barcode-2KR | TAATCG |  |
| Barcode-2LF | TACAGC |  |
| Barcode-2LR | TATAAT |  |
| Barcode-2MF | TCATTC |  |
| Barcode-2MR | TCCCGA |  |
| Barcode-2NF | TCGAAG |  |
| Barcode-2NR | TCGGCA |  |
| Barcode-2OF | ATGTCA |  |
| Barcode-2OR | CCGTCC |  |
| Barcode-2PF | ATCACG |  |
| Barcode-2PR | TTAGGC |  |
| Barcode-2QF | ACTTGA |  |
| Barcode-2QR | GATCAG |  |
| Barcode-2RF | TAGCTT |  |
| Barcode-2RR | GGCTAC |  |
| Barcode-2SF | GTGAAA |  |
| Barcode-2SR | GTGGCC |  |
| Barcode-2TF | GAGTGG |  |
| Barcode-2TR | GGTAGC |  |
| Barcode-2UF | ATTCCT |  |
| Barcode-2UR | CAAAAG |  |
| Barcode-2VF | CACGAT |  |
| Barcode-2VR | CACTCA |  |
| GA-F1 | TTGTAAAACGACGGCCAGTGAATTCAGTAATTCATCCAGGTCAC | Multiple sgRNA assembly |
| GA-R1 | ATGAATTACTAGACATAAAAAACAAAAAAAGGG |  |
| GA-F2 | TTTTATGTCTAGTAATTCATCCAGGTCAC |  |
| GA-R2 | CTATGACCATGATTACGCCAAGCTTAGACATAAAAAACAAAAAAAGCAC |  |
| GA-R3 | CTATGACCATGATTACGCCAAGCTTAGACATAAAAAACAAAAAAAGGGA |  |
| GA-F4 | AAAACGACGGCCAGTGCCAAGCTTAGTAATTCATCCAGGTCACCAAGTT |  |
| GA-R4 | GCACTGCAGGCATGCAAGCTTAGACATAAAAAACAAAAAAAGCACCGAC |  |
| *OsALS*-T2-OT1-F | GCCGCCTGCACCCGCGACAG | Potential off-target sites analysis |
| *OsALS*-T2-OT1-R | CGATGGGCGTCTCCTGGAACGCG |  |
| *OsALS*-T2-OT2-F | GGTGCTCGACGGTGTCGGTG |  |
| *OsALS*-T2-OT2-R | GTTGTGCTTGGTGATGGAGCG |  |
| *OsALS*-T2-OT3-F | TTAGCGACGCACCGGCTCG |  |
| *OsALS*-T2-OT3-R | CAGAGGGAGCATGTCCTTACCG |  |
| *OsALS*-T2-OT4-F | AAGATCATGGCGACGGAGCTG |  |
| *OsALS*-T2-OT4-R | TTGACTGGAGCGCGTCGTCG |  |
| OsBADH2-Indels-sgL-OT1-F | CCAGAATATCCAAACCGATGGATAGAGC |  |
| OsBADH2-Indels-sgL-OT1-R | CATTTTATGCGTCTCCCGAGGC |  |
| OsBADH2-Indels-sgL-OT2-F | CGATCCGTTTGTGGGTGTGAC |  |
| OsBADH2-Indels-sgL-OT2-R | CGCGTGGAAGCCCATGACAG |  |
| OsBADH2-Indels-sgL-OT3-F | GCTCTCTGACCAAAGCGGCG |  |
| OsBADH2-Indels-sgL-OT3-R | GATTCGAGATTATGTGCTAACATCGCC |  |
| OsBADH2-Indels-sgL-OT4-F | TGGCGGTGAACCCCGACGATG |  |
| OsBADH2-Indels-sgL-OT4-R | GGTAATTAGCTGATGGACACTCCTTCC |  |
| OsBADH2-Indels-sgL-OT5-F | GCCATGACAAACGCTGTGCG |  |
| OsBADH2-Indels-sgL-OT5-R | GAGGGAGTCGGCGTGTAGG |  |
| OsBADH2-Indels-sgL-OT6-F | AGTCGTGGAGGTGCACGATG |  |
| OsBADH2-Indels-sgL-OT6-R | CGCATGCACACCTGAGTTTGG |  |
| OsBADH2-Indels-sgL-OT7-F | GGAGGGAGCTTTGACTTTCCC |  |
| OsBADH2-Indels-sgL-OT7-R | CCGCCTCCATCACCATTCCC |  |
| OsBADH2-Indels-sgL-OT8-F | CCGAGTCCGACAGCTATGGC |  |
| OsBADH2-Indels-sgL-OT8-R | GCGTCGATGGCGATGCTGC |  |
| OsBADH2-Indels-sgR-OT1-F | AGAGCAGATTGCTCACCGTGAC |  |
| OsBADH2-Indels-sgR-OT1-R | CGGTTAAGCTCGTGCTCGAG |  |
| OsBADH2-Indels-sgR-OT2-F | GGACTCACCTGCGTCGATAGC |  |
| OsBADH2-Indels-sgR-OT2-R | ACTCTTGAGTAACTATAGATGCCGGTTC |  |
| OsBADH2-Indels-sgR-OT3-F | AGCTCGACTGTTTCGTTGCG |  |
| OsBADH2-Indels-sgR-OT3-R | GCGGTTGAGCTCGTGCTCG |  |
| OsBADH2-Indels-sgR-OT4-F | TCCCGGCCACCTCTACTGG |  |
| OsBADH2-Indels-sgR-OT4-R | CGACGCCGAAGTCGGCGAG |  |
