# Supplementary material for: SWISS: multiplexed orthogonal genome editing in plants with a Cas9 nickase and engineered CRISPR RNA scaffolds
Source: Genome Biol. 2020 Jun 16;21:141. doi: 10.1186/s13059-020-02051-x (PMC7296638; doi:10.1186/s13059-020-02051-x)
Supplement: Supplementary file 2 — Additional file 2: Sequences S1. DNA sequences of the OsU3 and TaU6 promoters used in this study. Sequences S2. DNA sequences of sgRNA, esgRNA, and scRNAs used in this study. Sequences S3. DNA sequences of the modules composed of PBEcs and PABEcs used in this study. Sequences S4. DNA sequences of templates for assemble multiple sgRNAs used in this study. Sequences S5. DNA sequences of SWISSv2/v3 and SWISSv2/v3-NG used in this study. [file 13059_2020_2051_MOESM2_ESM.docx]

**Sequences S1.** DNA sequences of the *OsU3* and *TaU6* promoters used in this study.

**>pOsU3**

AGTAATTCATCCAGGTCACCAAGTTCTAGGATTTTCAGAACTGCAACTTATTTTATCAAGGAATCTTTAAACATACGAACAGATCACTTAAAGTTCTTCTGAAGCAACTTAAAGTTATCAGGCATGCATGGATCTTGGAGGAATCAGATGTGCAGTCAGGGACCATAGCACAAGACAGGCGTCTTCTACTGGTGCTACCAGCAAATGCTGGAAGCCGGGAACACTGGGTACGTTGGAAACCACGTGATGTGAAGAAGTAAGATAAACTGTAGGAGAAAAGCATTTCGTAGTGGGCCATGAAGCCTTTCAGGACATGTATTGCAGTATGGGCCGGCCCATTACGCAATTGGACGACAACAAAGACTAGTATTAGTACCACCTCGGCTATCCACATAGATCAAAGCTGATTTAAAAGAGTTGTGCAGATGATCCGTGGCG

**>pTaU6**

GACCAAGCCCGTTATTCTGACAGTTCTGGTGCTCAACACATTTATATTTATCAAGGAGCACATTGTTACTCACTGCTAGGAGGGAATCGAACTAGGAATATTGATCAGAGGAACTACGAGAGAGCTGAAGATAACTGCCCTCTAGCTCTCACTGATCTGGGCGCATAGTGAGATGCAGCCCACGTGAGTTCAGCAACGGTCTAGCGCTGGGCTTTTAGGCCCGCATGATCGGGCTTTGTCGGGTGGTCGACGTGTTCACGATTGGGGAGAGCAACGCAGCAGTTCCTCTTAGTTTAGTCCCACCTCGCCTGTCCAGCAGAGTTCTGACCGGTTTATAAACTCGCTTGCTGCATCAGACTTG

**Sequences S2.** DNA sequences of sgRNA, esgRNA, and scRNAs used in this study.

**>sgRNA**

GTTTTAGAGCTAGAAATAGCAAGTTAAAATAAGGCTAGTCCGTTATCAACTTGAAAAAGTGGCACCGAGTCGGTGCTTTTTTTGTTTTTTATGTCT

**>esgRNA**

GTTTAAGAGCTATGCTGGAAACAGCATAGCAAGTTTAAATAAGGCTAGTCCGTTATCAACTTGAAAAAGTGGCACCGAGTCGGTGCTTTTTTTGTTTTTTATGTCT

**>sgRNA2.0**

GTTTTAGAGCTAGGCCAACATGAGGATCACCCATGTCTGCAGGGCCTAGCAAGTTAAAATAAGGCTAGTCCGTTATCAACTTGGCCAACATGAGGATCACCCATGTCTGCAGGGCCAAGTGGCACCGAGTCGGTGCTTTTTTTGTTTTTTATGTCT

**>sgRNA2.1**

GTTTAAGAGCTAGGCCAAGATGAGGATCACCCATGCCTGCAGGGCCTAGCAAGTTTAAATAAGGCTAGTCCGTTATCAACTTGGCCAACATGAGGATCACCCATGTCTGCAGGGCCAAGTGGCACCGAGTCGGTGCTTTTTTTGTTTTTTATGTCT

**>sgRNA2.2**

GTTTCAGAGCTAGGCCAAGATGAGGATCACCCATGCCTGCAGGGCCTAGCAAGTTGAAATAAGGCTAGTCCGTTATCAACTTGGCCAACATGAGGATCACCCATGTCTGCAGGGCCAAGTGGCACCGAGTCGGTGCTTTTTTTGTTTTTTATGTCT

**>sgRNA2.3**

GTTTAAGAGCTATGCTGAAGATGAGGATCACCCATGCCTGCAGCAGCATAGCAAGTTTAAATAAGGCTAGTCCGTTATCAACTTGGCCAACATGAGGATCACCCATGTCTGCAGGGCCAAGTGGCACCGAGTCGGTGCTTTTTTTGTTTTTTATGTCT

**>sgRNA2.4**

GTTTCAGAGCTATGCTGAAGATGAGGATCACCCATGCCTGCAGCAGCATAGCAAGTTGAAATAAGGCTAGTCCGTTATCAACTTGGCCAACATGAGGATCACCCATGTCTGCAGGGCCAAGTGGCACCGAGTCGGTGCTTTTTTTGTTTTTTATGTCT

**>sgRNA4.0**

GTTTTAGAGCTAGGCCAACATGAGGATCACCCATGTCTGCAGGGCCTAGCAAGTTAAAATAAGGCTAGTCCGTTATCAACTTGGCCAACATGAGGATCACCCATGTCTGCAGGGCCAAGTGGCACCGAGTCGGTGCGGGAGCACATGAGGATCACCCATGTGCCACGAGCGACATGAGGATCACCCATGTCGCTCGTGTTCCCTTTTTTTGTTTTTTATGTCT

**>sgRNA-2🞨MS2**

GTTTTAGAGCTAGAAATAGCAAGTTAAAATAAGGCTAGTCCGTTATCAACTTGAAAAAGTGGCACCGAGTCGGTGCGGGAGCACATGAGGATCACCCATGTGCCACGAGCGACATGAGGATCACCCATGTCGCTCGTGTTCCCTTTTTTTGTTTTTTATGTCT

**>esgRNA-1🞨MS2**

GTTTAAGAGCTATGCTGGAAACAGCATAGCAAGTTTAAATAAGGCTAGTCCGTTATCAACTTGAAAAAGTGGCACCGAGTCGGTGCGCGCACATGAGGATCACCCATGTGCTTTTTTTGTTTTTTATGTCT

**>esgRNA-2🞨MS2**

GTTTAAGAGCTATGCTGGAAACAGCATAGCAAGTTTAAATAAGGCTAGTCCGTTATCAACTTGAAAAAGTGGCACCGAGTCGGTGCGGGAGCACATGAGGATCACCCATGTGCCACGAGCGACATGAGGATCACCCATGTCGCTCGTGTTCCCTTTTTTTGTTTTTTATGTCT

**>esgRNA-MS2+f6**

GTTTAAGAGCTATGCTGGAAACAGCATAGCAAGTTTAAATAAGGCTAGTCCGTTATCAACTTGAAAAAGTGGCACCGAGTCGGTGCGGGAGCACATGAGGATCACCCATGTGCGACTCCCACAGTCACTGGGGAGTCTTCCCTTTTTTTGTTTTTTATGTCT

**>esgRNA-3🞨MS2**

GTTTAAGAGCTATGCTGGAAACAGCATAGCAAGTTTAAATAAGGCTAGTCCGTTATCAACTTGAAAAAGTGGCACCGAGTCGGTGCGGGAGCACATGAGGATCACCCATGTGCCACGAGCGACATGAGGATCACCCATGTCGCTCGTGGACTCTCGTGTCACATGAGGATCACCCATGTGACACGAGAGTCTTCCCTTTTTTTGTTTTTTATGTCT

**>sgRNA7-1**

GTTTGAGAGCTACCTAAGGAGTTTATATGGAAACCCTTAGGTAGCAAGTTCAAATAAGGCTAGTCCGTTATCAACTTTAAGGAGTTTATATGGAAACCCTTAAAGTGGCACCGAGTCGGTGCTTTTTTTGTTTTTTATGTCT

**>sgRNA7-2**

GTTTGAGAGCTACCGGAGCAGACGATATGGCGTCGCTCCGGTAGCAAGTTCAAATAAGGCTAGTCCGTTATCAACTTGGAGCAGACGATATGGCGTCGCTCCAAGTGGCACCGAGTCGGTGCTTTTTTTGTTTTTTATGTCT

**>esgRNA-1🞨PP7-1**

GTTTAAGAGCTATGCTGGAAACAGCATAGCAAGTTTAAATAAGGCTAGTCCGTTATCAACTTGAAAAAGTGGCACCGAGTCGGTGCAACATAAGGAGTTTATATGGAAACCCTTATGTTTTTTTGTTTTTTATGTCT

**>esgRNA-2🞨PP7-1**

GTTTAAGAGCTATGCTGGAAACAGCATAGCAAGTTTAAATAAGGCTAGTCCGTTATCAACTTGAAAAAGTGGCACCGAGTCGGTGCGGGAGCTAAGGAGTTTATATGGAAACCCTTAGCCTGCTGCGTAAGGAGTTTATATGGAAACCCTTACGCAGCAGTTCCCTTTTTTTGTTTTTTATGTCT

**>esgRNA-2🞨PP7-2**

GTTTAAGAGCTATGCTGGAAACAGCATAGCAAGTTTAAATAAGGCTAGTCCGTTATCAACTTGAAAAAGTGGCACCGAGTCGGTGCGGGAGCGGAGCAGACGATATGGCGTCGCTCCGCCTGCTGCGGGAGCAGACGATATGGCGTCGCTCCCGCAGCAGTTCCCTTTTTTTGTTTTTTATGTCT

**>sgRNAB.0**

GTTTGAGAGCTAGGGCCCTGAAGAAGGGCCCTAGCAAGTTCAAATAAGGCTAGTCCGTTATCAACTTGGGCCCTGAAGAAGGGCCCAAGTGGCACCGAGTCGGTGCTTTTTTTGTTTTTTATGTCT

**>esgRNA-2🞨boxB**

GTTTAAGAGCTATGCTGGAAACAGCATAGCAAGTTTAAATAAGGCTAGTCCGTTATCAACTTGAAAAAGTGGCACCGAGTCGGTGCGGGAGCGCCCTGAAGAAGGGCGCCTGCTGCGGCCCTGAAGAAGGGCCGCAGCAGTTCCCTTTTTTTGTTTTTTATGTCT

**>esgRNA-1🞨com**

GTTTAAGAGCTATGCTGGAAACAGCATAGCAAGTTTAAATAAGGCTAGTCCGTTATCAACTTGAAAAAGTGGCACCGAGTCGGTGCCTGAATGCCTGCGAGCATCTTTTTTTGTTTTTTATGTCT

**>esgRNA-2🞨com**

GTTTAAGAGCTATGCTGGAAACAGCATAGCAAGTTTAAATAAGGCTAGTCCGTTATCAACTTGAAAAAGTGGCACCGAGTCGGTGCGGGAGCCTGAATGCCTGCGAGCATCGCCTGCTGCGCTGAATGCCTGCGAGCATCCGCAGCAGTTCCCTTTTTTTGTTTTTTATGTCT

**Sequences S3.** DNA sequences of the modules composed of PBEcs and PABEcs used in this study.

**>nCas9 (D10A)**

GACAAGAAGTACTCGATCGGCCTCGCCATCGGGACGAACTCAGTTGGCTGGGCCGTGATCACCGACGAGTACAAGGTGCCCTCTAAGAAGTTCAAGGTCCTGGGGAACACCGACCGCCATTCCATCAAGAAGAACCTCATCGGCGCTCTCCTGTTCGACAGCGGGGAGACCGCTGAGGCTACGAGGCTCAAGAGAACCGCTAGGCGCCGGTACACGAGAAGGAAGAACAGGATCTGCTACCTCCAAGAGATTTTCTCCAACGAGATGGCCAAGGTTGACGATTCATTCTTCCACCGCCTGGAGGAGTCTTTCCTCGTGGAGGAGGATAAGAAGCACGAGCGGCATCCCATCTTCGGCAACATCGTGGACGAGGTTGCCTACCACGAGAAGTACCCTACGATCTACCATCTGCGGAAGAAGCTCGTGGACTCCACCGATAAGGCGGACCTCAGACTGATCTACCTCGCTCTGGCCCACATGATCAAGTTCCGCGGCCATTTCCTGATCGAGGGGGATCTCAACCCAGACAACAGCGATGTTGACAAGCTGTTCATCCAACTCGTGCAGACCTACAACCAACTCTTCGAGGAGAACCCGATCAACGCCTCTGGCGTGGACGCGAAGGCTATCCTGTCCGCGAGGCTCTCGAAGTCCAGGAGGCTGGAGAACCTGATCGCTCAGCTCCCAGGCGAGAAGAAGAACGGCCTGTTCGGGAACCTCATCGCTCTCAGCCTGGGGCTCACCCCGAACTTCAAGTCGAACTTCGATCTCGCTGAGGACGCCAAGCTGCAACTCTCCAAGGACACCTACGACGATGACCTCGATAACCTCCTGGCCCAGATCGGCGATCAATACGCGGACCTGTTCCTCGCTGCCAAGAACCTGTCGGACGCCATCCTCCTGTCAGATATCCTCCGCGTGAACACCGAGATCACGAAGGCTCCACTCTCTGCCTCCATGATCAAGCGCTACGACGAGCACCATCAGGATCTGACCCTCCTGAAGGCGCTGGTCCGCCAACAGCTCCCGGAGAAGTACAAGGAGATTTTCTTCGATCAGTCGAAGAACGGCTACGCTGGGTACATCGACGGCGGGGCCTCACAAGAGGAGTTCTACAAGTTCATCAAGCCAATCCTGGAGAAGATGGACGGCACGGAGGAGCTCCTGGTGAAGCTCAACAGGGAGGACCTCCTGCGGAAGCAGAGAACCTTCGATAACGGCAGCATCCCCCACCAAATCCATCTCGGGGAGCTGCACGCCATCCTGAGAAGGCAAGAGGACTTCTACCCTTTCCTCAAGGATAACCGGGAGAAGATCGAGAAGATCCTGACCTTCAGAATCCCATACTACGTCGGCCCTCTCGCGCGGGGGAACTCAAGATTCGCTTGGATGACCCGCAAGTCTGAGGAGACCATCACGCCGTGGAACTTCGAGGAGGTGGTGGACAAGGGCGCTAGCGCTCAGTCGTTCATCGAGAGGATGACCAACTTCGACAAGAACCTGCCCAACGAGAAGGTGCTCCCTAAGCACTCGCTCCTGTACGAGTACTTCACCGTCTACAACGAGCTCACGAAGGTGAAGTACGTCACCGAGGGCATGCGCAAGCCAGCGTTCCTGTCCGGGGAGCAGAAGAAGGCTATCGTGGACCTCCTGTTCAAGACCAACCGGAAGGTCACGGTTAAGCAACTCAAGGAGGACTACTTCAAGAAGATCGAGTGCTTCGATTCGGTCGAGATCAGCGGCGTTGAGGACCGCTTCAACGCCAGCCTCGGGACCTACCACGATCTCCTGAAGATCATCAAGGATAAGGACTTCCTGGACAACGAGGAGAACGAGGATATCCTGGAGGACATCGTGCTGACCCTCACGCTGTTCGAGGACAGGGAGATGATCGAGGAGCGCCTGAAGACGTACGCCCATCTCTTCGATGACAAGGTCATGAAGCAACTCAAGCGCCGGAGATACACCGGCTGGGGGAGGCTGTCCCGCAAGCTCATCAACGGCATCCGGGACAAGCAGTCCGGGAAGACCATCCTCGACTTCCTCAAGAGCGATGGCTTCGCCAACAGGAACTTCATGCAACTGATCCACGATGACAGCCTCACCTTCAAGGAGGATATCCAAAAGGCTCAAGTGAGCGGCCAGGGGGACTCGCTGCACGAGCATATCGCGAACCTCGCTGGCTCCCCCGCGATCAAGAAGGGCATCCTCCAGACCGTGAAGGTTGTGGACGAGCTCGTGAAGGTCATGGGCCGGCACAAGCCTGAGAACATCGTCATCGAGATGGCCAGAGAGAACCAAACCACGCAGAAGGGGCAAAAGAACTCTAGGGAGCGCATGAAGCGCATCGAGGAGGGCATCAAGGAGCTGGGGTCCCAAATCCTCAAGGAGCACCCAGTGGAGAACACCCAACTGCAGAACGAGAAGCTCTACCTGTACTACCTCCAGAACGGCAGGGATATGTACGTGGACCAAGAGCTGGATATCAACCGCCTCAGCGATTACGACGTCGATCATATCGTTCCCCAGTCTTTCCTGAAGGATGACTCCATCGACAACAAGGTCCTCACCAGGTCGGACAAGAACCGCGGCAAGTCAGATAACGTTCCATCTGAGGAGGTCGTTAAGAAGATGAAGAACTACTGGAGGCAGCTCCTGAACGCCAAGCTGATCACGCAAAGGAAGTTCGACAACCTCACCAAGGCTGAGAGAGGCGGGCTCTCAGAGCTGGACAAGGCCGGCTTCATCAAGCGGCAGCTGGTCGAGACCAGACAAATCACGAAGCACGTTGCGCAAATCCTCGACTCTCGGATGAACACGAAGTACGATGAGAACGACAAGCTGATCAGGGAGGTTAAGGTGATCACCCTGAAGTCTAAGCTCGTCTCCGACTTCAGGAAGGATTTCCAGTTCTACAAGGTTCGCGAGATCAACAACTACCACCATGCCCATGACGCTTACCTCAACGCTGTGGTCGGCACCGCTCTGATCAAGAAGTACCCAAAGCTGGAGTCCGAGTTCGTGTACGGGGACTACAAGGTTTACGATGTGCGCAAGATGATCGCCAAGTCGGAGCAAGAGATCGGCAAGGCTACCGCCAAGTACTTCTTCTACTCAAACATCATGAACTTCTTCAAGACCGAGATCACGCTGGCCAACGGCGAGATCCGGAAGAGACCGCTCATCGAGACCAACGGCGAGACGGGGGAGATCGTGTGGGACAAGGGCAGGGATTTCGCGACCGTCCGCAAGGTTCTCTCCATGCCCCAGGTGAACATCGTCAAGAAGACCGAGGTCCAAACGGGCGGGTTCTCAAAGGAGTCTATCCTGCCTAAGCGGAACAGCGACAAGCTCATCGCCAGAAAGAAGGACTGGGACCCAAAGAAGTACGGCGGGTTCGACAGCCCTACCGTGGCCTACTCGGTCCTGGTTGTGGCGAAGGTTGAGAAGGGCAAGTCCAAGAAGCTCAAGAGCGTGAAGGAGCTCCTGGGGATCACCATCATGGAGAGGTCCAGCTTCGAGAAGAACCCAATCGACTTCCTGGAGGCCAAGGGCTACAAGGAGGTGAAGAAGGACCTGATCATCAAGCTCCCGAAGTACTCTCTCTTCGAGCTGGAGAACGGCAGGAAGAGAATGCTGGCTTCCGCTGGCGAGCTCCAGAAGGGGAACGAGCTCGCGCTGCCAAGCAAGTACGTGAACTTCCTCTACCTGGCTTCCCACTACGAGAAGCTCAAGGGCAGCCCGGAGGACAACGAGCAAAAGCAGCTGTTCGTCGAGCAGCACAAGCATTACCTCGACGAGATCATCGAGCAAATCTCCGAGTTCAGCAAGCGCGTGATCCTCGCCGACGCGAACCTGGATAAGGTCCTCTCCGCCTACAACAAGCACCGGGACAAGCCCATCAGAGAGCAAGCGGAGAACATCATCCATCTCTTCACCCTGACGAACCTCGGCGCTCCTGCTGCTTTCAAGTACTTCGACACCACGATCGATCGGAAGAGATACACCTCCACGAAGGAGGTCCTGGACGCGACCCTCATCCACCAGTCGATCACCGGCCTGTACGAGACGAGGATCGACCTCTCACAACTCGGCGGGGAT

**>APOBEC1**

TCATCGGAGACCGGCCCTGTTGCTGTTGACCCCACCCTGCGGCGGAGAATCGAGCCACACGAGTTCGAGGTGTTCTTCGACCCAAGGGAGCTCCGCAAGGAGACGTGCCTCCTGTACGAGATCAACTGGGGCGGCAGGCACTCCATCTGGAGGCACACCAGCCAAAACACCAACAAGCACGTGGAGGTCAACTTCATCGAGAAGTTCACCACCGAGAGGTACTTCTGCCCAAACACCCGCTGCTCCATCACCTGGTTCCTGTCCTGGAGCCCATGCGGCGAGTGCTCCAGGGCCATCACCGAGTTCCTCAGCCGCTACCCACACGTCACCCTGTTCATCTACATCGCCAGGCTCTACCACCACGCCGACCCAAGGAACAGGCAGGGCCTCCGCGACCTGATCTCCAGCGGCGTGACCATCCAAATCATGACCGAGCAGGAGTCCGGCTACTGCTGGAGGAACTTCGTCAACTACTCCCCAAGCAACGAGGCCCACTGGCCAAGGTACCCACACCTCTGGGTGCGCCTCTACGTGCTCGAGCTGTACTGCATCATCCTCGGCCTGCCACCATGCCTCAACATCCTGAGGCGCAAGCAACCACAGCTGACCTTCTTCACCATCGCCCTCCAAAGCTGCCACTACCAGAGGCTCCCACCACACATCCTGTGGGCTACCGGCCTCAAG

**>APOBEC1-YE1**

TCATCGGAAACCGGCCCTGTTGCTGTTGACCCCACCCTGCGGCGGAGAATCGAGCCACACGAGTTCGAGGTGTTCTTCGACCCAAGGGAGCTCCGCAAGGAGACGTGCCTCCTGTACGAGATCAACTGGGGCGGCAGGCACTCCATCTGGAGGCACACCAGCCAAAACACCAACAAGCACGTGGAGGTCAACTTCATCGAGAAGTTCACCACCGAGAGGTACTTCTGCCCAAACACCCGCTGCTCCATCACCTGGTTCCTGTCCTACAGCCCATGCGGCGAGTGCTCCAGGGCCATCACCGAGTTCCTCAGCCGCTACCCACACGTCACCCTGTTCATCTACATCGCCAGGCTCTACCACCACGCCGACCCAGAGAACAGGCAGGGCCTCCGCGACCTGATCTCCAGCGGCGTGACCATCCAAATCATGACCGAGCAGGAGTCCGGCTACTGCTGGAGGAACTTCGTCAACTACTCCCCAAGCAACGAGGCCCACTGGCCAAGGTACCCACACCTCTGGGTGCGCCTCTACGTGCTCGAGCTGTACTGCATCATCCTCGGCCTGCCACCATGCCTCAACATCCTGAGGCGCAAGCAACCACAGCTGACCTTCTTCACCATCGCCCTCCAAAGCTGCCACTACCAGAGGCTCCCACCACACATCCTGTGGGCTACCGGCCTCAAG

**>APOBEC1-YE2**

TCATCGGAAACCGGCCCTGTTGCTGTTGACCCCACCCTGCGGCGGAGAATCGAGCCACACGAGTTCGAGGTGTTCTTCGACCCAAGGGAGCTCCGCAAGGAGACGTGCCTCCTGTACGAGATCAACTGGGGCGGCAGGCACTCCATCTGGAGGCACACCAGCCAAAACACCAACAAGCACGTGGAGGTCAACTTCATCGAGAAGTTCACCACCGAGAGGTACTTCTGCCCAAACACCCGCTGCTCCATCACCTGGTTCCTGTCCTACAGCCCATGCGGCGAGTGCTCCAGGGCCATCACCGAGTTCCTCAGCCGCTACCCACACGTCACCCTGTTCATCTACATCGCCAGGCTCTACCACCACGCCGACCCAAGGAACAGGCAGGGCCTTGAGGACCTGATCTCCAGCGGCGTGACCATCCAAATCATGACCGAGCAGGAGTCCGGCTACTGCTGGAGGAACTTCGTCAACTACTCCCCAAGCAACGAGGCCCACTGGCCAAGGTACCCACACCTCTGGGTGCGCCTCTACGTGCTCGAGCTGTACTGCATCATCCTCGGCCTGCCACCATGCCTCAACATCCTGAGGCGCAAGCAACCACAGCTGACCTTCTTCACCATCGCCCTCCAAAGCTGCCACTACCAGAGGCTCCCACCACACATCCTGTGGGCTACCGGCCTCAAG

**>APOBEC1-EE**

TCATCGGAAACCGGCCCTGTTGCTGTTGACCCCACCCTGCGGCGGAGAATCGAGCCACACGAGTTCGAGGTGTTCTTCGACCCAAGGGAGCTCCGCAAGGAGACGTGCCTCCTGTACGAGATCAACTGGGGCGGCAGGCACTCCATCTGGAGGCACACCAGCCAAAACACCAACAAGCACGTGGAGGTCAACTTCATCGAGAAGTTCACCACCGAGAGGTACTTCTGCCCAAACACCCGCTGCTCCATCACCTGGTTCCTGTCCTGGAGCCCATGCGGCGAGTGCTCCAGGGCCATCACCGAGTTCCTCAGCCGCTACCCACACGTCACCCTGTTCATCTACATCGCCAGGCTCTACCACCACGCCGACCCAGAGAACAGGCAGGGCCTCGAGGACCTGATCTCCAGCGGCGTGACCATCCAAATCATGACCGAGCAGGAGTCCGGCTACTGCTGGAGGAACTTCGTCAACTACTCCCCAAGCAACGAGGCCCACTGGCCAAGGTACCCACACCTCTGGGTGCGCCTCTACGTGCTCGAGCTGTACTGCATCATCCTCGGCCTGCCACCATGCCTCAACATCCTGAGGCGCAAGCAACCACAGCTGACCTTCTTCACCATCGCCCTCCAAAGCTGCCACTACCAGAGGCTCCCACCACACATCCTGTGGGCTACCGGCCTCAAG

**>APOBEC1-YEE**

TCATCGGAAACCGGCCCTGTTGCTGTTGACCCCACCCTGCGGCGGAGAATCGAGCCACACGAGTTCGAGGTGTTCTTCGACCCAAGGGAGCTCCGCAAGGAGACGTGCCTCCTGTACGAGATCAACTGGGGCGGCAGGCACTCCATCTGGAGGCACACCAGCCAAAACACCAACAAGCACGTGGAGGTCAACTTCATCGAGAAGTTCACCACCGAGAGGTACTTCTGCCCAAACACCCGCTGCTCCATCACCTGGTTCCTGTCCTACAGCCCATGCGGCGAGTGCTCCAGGGCCATCACCGAGTTCCTCAGCCGCTACCCACACGTCACCCTGTTCATCTACATCGCCAGGCTCTACCACCACGCCGACCCAGAGAACAGGCAGGGCCTCGAGGACCTGATCTCCAGCGGCGTGACCATCCAAATCATGACCGAGCAGGAGTCCGGCTACTGCTGGAGGAACTTCGTCAACTACTCCCCAAGCAACGAGGCCCACTGGCCAAGGTACCCACACCTCTGGGTGCGCCTCTACGTGCTCGAGCTGTACTGCATCATCCTCGGCCTGCCACCATGCCTCAACATCCTGAGGCGCAAGCAACCACAGCTGACCTTCTTCACCATCGCCCTCCAAAGCTGCCACTACCAGAGGCTCCCACCACACATCCTGTGGGCTACCGGCCTCAAG

**>UGI**

ACCAACCTGTCCGACATCATCGAGAAGGAGACGGGCAAGCAACTCGTGATCCAGGAGAGCATCCTCATGCTGCCAGAGGAGGTGGAGGAGGTCATCGGCAACAAGCCAGAGTCCGACATCCTGGTGCACACCGCCTACGACGAGTCCACCGACGAGAACGTCATGCTCCTGACCAGCGACGCCCCAGAGTACAAGCCATGGGCCCTCGTCATCCAGGACAGCAACGGGGAGAACAAGATCAAGATGCTG

**>ecTadA**

TCCGAGGTGGAGTTCTCTCACGAGTATTGGATGAGGCACGCTCTTACACTTGCTAAGAGAGCTTGGGACGAAAGAGAAGTGCCAGTTGGCGCCGTTCTTGTGCATAATAATAGGGTGATCGGCGAGGGTTGGAATAGACCAATTGGAAGGCATGATCCAACAGCTCACGCAGAGATTATGGCTCTCAGACAAGGCGGCCTCGTTATGCAGAACTACAGGCTCATTGACGCTACACTCTACGTGACACTCGAACCTTGCGTTATGTGCGCCGGAGCTATGATTCATTCTAGGATTGGCAGGGTCGTGTTTGGAGCTAGGGACGCTAAAACAGGAGCCGCCGGATCTCTTATGGACGTGTTGCATCATCCAGGCATGAACCATAGGGTGGAGATTACAGAGGGCATTCTTGCAGACGAGTGCGCTGCTCTTCTTTCCGATTTCTTCAGGATGAGAAGGCAGGAGATTAAGGCCCAGAAGAAGGCTCAGTCTTCTACAGAT

**>ecTadA7.10**

TCTGAAGTGGAGTTCTCCCACGAGTATTGGATGAGGCACGCTCTTACACTTGCTAAAAGGGCTAGGGACGAAAGGGAAGTTCCAGTTGGAGCTGTTCTCGTGCTCAATAACAGGGTGATTGGCGAGGGTTGGAATAGAGCCATTGGACTCCATGATCCAACAGCTCACGCAGAGATTATGGCTCTTAGACAAGGCGGCCTCGTTATGCAGAATTACAGACTCATCGACGCCACACTCTACGTTACCTTCGAACCTTGCGTTATGTGCGCCGGAGCTATGATCCATTCTAGGATTGGCAGGGTCGTGTTCGGCGTTAGAAACGCTAAGACAGGAGCTGCAGGCTCTCTTATGGACGTTCTTCATTACCCAGGCATGAATCATAGAGTGGAGATCACAGAAGGCATTCTTGCAGACGAGTGCGCAGCTCTCCTTTGCTATTTCTTCAGGATGCCGAGGCAAGTTTTCAACGCTCAGAAGAAGGCCCAGTCTTCTACAGAT

**>MCP**

GCCTCAAACTTCACCCAGTTCGTGCTCGTCGACAATGGCGGAACCGGCGATGTGACAGTCGCACCTTCCAACTTCGCCAATGGCATCGCGGAGTGGATTTCCAGCAACTCCAGGTCCCAGGCGTACAAGGTGACCTGCTCCGTCAGGCAGTCATCTGCGCAGAATCGCAAGTACACAATCAAGGTGGAGGTCCCTAAGGGCGCCTGGAGGTCCTACCTCAACATGGAGCTGACCATCCCGATTTTCGCCACAAATAGCGACTGCGAGCTGATCGTGAAGGCGATGCAGGGCCTCCTGAAGGATGGCAACCCGATCCCATCAGCGATTGCCGCGAATTCTGGCATCTAC

**>PCP**

TCCAAGACAATCGTGCTCAGCGTCGGAGAGGCAACCCGCACACTGACCGAGATCCAGTCCACCGCGGATCGGCAGATTTTCGAGGAGAAAGTGGGACCTCTCGTCGGAAGGCTCAGGCTGACAGCCAGCCTGAGGCAGAACGGCGCAAAGACCGCGTACAGGGTGAATCTCAAGCTGGACCAGGCAGATGTGGTCGACTCCGGCCTCCCAAAGGTGAGGTACACACAGGTCTGGTCACACGACGTGACAATTGTCGCCAACTCTACCGAGGCGTCCCGCAAGAGCCTCTACGATCTGACAAAGTCACTCGTGGCCACCTCTCAGGTCGAGGACCTGGTGGTCAATCTCGTCCCGCTGGGCCGG

**>N22p**

GGCAACGCCAGGACCAGGAGGAGGGAGAGGAGGGCCGAGAAGCAGGCCCAGTGGAAGGCCGCCAAC

**>COM**

AAGAGCATCAGGTGCAAGAACTGCAACAAGCTGCTGTTCAAGGCCGACAGCTTCGACCACATCGAGATCAGGTGCCCGAGGTGCAAGAGGCACATCATCATGCTGAACGCCTGCGAGCACCCGACCGAGAAGCACTGCGGCAAGAGGGAGAAGATCACCCACAGCGACGAGACGGTGAGGTAC

**>T2A**

GAGGGCAGAGGAAGTCTTCTAACATGCGGTGACGTGGAGGAGAATCCCGGCCCT

**>Nucleoplasmin NLS**

AAGCGGCCAGCGGCGACGAAGAAGGCGGGGCAGGCGAAGAAGAAGAAG

**>SV40 NLS**

CCAAAGAAGAAGCGGAAGGTG

**>XTEN linker**

TCCGGCAGCGAGACGCCAGGCACCTCCGAGAGCGCTACGCCTGAATCC

**>32 amino acids linker ((SGGS)_2_-XTEN-(SGGS)_2_)_1**

AGCGGAGGATCTTCCGGAGGATCTAGCGGCTCCGAGACACCAGGAACATCCGAAAGCGCTACACCAGAATCTAGCGGAGGCTCTTCCGGAGGATCT

**>32 amino acids linker ((SGGS)_2_-XTEN-(SGGS)_2_)_2**

TCCGGCGGATCTTCTGGAGGATCTAGCGGCTCCGAGACACCAGGAACATCCGAATCCGCTACACCAGAGTCTTCTGGAGGATCTAGCGGAGGATCT

**Sequences S4.** DNA sequences of templates for assemble multiple sgRNAs used in this study.

**>esgRNA-pTaU6**

GTTTAAGAGCTATGCTGGAAACAGCATAGCAAGTTTAAATAAGGCTAGTCCGTTATCAACTTGAAAAAGTGGCACCGAGTCGGTGCTTTTTTTGTTTTTTATGTCTGACCAAGCCCGTTATTCTGACAGTTCTGGTGCTCAACACATTTATATTTATCAAGGAGCACATTGTTACTCACTGCTAGGAGGGAATCGAACTAGGAATATTGATCAGAGGAACTACGAGAGAGCTGAAGATAACTGCCCTCTAGCTCTCACTGATCTGGGTCGCATAGTGAGATGCAGCCCACGTGAGTTCAGCAACGGTCTAGCGCTGGGCTTTTAGGCCCGCATGATCGGGCTTTTGTCGGGTGGTCGACGTGTTCACGATTGGGGAGAGCAACGCAGCAGTTCCTCTTAGTTTAGTCCCACCTCGCCTGTCCAGCAGAGTTCTGACCGGTTTATAAACTCGCTTGCTGCATCAGACTTG

**>esgRNA-2🞨boxB-pTaU6**

GTTTAAGAGCTATGCTGGAAACAGCATAGCAAGTTTAAATAAGGCTAGTCCGTTATCAACTTGAAAAAGTGGCACCGAGTCGGTGCgggagcGCCCTGAAGAAGGGCgcctgctgcgGCCCTGAAGAAGGGCcgcagcagttcccTTTTTTTGTTTTTTATGTCTGACCAAGCCCGTTATTCTGACAGTTCTGGTGCTCAACACATTTATATTTATCAAGGAGCACATTGTTACTCACTGCTAGGAGGGAATCGAACTAGGAATATTGATCAGAGGAACTACGAGAGAGCTGAAGATAACTGCCCTCTAGCTCTCACTGATCTGGGTCGCATAGTGAGATGCAGCCCACGTGAGTTCAGCAACGGTCTAGCGCTGGGCTTTTAGGCCCGCATGATCGGGCTTTTGTCGGGTGGTCGACGTGTTCACGATTGGGGAGAGCAACGCAGCAGTTCCTCTTAGTTTAGTCCCACCTCGCCTGTCCAGCAGAGTTCTGACCGGTTTATAAACTCGCTTGCTGCATCAGACTTG

**Sequences S5.** DNA sequences of SWISSv2/v3 and SWISSv2/v3-NG used in this study. The codon optimized nCas9 (D10A), nCas9-NG (D10A), linker, NLS, T2A, MCP, APOBEC1, UGI, ecTadA, ecTadA7.10, and N22p are highlighted in blue, wathet blue, gray, golden, orange, reseda green, green, black, crimson, purple, and pink red, respectively. The underlined and italic sequences indicate the sites of restriction enzymes used for constructing the vector. The red and bold sequences indicate the codons of R1335V/L1111R/D1135V/G1218R/E1219F/A1322R/T1337R variant sites in nCas9-NG (D10A).

**>SWISSv2/v3 (nCas9 (D10A)-3🞨NLS-T2A-MCP-XTEN linker-NLS-APOBEC1-UGI-NLS-T2A-ecTadA-32aa linker-ecTadA7.10-32aa linker-N22p-3🞨NLS)**

ATGGACAAGAAGTACTCGATCGGCCTCGCCATTGGGACTAACTCTGTTGGCTGGGCCGTGATCACCGACGAGTACAAGGTGCCCTCAAAGAAGTTCAAGGTCCTGGGCAACACCGATCGGCATTCCATCAAGAAGAATCTCATTGGCGCTCTCCTGTTCGACAGCGGCGAGACGGCTGAGGCTACGCGGCTCAAGCGCACCGCCCGCAGGCGGTACACGCGCAGGAAGAATCGCATCTGCTACCTGCAGGAGATTTTCTCCAACGAGATGGCGAAGGTTGACGATTCTTTCTTCCACAGGCTGGAGGAGTCATTCCTCGTGGAGGAGGATAAGAAGCACGAGCGGCATCCAATCTTCGGCAACATTGTCGACGAGGTTGCCTACCACGAGAAGTACCCTACGATCTACCATCTGCGGAAGAAGCTCGTGGACTCCACAGATAAGGCGGACCTCCGCCTGATCTACCTCGCTCTGGCCCACATGATTAAGTTCAGGGGCCATTTCCTGATCGAGGGGGATCTCAACCCGGACAATAGCGATGTTGACAAGCTGTTCATCCAGCTCGTGCAGACGTACAACCAGCTCTTCGAGGAGAACCCCATTAATGCGTCAGGCGTCGACGCGAAGGCTATCCTGTCCGCTAGGCTCTCGAAGTCTCGGCGCCTCGAGAACCTGATCGCCCAGCTGCCGGGCGAGAAGAAGAACGGCCTGTTCGGGAATCTCATTGCGCTCAGCCTGGGGCTCACGCCCAACTTCAAGTCGAATTTCGATCTCGCTGAGGACGCCAAGCTGCAGCTCTCCAAGGACACATACGACGATGACCTGGATAACCTCCTGGCCCAGATCGGCGATCAGTACGCGGACCTGTTCCTCGCTGCCAAGAATCTGTCGGACGCCATCCTCCTGTCTGATATTCTCAGGGTGAACACCGAGATTACGAAGGCTCCGCTCTCAGCCTCCATGATCAAGCGCTACGACGAGCACCATCAGGATCTGACCCTCCTGAAGGCGCTGGTCAGGCAGCAGCTCCCCGAGAAGTACAAGGAGATCTTCTTCGATCAGTCGAAGAACGGCTACGCTGGGTACATTGACGGCGGGGCCTCTCAGGAGGAGTTCTACAAGTTCATCAAGCCGATTCTGGAGAAGATGGACGGCACGGAGGAGCTGCTGGTGAAGCTCAATCGCGAGGACCTCCTGAGGAAGCAGCGGACATTCGATAACGGCAGCATCCCACACCAGATTCATCTCGGGGAGCTGCACGCTATCCTGAGGAGGCAGGAGGACTTCTACCCTTTCCTCAAGGATAACCGCGAGAAGATCGAGAAGATTCTGACTTTCAGGATCCCGTACTACGTCGGCCCACTCGCTAGGGGCAACTCCCGCTTCGCTTGGATGACCCGCAAGTCAGAGGAGACGATCACGCCGTGGAACTTCGAGGAGGTGGTCGACAAGGGCGCTAGCGCTCAGTCGTTCATCGAGAGGATGACGAATTTCGACAAGAACCTGCCAAATGAGAAGGTGCTCCCTAAGCACTCGCTCCTGTACGAGTACTTCACAGTCTACAACGAGCTGACTAAGGTGAAGTATGTGACCGAGGGCATGAGGAAGCCGGCTTTCCTGTCTGGGGAGCAGAAGAAGGCCATCGTGGACCTCCTGTTCAAGACCAACCGGAAGGTCACGGTTAAGCAGCTCAAGGAGGACTACTTCAAGAAGATTGAGTGCTTCGATTCGGTCGAGATCTCTGGCGTTGAGGACCGCTTCAACGCCTCCCTGGGGACCTACCACGATCTCCTGAAGATCATTAAGGATAAGGACTTCCTGGACAACGAGGAGAATGAGGATATCCTCGAGGACATTGTGCTGACACTCACTCTGTTCGAGGACCGGGAGATGATCGAGGAGCGCCTGAAGACTTACGCCCATCTCTTCGATGACAAGGTCATGAAGCAGCTCAAGAGGAGGAGGTACACCGGCTGGGGGAGGCTGAGCAGGAAGCTCATCAACGGCATTCGGGACAAGCAGTCCGGGAAGACGATCCTCGACTTCCTGAAGAGCGATGGCTTCGCGAACCGCAATTTCATGCAGCTGATTCACGATGACAGCCTCACATTCAAGGAGGATATCCAGAAGGCTCAGGTGAGCGGCCAGGGGGACTCGCTGCACGAGCATATCGCGAACCTCGCTGGCTCGCCAGCTATCAAGAAGGGGATTCTGCAGACCGTGAAGGTTGTGGACGAGCTGGTGAAGGTCATGGGCAGGCACAAGCCTGAGAACATCGTCATTGAGATGGCCCGGGAGAATCAGACCACGCAGAAGGGCCAGAAGAACTCACGCGAGAGGATGAAGAGGATCGAGGAGGGCATTAAGGAGCTGGGGTCCCAGATCCTCAAGGAGCACCCGGTGGAGAACACGCAGCTGCAGAATGAGAAGCTCTACCTGTACTACCTCCAGAATGGCCGCGATATGTATGTGGACCAGGAGCTGGATATTAACAGGCTCAGCGATTACGACGTCGATCATATCGTTCCACAGTCATTCCTGAAGGATGACTCCATTGACAACAAGGTCCTCACCAGGTCGGACAAGAACCGGGGCAAGTCTGATAATGTTCCTTCAGAGGAGGTCGTTAAGAAGATGAAGAACTACTGGCGCCAGCTCCTGAATGCCAAGCTGATCACGCAGCGGAAGTTCGATAACCTCACAAAGGCTGAGAGGGGCGGGCTCTCTGAGCTGGACAAGGCGGGCTTCATCAAGAGGCAGCTGGTCGAGACACGGCAGATCACTAAGCACGTTGCGCAGATTCTCGACTCACGGATGAACACTAAGTACGATGAGAATGACAAGCTGATCCGCGAGGTGAAGGTCATCACCCTGAAGTCAAAGCTCGTCTCCGACTTCAGGAAGGATTTCCAGTTCTACAAGGTTCGGGAGATCAACAATTACCACCATGCCCATGACGCGTACCTGAACGCGGTGGTCGGCACAGCTCTGATCAAGAAGTACCCAAAGCTCGAGAGCGAGTTCGTGTACGGGGACTACAAGGTTTACGATGTGAGGAAGATGATCGCCAAGTCGGAGCAGGAGATTGGCAAGGCTACCGCCAAGTACTTCTTCTACTCTAACATTATGAATTTCTTCAAGACAGAGATCACTCTGGCCAATGGCGAGATCCGGAAGCGCCCCCTCATCGAGACGAACGGCGAGACGGGGGAGATCGTGTGGGACAAGGGCAGGGATTTCGCGACCGTCAGGAAGGTTCTCTCCATGCCACAAGTGAATATCGTCAAGAAGACAGAGGTCCAGACTGGCGGGTTCTCTAAGGAGTCAATTCTGCCTAAGCGGAACAGCGACAAGCTCATCGCCCGCAAGAAGGACTGGGATCCGAAGAAGTACGGCGGGTTCGACAGCCCCACTGTGGCCTACTCGGTCCTGGTTGTGGCGAAGGTTGAGAAGGGCAAGTCCAAGAAGCTCAAGAGCGTGAAGGAGCTGCTGGGGATCACGATTATGGAGCGCTCCAGCTTCGAGAAGAACCCGATCGATTTCCTGGAGGCGAAGGGCTACAAGGAGGTGAAGAAGGACCTGATCATTAAGCTCCCCAAGTACTCACTCTTCGAGCTGGAGAACGGCAGGAAGCGGATGCTGGCTTCCGCTGGCGAGCTGCAGAAGGGGAACGAGCTGGCTCTGCCGTCCAAGTATGTGAACTTCCTCTACCTGGCCTCCCACTACGAGAAGCTCAAGGGCAGCCCCGAGGACAACGAGCAGAAGCAGCTGTTCGTCGAGCAGCACAAGCATTACCTCGACGAGATCATTGAGCAGATTTCCGAGTTCTCCAAGCGCGTGATCCTGGCCGACGCGAATCTGGATAAGGTCCTCTCCGCGTACAACAAGCACCGCGACAAGCCAATCAGGGAGCAGGCTGAGAATATCATTCATCTCTTCACCCTGACGAACCTCGGCGCCCCTGCTGCTTTCAAGTACTTCGACACAACTATCGATCGCAAGAGGTACACAAGCACTAAGGAGGTCCTGGACGCGACCCTCATCCACCAGTCGATTACCGGCCTCTACGAGACGCGCATCGACCTGTCTCAGCTCGGGGGCGACTCCGGCGGCAGCCCAAAGAAGAAGCGGAAGGTGTCTGGAGGTTCTCCTAAGAAAAAGAGAAAAGTGTCCGGCGGCTCCCCGAAGAAGAAGCGCAAGGTG*GAATTC*GAGGGCAGAGGAAGTCTTCTAACATGCGGTGACGTGGAGGAGAATCCCGGCCCTGCCTCAAACTTCACCCAGTTCGTGCTCGTCGACAATGGCGGAACCGGCGATGTGACAGTCGCACCTTCCAACTTCGCCAATGGCATCGCGGAGTGGATTTCCAGCAACTCCAGGTCCCAGGCGTACAAGGTGACCTGCTCCGTCAGGCAGTCATCTGCGCAGAATCGCAAGTACACAATCAAGGTGGAGGTCCCTAAGGGCGCCTGGAGGTCCTACCTCAACATGGAGCTGACCATCCCGATTTTCGCCACAAATAGCGACTGCGAGCTGATCGTGAAGGCGATGCAGGGCCTCCTGAAGGATGGCAACCCGATCCCATCAGCGATTGCCGCGAATTCTGGCATCTACTCCGGCAGCGAGACGCCAGGCACCTCCGAGAGCGCTACGCCTGAATCCCCAAAGAAGAAGAGGAAGGTTTCATCGGAaACCGGCCCTGTTGCTGTTGACCCCACCCTGCGGCGGAGAATCGAGCCACACGAGTTCGAGGTGTTCTTCGACCCAAGGGAGCTCCGCAAGGAGACGTGCCTCCTGTACGAGATCAACTGGGGCGGCAGGCACTCCATCTGGAGGCACACCAGCCAAAACACCAACAAGCACGTGGAGGTCAACTTCATCGAGAAGTTCACCACCGAGAGGTACTTCTGCCCAAACACCCGCTGCTCCATCACCTGGTTCCTGTCCTGGAGCCCATGCGGCGAGTGCTCCAGGGCCATCACCGAGTTCCTCAGCCGCTACCCACACGTCACCCTGTTCATCTACATCGCCAGGCTCTACCACCACGCCGACCCAAGGAACAGGCAGGGCCTCCGCGACCTGATCTCCAGCGGCGTGACCATCCAAATCATGACCGAGCAGGAGTCCGGCTACTGCTGGAGGAACTTCGTCAACTACTCCCCAAGCAACGAGGCCCACTGGCCAAGGTACCCACACCTCTGGGTGCGCCTCTACGTGCTCGAGCTGTACTGCATCATCCTCGGCCTGCCACCATGCCTCAACATCCTGAGGCGCAAGCAACCACAGCTGACCTTCTTCACCATCGCCCTCCAAAGCTGCCACTACCAGAGGCTCCCACCACACATCCTGTGGGCTACCGGCCTCAAGTCGGGGGGGAGCACCAACCTGTCCGACATCATCGAGAAGGAGACGGGCAAGCAACTCGTGATCCAGGAGAGCATCCTCATGCTGCCAGAGGAGGTGGAGGAGGTCATCGGCAACAAGCCAGAGTCCGACATCCTGGTGCACACCGCCTACGACGAGTCCACCGACGAGAACGTCATGCTCCTGACCAGCGACGCCCCAGAGTACAAGCCATGGGCCCTCGTCATCCAGGACAGCAACGGGGAGAACAAGATCAAGATGCTGTCGGGGGGGAGCAAGCGGCCAGCGGCGACGAAGAAGGCGGGGCAGGCGAAGAAGAAGAAGGAGGGCAGAGGAAGTCTTCTAACATGCGGTGACGTGGAGGAGAATCCCGGCCCTTCCGAGGTGGAGTTCTCTCACGAGTATTGGATGAGGCACGCTCTTACACTTGCTAAGAGAGCTTGGGACGAAAGAGAAGTGCCAGTTGGCGCCGTTCTTGTGCATAATAATAGGGTGATCGGCGAGGGTTGGAATAGACCAATTGGAAGGCATGATCCAACAGCTCACGCAGAGATTATGGCTCTCAGACAAGGCGGCCTCGTTATGCAGAACTACAGGCTCATTGACGCTACACTCTACGTGACACTCGAACCTTGCGTTATGTGCGCCGGAGCTATGATTCATTCTAGGATTGGCAGGGTCGTGTTTGGAGCTAGGGACGCTAAAACAGGAGCCGCCGGATCTCTTATGGACGTGTTGCATCATCCAGGCATGAACCATAGGGTGGAGATTACAGAGGGCATTCTTGCAGACGAGTGCGCTGCTCTTCTTTCCGATTTCTTCAGGATGAGAAGGCAGGAGATTAAGGCCCAGAAGAAGGCTCAGTCTTCTACAGATAGCGGAGGATCTTCCGGAGGATCTAGCGGCTCCGAGACACCAGGAACATCCGAAAGCGCTACACCAGAATCTAGCGGAGGCTCTTCCGGAGGATCTTCTGAAGTGGAGTTCTCCCACGAGTATTGGATGAGGCACGCTCTTACACTTGCTAAAAGGGCTAGGGACGAAAGGGAAGTTCCAGTTGGAGCTGTTCTCGTGCTCAATAACAGGGTGATTGGCGAGGGTTGGAATAGAGCCATTGGACTCCATGATCCAACAGCTCACGCAGAGATTATGGCTCTTAGACAAGGCGGCCTCGTTATGCAGAATTACAGACTCATCGACGCCACACTCTACGTTACCTTCGAACCTTGCGTTATGTGCGCCGGAGCTATGATCCATTCTAGGATTGGCAGGGTCGTGTTCGGCGTTAGAAACGCTAAGACAGGAGCTGCAGGCTCTCTTATGGACGTTCTTCATTACCCAGGCATGAATCATAGAGTGGAGATCACAGAAGGCATTCTTGCAGACGAGTGCGCAGCTCTCCTTTGCTATTTCTTCAGGATGCCGAGGCAAGTTTTCAACGCTCAGAAGAAGGCCCAGTCTTCTACAGATTCCGGCGGATCTTCCGGAGGATCTAGCGGCTCCGAGACACCAGGAACATCCGAATCCGCTACACCAGAGTCTTCTGGAGGATCTAGCGGAGGATCTGGCAACGCCAGGACCAGGAGGAGGGAGAGGAGGGCCGAGAAGCAGGCCCAGTGGAAGGCCGCCAACTCCGGCGGCAGCCCAAAGAAGAAGCGGAAGGTGTCTGGAGGTTCTCCTAAGAAAAAGAGAAAAGTGTCCGGCGGCTCCCCGAAGAAGAAGCGCAAGGTGTAG

**>SWISSv2/v3-NG (nCas9-NG (D10A)-3🞨NLS-T2A-MCP-XTEN linker-NLS-APOBEC1-UGI-NLS-T2A-ecTadA-32aa linker-ecTadA7.10-32aa linker-N22p-3🞨NLS)**

ATGGACAAGAAGTACTCGATCGGCCTCGCCATTGGGACTAACTCTGTTGGCTGGGCCGTGATCACCGACGAGTACAAGGTGCCCTCAAAGAAGTTCAAGGTCCTGGGCAACACCGATCGGCATTCCATCAAGAAGAATCTCATTGGCGCTCTCCTGTTCGACAGCGGCGAGACGGCTGAGGCTACGCGGCTCAAGCGCACCGCCCGCAGGCGGTACACGCGCAGGAAGAATCGCATCTGCTACCTGCAGGAGATTTTCTCCAACGAGATGGCGAAGGTTGACGATTCTTTCTTCCACAGGCTGGAGGAGTCATTCCTCGTGGAGGAGGATAAGAAGCACGAGCGGCATCCAATCTTCGGCAACATTGTCGACGAGGTTGCCTACCACGAGAAGTACCCTACGATCTACCATCTGCGGAAGAAGCTCGTGGACTCCACAGATAAGGCGGACCTCCGCCTGATCTACCTCGCTCTGGCCCACATGATTAAGTTCAGGGGCCATTTCCTGATCGAGGGGGATCTCAACCCGGACAATAGCGATGTTGACAAGCTGTTCATCCAGCTCGTGCAGACGTACAACCAGCTCTTCGAGGAGAACCCCATTAATGCGTCAGGCGTCGACGCGAAGGCTATCCTGTCCGCTAGGCTCTCGAAGTCTCGGCGCCTCGAGAACCTGATCGCCCAGCTGCCGGGCGAGAAGAAGAACGGCCTGTTCGGGAATCTCATTGCGCTCAGCCTGGGGCTCACGCCCAACTTCAAGTCGAATTTCGATCTCGCTGAGGACGCCAAGCTGCAGCTCTCCAAGGACACATACGACGATGACCTGGATAACCTCCTGGCCCAGATCGGCGATCAGTACGCGGACCTGTTCCTCGCTGCCAAGAATCTGTCGGACGCCATCCTCCTGTCTGATATTCTCAGGGTGAACACCGAGATTACGAAGGCTCCGCTCTCAGCCTCCATGATCAAGCGCTACGACGAGCACCATCAGGATCTGACCCTCCTGAAGGCGCTGGTCAGGCAGCAGCTCCCCGAGAAGTACAAGGAGATCTTCTTCGATCAGTCGAAGAACGGCTACGCTGGGTACATTGACGGCGGGGCCTCTCAGGAGGAGTTCTACAAGTTCATCAAGCCGATTCTGGAGAAGATGGACGGCACGGAGGAGCTGCTGGTGAAGCTCAATCGCGAGGACCTCCTGAGGAAGCAGCGGACATTCGATAACGGCAGCATCCCACACCAGATTCATCTCGGGGAGCTGCACGCTATCCTGAGGAGGCAGGAGGACTTCTACCCTTTCCTCAAGGATAACCGCGAGAAGATCGAGAAGATTCTGACTTTCAGGATCCCGTACTACGTCGGCCCACTCGCTAGGGGCAACTCCCGCTTCGCTTGGATGACCCGCAAGTCAGAGGAGACGATCACGCCGTGGAACTTCGAGGAGGTGGTCGACAAGGGCGCTAGCGCTCAGTCGTTCATCGAGAGGATGACGAATTTCGACAAGAACCTGCCAAATGAGAAGGTGCTCCCTAAGCACTCGCTCCTGTACGAGTACTTCACAGTCTACAACGAGCTGACTAAGGTGAAGTATGTGACCGAGGGCATGAGGAAGCCGGCTTTCCTGTCTGGGGAGCAGAAGAAGGCCATCGTGGACCTCCTGTTCAAGACCAACCGGAAGGTCACGGTTAAGCAGCTCAAGGAGGACTACTTCAAGAAGATTGAGTGCTTCGATTCGGTCGAGATCTCTGGCGTTGAGGACCGCTTCAACGCCTCCCTGGGGACCTACCACGATCTCCTGAAGATCATTAAGGATAAGGACTTCCTGGACAACGAGGAGAATGAGGATATCCTCGAGGACATTGTGCTGACACTCACTCTGTTCGAGGACCGGGAGATGATCGAGGAGCGCCTGAAGACTTACGCCCATCTCTTCGATGACAAGGTCATGAAGCAGCTCAAGAGGAGGAGGTACACCGGCTGGGGGAGGCTGAGCAGGAAGCTCATCAACGGCATTCGGGACAAGCAGTCCGGGAAGACGATCCTCGACTTCCTGAAGAGCGATGGCTTCGCGAACCGCAATTTCATGCAGCTGATTCACGATGACAGCCTCACATTCAAGGAGGATATCCAGAAGGCTCAGGTGAGCGGCCAGGGGGACTCGCTGCACGAGCATATCGCGAACCTCGCTGGCTCGCCAGCTATCAAGAAGGGGATTCTGCAGACCGTGAAGGTTGTGGACGAGCTGGTGAAGGTCATGGGCAGGCACAAGCCTGAGAACATCGTCATTGAGATGGCCCGGGAGAATCAGACCACGCAGAAGGGCCAGAAGAACTCACGCGAGAGGATGAAGAGGATCGAGGAGGGCATTAAGGAGCTGGGGTCCCAGATCCTCAAGGAGCACCCGGTGGAGAACACGCAGCTGCAGAATGAGAAGCTCTACCTGTACTACCTCCAGAATGGCCGCGATATGTATGTGGACCAGGAGCTGGATATTAACAGGCTCAGCGATTACGACGTCGATCATATCGTTCCACAGTCATTCCTGAAGGATGACTCCATTGACAACAAGGTCCTCACCAGGTCGGACAAGAACCGGGGCAAGTCTGATAATGTTCCTTCAGAGGAGGTCGTTAAGAAGATGAAGAACTACTGGCGCCAGCTCCTGAATGCCAAGCTGATCACGCAGCGGAAGTTCGATAACCTCACAAAGGCTGAGAGGGGCGGGCTCTCTGAGCTGGACAAGGCGGGCTTCATCAAGAGGCAGCTGGTCGAGACACGGCAGATCACTAAGCACGTTGCGCAGATTCTCGACTCACGGATGAACACTAAGTACGATGAGAATGACAAGCTGATCCGCGAGGTGAAGGTCATCACCCTGAAGTCAAAGCTCGTCTCCGACTTCAGGAAGGATTTCCAGTTCTACAAGGTTCGGGAGATCAACAATTACCACCATGCCCATGACGCGTACCTGAACGCGGTGGTCGGCACAGCTCTGATCAAGAAGTACCCAAAGCTCGAGAGCGAGTTCGTGTACGGGGACTACAAGGTTTACGATGTGAGGAAGATGATCGCCAAGTCGGAGCAGGAGATTGGCAAGGCTACCGCCAAGTACTTCTTCTACTCTAACATTATGAATTTCTTCAAGACAGAGATCACTCTGGCCAATGGCGAGATCCGGAAGCGCCCCCTCATCGAGACGAACGGCGAGACGGGGGAGATCGTGTGGGACAAGGGCAGGGATTTCGCGACCGTCAGGAAGGTTCTCTCCATGCCACAAGTGAATATCGTCAAGAAGACAGAGGTCCAGACTGGCGGGTTCTCTAAGGAGTCAATT**CGG**CCTAAGCGGAACAGCGACAAGCTCATCGCCCGCAAGAAGGACTGGGATCCGAAGAAGTACGGCGGGTTC**GTC**AGCCCCACTGTGGCCTACTCGGTCCTGGTTGTGGCGAAGGTTGAGAAGGGCAAGTCCAAGAAGCTCAAGAGCGTGAAGGAGCTGCTGGGGATCACGATTATGGAGCGCTCCAGCTTCGAGAAGAACCCGATCGATTTCCTGGAGGCGAAGGGCTACAAGGAGGTGAAGAAGGACCTGATCATTAAGCTCCCCAAGTACTCACTCTTCGAGCTGGAGAACGGCAGGAAGCGGATGCTGGCTTCCGCT**CGCTTC**CTGCAGAAGGGGAACGAGCTGGCTCTGCCGTCCAAGTATGTGAACTTCCTCTACCTGGCCTCCCACTACGAGAAGCTCAAGGGCAGCCCCGAGGACAACGAGCAGAAGCAGCTGTTCGTCGAGCAGCACAAGCATTACCTCGACGAGATCATTGAGCAGATTTCCGAGTTCTCCAAGCGCGTGATCCTGGCCGACGCGAATCTGGATAAGGTCCTCTCCGCGTACAACAAGCACCGCGACAAGCCAATCAGGGAGCAGGCTGAGAATATCATTCATCTCTTCACCCTGACGAACCTCGGCGCCCCT**CGT**GCTTTCAAGTACTTCGACACAACTATCGATCGCAAG**GTA**TAC**AGA**AGCACTAAGGAGGTCCTGGACGCGACCCTCATCCACCAGTCGATTACCGGCCTCTACGAGACGCGCATCGACCTGTCTCAGCTCGGGGGCGACTCCGGCGGCAGCCCAAAGAAGAAGCGGAAGGTGTCTGGAGGTTCTCCTAAGAAAAAGAGAAAAGTGTCCGGCGGCTCCCCGAAGAAGAAGCGCAAGGTG*GAATTC*GAGGGCAGAGGAAGTCTTCTAACATGCGGTGACGTGGAGGAGAATCCCGGCCCTGCCTCAAACTTCACCCAGTTCGTGCTCGTCGACAATGGCGGAACCGGCGATGTGACAGTCGCACCTTCCAACTTCGCCAATGGCATCGCGGAGTGGATTTCCAGCAACTCCAGGTCCCAGGCGTACAAGGTGACCTGCTCCGTCAGGCAGTCATCTGCGCAGAATCGCAAGTACACAATCAAGGTGGAGGTCCCTAAGGGCGCCTGGAGGTCCTACCTCAACATGGAGCTGACCATCCCGATTTTCGCCACAAATAGCGACTGCGAGCTGATCGTGAAGGCGATGCAGGGCCTCCTGAAGGATGGCAACCCGATCCCATCAGCGATTGCCGCGAATTCTGGCATCTACTCCGGCAGCGAGACGCCAGGCACCTCCGAGAGCGCTACGCCTGAATCCCCAAAGAAGAAGAGGAAGGTTTCATCGGAaACCGGCCCTGTTGCTGTTGACCCCACCCTGCGGCGGAGAATCGAGCCACACGAGTTCGAGGTGTTCTTCGACCCAAGGGAGCTCCGCAAGGAGACGTGCCTCCTGTACGAGATCAACTGGGGCGGCAGGCACTCCATCTGGAGGCACACCAGCCAAAACACCAACAAGCACGTGGAGGTCAACTTCATCGAGAAGTTCACCACCGAGAGGTACTTCTGCCCAAACACCCGCTGCTCCATCACCTGGTTCCTGTCCTGGAGCCCATGCGGCGAGTGCTCCAGGGCCATCACCGAGTTCCTCAGCCGCTACCCACACGTCACCCTGTTCATCTACATCGCCAGGCTCTACCACCACGCCGACCCAAGGAACAGGCAGGGCCTCCGCGACCTGATCTCCAGCGGCGTGACCATCCAAATCATGACCGAGCAGGAGTCCGGCTACTGCTGGAGGAACTTCGTCAACTACTCCCCAAGCAACGAGGCCCACTGGCCAAGGTACCCACACCTCTGGGTGCGCCTCTACGTGCTCGAGCTGTACTGCATCATCCTCGGCCTGCCACCATGCCTCAACATCCTGAGGCGCAAGCAACCACAGCTGACCTTCTTCACCATCGCCCTCCAAAGCTGCCACTACCAGAGGCTCCCACCACACATCCTGTGGGCTACCGGCCTCAAGTCGGGGGGGAGCACCAACCTGTCCGACATCATCGAGAAGGAGACGGGCAAGCAACTCGTGATCCAGGAGAGCATCCTCATGCTGCCAGAGGAGGTGGAGGAGGTCATCGGCAACAAGCCAGAGTCCGACATCCTGGTGCACACCGCCTACGACGAGTCCACCGACGAGAACGTCATGCTCCTGACCAGCGACGCCCCAGAGTACAAGCCATGGGCCCTCGTCATCCAGGACAGCAACGGGGAGAACAAGATCAAGATGCTGTCGGGGGGGAGCAAGCGGCCAGCGGCGACGAAGAAGGCGGGGCAGGCGAAGAAGAAGAAGGAGGGCAGAGGAAGTCTTCTAACATGCGGTGACGTGGAGGAGAATCCCGGCCCTTCCGAGGTGGAGTTCTCTCACGAGTATTGGATGAGGCACGCTCTTACACTTGCTAAGAGAGCTTGGGACGAAAGAGAAGTGCCAGTTGGCGCCGTTCTTGTGCATAATAATAGGGTGATCGGCGAGGGTTGGAATAGACCAATTGGAAGGCATGATCCAACAGCTCACGCAGAGATTATGGCTCTCAGACAAGGCGGCCTCGTTATGCAGAACTACAGGCTCATTGACGCTACACTCTACGTGACACTCGAACCTTGCGTTATGTGCGCCGGAGCTATGATTCATTCTAGGATTGGCAGGGTCGTGTTTGGAGCTAGGGACGCTAAAACAGGAGCCGCCGGATCTCTTATGGACGTGTTGCATCATCCAGGCATGAACCATAGGGTGGAGATTACAGAGGGCATTCTTGCAGACGAGTGCGCTGCTCTTCTTTCCGATTTCTTCAGGATGAGAAGGCAGGAGATTAAGGCCCAGAAGAAGGCTCAGTCTTCTACAGATAGCGGAGGATCTTCCGGAGGATCTAGCGGCTCCGAGACACCAGGAACATCCGAAAGCGCTACACCAGAATCTAGCGGAGGCTCTTCCGGAGGATCTTCTGAAGTGGAGTTCTCCCACGAGTATTGGATGAGGCACGCTCTTACACTTGCTAAAAGGGCTAGGGACGAAAGGGAAGTTCCAGTTGGAGCTGTTCTCGTGCTCAATAACAGGGTGATTGGCGAGGGTTGGAATAGAGCCATTGGACTCCATGATCCAACAGCTCACGCAGAGATTATGGCTCTTAGACAAGGCGGCCTCGTTATGCAGAATTACAGACTCATCGACGCCACACTCTACGTTACCTTCGAACCTTGCGTTATGTGCGCCGGAGCTATGATCCATTCTAGGATTGGCAGGGTCGTGTTCGGCGTTAGAAACGCTAAGACAGGAGCTGCAGGCTCTCTTATGGACGTTCTTCATTACCCAGGCATGAATCATAGAGTGGAGATCACAGAAGGCATTCTTGCAGACGAGTGCGCAGCTCTCCTTTGCTATTTCTTCAGGATGCCGAGGCAAGTTTTCAACGCTCAGAAGAAGGCCCAGTCTTCTACAGATTCCGGCGGATCTTCCGGAGGATCTAGCGGCTCCGAGACACCAGGAACATCCGAATCCGCTACACCAGAGTCTTCTGGAGGATCTAGCGGAGGATCTGGCAACGCCAGGACCAGGAGGAGGGAGAGGAGGGCCGAGAAGCAGGCCCAGTGGAAGGCCGCCAACTCCGGCGGCAGCCCAAAGAAGAAGCGGAAGGTGTCTGGAGGTTCTCCTAAGAAAAAGAGAAAAGTGTCCGGCGGCTCCCCGAAGAAGAAGCGCAAGGTGTAG
