# Supplementary material for: SWISS: multiplexed orthogonal genome editing in plants with a Cas9 nickase and engineered CRISPR RNA scaffolds
Source: Genome Biol. 2020 Jun 16;21:141. doi: 10.1186/s13059-020-02051-x (PMC7296638; doi:10.1186/s13059-020-02051-x)
Supplement: Supplementary file 3 — Additional file 3. Review history. [file 13059_2020_2051_MOESM3_ESM.docx]

Review History

**First round of review**

**Reviewer 1**

**Were you able to assess all statistics in the manuscript, including the appropriateness of statistical tests used?**

No

**Were you able to directly test the methods?**

No

**Comments to author:**

In this manuscript, Li et al. developed a multiplexed platform to simultaneously induce different kinds of editing by using aptamer-containing sgRNAs to recruit different effector proteins.  This study is of potential interest to the gene editing field and may provide useful tools for functional genomics and molecular breeding in plants. The text and figures are carefully prepared and main results well described. Nevertheless, a few major concerns still remain and needs to be addressed.  Below are specific comments for the authors.

1. The authors nicely showed that SWISSv3 can be used to induce cytosine base editing, adenine base editing and indel formation simultaneously. However, SWISSv3 has many components and generally, the delivery efficiency is inversely correlated with the number of components, thereby leading to inefficient editing. In this regard, the editing efficiencies of cytosine base editing, adenine base editing and indel formation induced by SWISSv3 should be compared to the efficiency of each kind of editing individually at multiple sites, i.e. comparing the cytosine, adenine editing and indel formation efficiency of SWISSv3 at multiple sites to that induced by sgRNA/PBE, esgRNA/PABE or sgRNAs/Cas9 respectively.

2. The authors determined off-target effects at precited off-target sites and found no mutation was induced by SWISSv3. However, recent studies showed that CBEs can also induce Cas9-independent, genome-wide mutations. Thus, whether SWISSv3 also induces Cas9-independent, genome-wide off-target mutations should be checked and presented.

**Reviewer 2**

**Were you able to assess all statistics in the manuscript, including the appropriateness of statistical tests used?**

No

**Were you able to directly test the methods?**

No

**Comments to author:**

This project investigated quite comprehensively the use of RNA aptamers to recruit cytidine and adenine deaminases for base editing. The goal is to eventually develop a multiplex system for simultaneous C to T conversion at one site and A to G conversion at the other. In this sense, the goal was reached. With current base editing systems, it is very common that indels are generated due to mismatch repair of the nicked DNA. Also, pair nicking (whether use a pure nickase or a complex base editor) of course can introduce deletions and this is nothing new. The most useful implication of the so-called SWISS systems developed here is simultaneous C to T and A to G editing at different sites in an orthogonal way. I think the novelty is there. It is pity that the authors did not show a real application.

Other points:
1)     The authors somehow chose a less efficient cytidine deaminase to start with. The APOBEC1 based deaminase is one of the worse among deaminases of choice, when compared to PmCDA, hAID, hA3A, etc. Hence, even though the authors showed their PBEc4 had much higher base conversion rates than the original BE3, this PBEc4 may not have higher activity when compared to other BE3 types that use PmCDA or other cytidine deaminases. My question is why did not the authors try out these better deaminases to truly improve C to T base editing?
2)     Similarly, the new PABEc5, although novel in an aptamer setting, failed to outperform existing and conventional A to G base editing systems. This will seriously impact the usefulness of the system. Any idea for improvement?
3)     I like the authors' comprehensive way of study of RNA aptamers and their corresponding RNA binding proteins. However, the ways that the authors cited the literatures gave readers impression that no such endeavors were done in plants. Similarly, the authors' claim in lines 257-259 is slightly misleading because it gave people impression that none of such RNA aptamer-RNA binding protein systems tested in this study was applied in plants before. This of course is not true. I have come across multiple papers (both original and review papers) describing the use of similar RNA aptamers in CRISPR applications in plants. The authors should have cited those papers to give others some credits which also reflects the prior arts.
4)     Among the deletions induced, the authors should show the genotypes in sequence so that one can figure out whether those are truly induced by paired nicking or simply by the editing at one site with one sgRNA.
5)     For lines 262-265, the authors talked about direct evolution. What does this mean? Why is it special for SWISSS systems in this? If the authors concern about introducing random mutations, then using most efficient cytidine deaminase or adenine deaminase seems better than the SWISS systems.
6)     In multiplexed editing (Table 2), the C to T editing rates and A to G editing rates were all at a single digit percentage range, whether for single, double or triple editing events. Further, nearly all the base editing events contain base change plus indels, which really suggest the system is far from being pitched as a tool for orthogonal base editing. Really, it just generated indels at most target sites. I was disappointed to see the data because it just makes me skeptical on the usefulness of the system reported here. The authors should admit this and discuss ways for improvements.

**Authors Response**

**Point-by-point responses to the reviewers’ comments:**

Reviewer #1: In this manuscript, Li et al. developed a multiplexed platform to simultaneously induce different kinds of editing by using aptamer-containing sgRNAs to recruit different effector proteins. This study is of potential interest to the gene editing field and may provide useful tools for functional genomics and molecular breeding in plants. The text and figures are carefully prepared and main results well described. Nevertheless, a few major concerns still remain and needs to be addressed. Below are specific comments for the authors.
1. The authors nicely showed that SWISSv3 can be used to induce cytosine base editing, adenine base editing and indel formation simultaneously. However, SWISSv3 has many components and generally, the delivery efficiency is inversely correlated with the number of components, thereby leading to inefficient editing. In this regard, the editing efficiencies of cytosine base editing, adenine base editing and indel formation induced by SWISSv3 should be compared to the efficiency of each kind of editing individually at multiple sites, i.e. comparing the cytosine, adenine editing and indel formation efficiency of SWISSv3 at multiple sites to that induced by sgRNA/PBE, esgRNA/PABE or sgRNAs/Cas9 respectively.
*Response: Thanks for the reviewer’s suggestion. We have added the comparison data into Additional file 1: Figure S13 in the revised manuscript. Take the target sites of OsCDC48 and OsEP1-T1 as an example, we found that that C-to-T editing efficiency of SWISSv3 was higher on the PAM proximal positions (C9, 32.3%; C15, 0.3%), but lower on the distal positions (C5, 0.6%) than that of PBE (C9, 29.9%; C15, 0.1%; CT5, 2.9%) (Fig. 3e). The A-to-G editing efficiency of SWISSv3 (A4, 1.3%; A7, 1.2%) was slightly lower than that of PABE-2 (A4, 2.1%; A7, 2.1%), while the indel frequency was comparable between SWISSv3 (2.1%) and the paired nCas9 (D10A) (2.1%) (Additional file 1: Figure S13a). Similar results were also shown in Additional file 1: Figure S13b. These data support that SWISS is a reliable multi-functional genome editing tool. These results were combined into the revised manuscript in L188-L195.*

2. The authors determined off-target effects at precited off-target sites and found no mutation was induced by SWISSv3. However, recent studies showed that CBEs can also induce Cas9-independent, genome-wide mutations. Thus, whether SWISSv3 also induces Cas9-independent, genome-wide off-target mutations should be checked and presented.
*Response: Thanks for the reviewer’s suggestion. We have checked whether SWISSv3 induces Cas9-independent genome-wide off-target random mutations and added the WGS analysis in Figure 3f-i. Our results showed that the genome-wide Cas9-independent off-target effects of the SWISS system was comparable to that of PBE (L220-L227).*

Reviewer #2: This project investigated quite comprehensively the use of RNA aptamers to recruit cytidine and adenine deaminases for base editing. The goal is to eventually develop a multiplex system for simultaneous C to T conversion at one site and A to G conversion at the other. In this sense, the goal was reached. With current base editing systems, it is very common that indels are generated due to mismatch repair of the nicked DNA. Also, pair nicking (whether use a pure nickase or a complex base editor) of course can introduce deletions and this is nothing new. The most useful implication of the so-called SWISS systems developed here is simultaneous C to T and A to G editing at different sites in an orthogonal way. I think the novelty is there. It is pity that the authors did not show a real application.
*Response: Thanks for the reviewer’s suggestion. We agree that the most useful application of SWISS is to perform simultaneous C-to-T and A-to-G editing at different sites in an orthogonal way. The rice mutants that obtained in this manuscript implied the SWISS system indeed work in rice regenerated plants.*

Other points:
1) The authors somehow chose a less efficient cytidine deaminase to start with. The APOBEC1 based deaminase is one of the worse among deaminases of choice, when compared to PmCDA, hAID, hA3A, etc. Hence, even though the authors showed their PBEc4 had much higher base conversion rates than the original BE3, this PBEc4 may not have higher activity when compared to other BE3 types that use PmCDA or other cytidine deaminases. My question is why did not the authors try out these better deaminases to truly improve C to T base editing?
*Response: Thanks for the comments. The aim of the SWISS system was to perform orthogonal editing events of CBE, ABE, and/or indels by different RNA scaffolds. We used APOBEC1 to illustrate that cytosine base editor can be repurposed into RNA aptamer-recruiting architecture. In the near future, we will replace APOBEC1 with PmCDA, hAID, hA3A to improve the C>T editing efficiency.*

2) Similarly, the new PABEc5, although novel in an aptamer setting, failed to outperform existing and conventional A to G base editing systems. This will seriously impact the usefulness of the system. Any idea for improvement?
*Response: We agree with the reviewer that we therefore proposed several strategies to improve A>G editing efficiency in SWISS in the revised manuscript. One is to use the recently-evolved ecTadA variants with obviously A>G editing efficiency improvement, such as the adenosine deaminase in ABE8e (Richter, Nature Biotechnology, 2020) and ABE8.8/13/17/20 (Gaudelli, Nature Biotechnology, 2020). The other strategy is to fuse a synthetic transcription activation domain for opening chromatin to improve the A>G editing efficiency. The information has been added in L256-L260.*

3) I like the authors' comprehensive way of study of RNA aptamers and their corresponding RNA binding proteins. However, the ways that the authors cited the literatures gave readers impression that no such endeavors were done in plants. Similarly, the authors' claim in lines 257-259 is slightly misleading because it gave people impression that none of such RNA aptamer-RNA binding protein systems tested in this study was applied in plants before. This of course is not true. I have come across multiple papers (both original and review papers) describing the use of similar RNA aptamers in CRISPR applications in plants. The authors should have cited those papers to give others some credits which also reflects the prior arts.
*Response: Thanks for the comments. We have cited the following two relevant papers in the revised manuscript.
1. Li Z, Zhang D, Xiong X, Yan B, Xie W, Sheen J, Li J-F: A potent Cas9-derived gene activator for plant and mammalian cells. Nat Plants 2017, 3:930-936.
2. Lowder LG, Zhou J, Zhang Y, Malzahn A, Zhong Z, Hsieh T-F, Voytas DF, Zhang Y, Qi Y: Robust Transcriptional Activation in Plants Using Multiplexed CRISPR-Act2.0 and mTALE-Act Systems. Mol Plant 2018, 11:245-256.*

4) Among the deletions induced, the authors should show the genotypes in sequence so that one can figure out whether those are truly induced by paired nicking or simply by the editing at one site with one sgRNA.
*Response: Thanks for the comments. We have added the genotypes in sequence into Additional file 1: Figure S14c.*

5) For lines 262-265, the authors talked about direct evolution. What does this mean? Why is it special for SWISSS systems in this? If the authors concern about introducing random mutations, then using most efficient cytidine deaminase or adenine deaminase seems better than the SWISS systems.
*Response: Thanks for the comments. We have now deleted the comment since the SWISS systems developed here may not appropriate for directed evolution. We have remove these sentences in the revised manuscript.*

6) In multiplexed editing (Table 2), the C to T editing rates and A to G editing rates were all at a single digit percentage range, whether for single, double or triple editing events. Further, nearly all the base editing events contain base change plus indels, which really suggest the system is far from being pitched as a tool for orthogonal base editing. Really, it just generated indels at most target sites. I was disappointed to see the data because it just makes me skeptical on the usefulness of the system reported here. The authors should admit this and discuss ways for improvements.
*Response: Thanks for the comments. The aim of SWISSv3 is to introduce simultaneous CBE, ABE, and Indels editing on three different targets, respectively. The results in Table 2 showed that single, double, and triple mutants were obtained when using SWISSv3, while the efficiency of triple mutants was 7.3%. Moreover, the indels editing events were induced by paired sgRNAs in SWISSv3. When performing orthogonal base editing, the SWISSv2 should be best choice not SWISSv2.*

**Second round of review**

**Reviewer 1**

In this revised manuscript, the authors compared the editing efficiencies of cytosine base editing, adenosine base editing and indels formation induced by SWISSv3 to that induced by PBE, PABE-2 and paired nickase, respectively. Moreover, the authors also performed WGS analysis to check if SWISSv3 also induce Cas9-independent genome-wide off-target random mutations. As expected, the SWISSv3does induce genome-wide random mutation at a level comparable to that of PBE. The revised manuscript has been improved compared to the original version and I think the manuscript, in its revised form, is suitable for publishing in Genome Biology.

**Reviewer 2**

I have no more concerns for this work and I endorse its publication.
